# Epigenetic Alterations of DNA Methylation and miRNA Contribution to Lung Adenocarcinoma

**DOI:** 10.3389/fgene.2022.817552

**Published:** 2022-05-31

**Authors:** Wenhan Cai, Miao Jing, Jiaxin Wen, Hua Guo, Zhiqiang Xue

**Affiliations:** ^1^ Medical School of Chinese PLA, Beijing, China; ^2^ Department of Thoracic Surgery, The First Medical Centre, Chinese PLA General Hospital, Beijing, China

**Keywords:** DNA methylation, miRNA, epigenetics, lung adenocarcinoma, mRNA

## Abstract

This study focused on the epigenetic alterations of DNA methylation and miRNAs for lung adenocarcinoma (LUAD) diagnosis and treatment using bioinformatics analyses. DNA methylation data and mRNA and miRNA expression microarray data were obtained from The Cancer Genome Atlas (TCGA) database. The differentially methylated genes (DMGs), differentially expressed genes (DEGs), and differentially expressed miRNAs were analyzed by using the limma package. The DAVID database performed GO and KEGG pathway enrichment analyses. Using STRING and Cytoscape, we constructed the protein–protein interaction (PPI) network and achieved visualization. The online analysis tool CMap was used to identify potential small-molecule drugs for LUAD. In LUAD, 607 high miRNA-targeting downregulated genes and 925 low miRNA-targeting upregulated genes, as well as 284 hypermethylated low-expression genes and 315 hypomethylated high-expression genes, were obtained. They were mainly enriched in terms of pathways in cancer, neuroactive ligand–receptor interaction, cAMP signaling pathway, and cytosolic DNA-sensing pathway. In addition, 40 upregulated and 84 downregulated genes were regulated by both aberrant alternations of DNA methylation and miRNAs. Five small-molecule drugs were identified as a potential treatment for LUAD, and five hub genes (*SLC2A1*, *PAX6*, *LEP*, *KLF4*, and *FGF10*) were found in PPI, and two of them (*SLC2A1* and *KLF4*) may be related to the prognosis of LUAD. In summary, our study identified a series of differentially expressed genes associated with epigenetic alterations of DNA methylation and miRNA in LUAD. Five small-molecule drugs and five hub genes may be promising drugs and targets for LUAD treatment.

## 1 Introduction

Lung cancer is a fatal malignancy featuring the highest incidence (11.6%) and mortality (18.4%) globally. Lung adenocarcinoma (LUAD), increasing yearly, is the most common histological type of lung cancer (Bray et al., 2018). LUAD often arises peripherally and has adenoid tissue differentiation and mucin production in histology (Chen et al., 2014). It has been documented that the pathogenesis of LUAD involves inflammation, oxidative stress, mitochondrial dysfunction, changes in lipid metabolism, and epigenetic changes (Lindskog et al., 2014; Tan et al., 2016; Zhang C. et al., 2021). Unlike other subtypes, LUAD has a high rate of gene mutations. Although targeted therapy has improved the survival rate and quality of life of nearly 60% of LUAD patients with corresponding driver gene mutations in recent years, drug resistance is still inevitable. The long-term survival rate of LUAD is still not satisfactory (Kulasingam and Diamandis, 2008; Hirsch et al., 2017). A large amount of literature studies show that environmental factors and genetic and epigenetic factors will affect the occurrence and development of lung adenocarcinoma (Fabrizio et al., 2020; Gong et al., 2020; Wang et al., 2020; Huang et al., 2021; Shi et al., 2021). Although detailed knowledge about the processes of initiation and progression of LUAD is still unknown and remains a major stumbling block on the road to LUAD treatment, robust and accurate development of biomarkers will greatly facilitate early diagnosis and treatment of biological characteristics of LUAD. Therefore, there is an urgent need to identify new therapeutic targets and some chemicals of LUAD.

Carcinogenesis is a complex process involving genetic and epigenetic changes. Abnormal genetic and epigenetic changes are the hallmarks of cancer. Epigenetic modifications can modify the gene expression without altering the DNA sequence. In cancer, deviant epigenetic regulation includes miRNA gene silencing, DNA methylation, mRNA and non-coding RNA methylation, histone methylation, and histone acetylation (Maruyama et al., 2011). The aforementioned processes are closely related and affect protein synthesis. Interference with each operation may lead to dysfunction.

miRNAs are small non-coding RNA sequences about 19–23 nucleotides in length, which are highly conserved in regulating post-translational modifications (Bartel, 2004). miRNAs can exhibit carcinogenic effects or suppressor tumors by regulating target genes. These two miRNAs are termed oncomiR and tumor suppressor (TS) miRNA, respectively

miRNAs can show carcinogenic effects or suppressor tumors by regulating target genes. These two miRNAs are termed oncomiR and tumor suppressor (TS) miRNA, respectively (Zhang et al., 2014). They have emerged as promising biomarkers for diagnostic, therapeutic, and prognostic applications due to their association with LUAD (Gu et al., 2017; Wang et al., 2019; Yuan et al., 2019). For instance, miR-196b-5p displays high expressions, whereas its target gene RSPO2 (R-Spodin 2) is expressed low in the cancer tissues and normal in para-cancer tissues, promoting proliferation and migration and invasion of LUAD (Xu and Xu, 2020).

DNA methylation is a genetic modification that does not change the DNA sequence (Santos et al., 2005). DNA methylation is associated with the subtypes and prognosis of multiple tumors, including LUAD (Fleischer et al., 2017; Long et al., 2019; Ding et al., 2020; Xu et al., 2020). Shen et al. (2019) discovered that the hypermethylation of HOXA9 and hypomethylation of TULP2, CCND1, and KRTAP8-1 could be used as biomarkers for the early detection of LUAD in the undetermined lung nodules.

As yet, although a large number of studies have demonstrated the abnormal DNA methylations or the global methylation level and miRNA level in LUAD, the comprehensive regulatory network and pathways analyses of DNA methylation levels and miRNA epigenetic alterations have not yet been conducted.

This study systematically analyzed the data on DNA methylation microarrays, miRNA expression microarrays, and mRNA expression profiling microarrays from TCGA database to identify the core genes and pathways that lead to the occurrence and development of LUAD *via* epigenetic regulation.

## 2 Materials and Methods

### 2.1. Microarray Data

In this study, the data on DNA methylation microarrays (including 437 LUAD and 29 adjacent normal tissue samples), miRNA expression microarrays (including 483 LUAD and 45 adjacent normal tissue samples), and mRNA expression profiling microarrays (including 497 LUAD and 54 adjacent normal tissue samples) were obtained from TCGA database (https://portal.gdc.cancer.gov/).

### 2.2. Data Process

The Perl script (Perl version 5.18.4) was used to process expression data to obtain mRNA and miRNA matrix. R (version 4.0.2) and Bioconductor packages were used to preprocess the raw gene expression profiles, including background correction, normalization, and logarithmic conversion. Differentially methylated probes (DMPs), differentially expressed miRNAs (DEMs), and differentially expressed genes (DEGs) were performed by using the limma package in R. DMPs were screened with P. adjust <0.05 and ∣logFC | >0.2 as the cut-off criteria. DEMs were screened with P. adjust <0.05 and ∣logFC |>2 as the cut-off criteria, and DEGs were screened with P. adjust <0.05 and ∣logFC |>1 as the threshold. Draw Venn Diagram online software (http://bioinformatics.psb.ugent.be/webtools/Venn/) was used to find overlapping genes from DMPs, DEMs, and DEGs. Aberrant methylated and expressed genes were overlapped to obtain hypermethylated low-expression genes and hypomethylated high-expression genes. Subsequently, high miRNA-targeting downregulated genes and low miRNA-targeting upregulated genes were obtained *via* overlapping potential targets of DEMs and DEGs. The Kaplan–Meier plotter database (https://kmplot.com/analysis/index.php?p=service&cancer=lung) was used for the survival analysis of hub genes.

### 2.3. Prediction of Potential Targets of miRNAs and Construction of the miRNA–mRNA Network

The targets of DEGs were predicted by microT-CDS online software of DIANA TOOLS (http://diana.imis.athena-innovation.gr/DianaTools/index.php?r=microT_CDS/index) and the miRWalk database (http://mirwalk.umm.uni-heidelberg.de/). In addition, the Cytoscape tool (v3.7.2) was used to construct the entire miRNA–mRNA regulatory network.

### 2.4. Functional and Pathway Enrichment Analysis

Gene ontology (GO) analyses, including the biological process (BP), cellular component (CC), and molecular function (MF), were conducted for the upregulated genes, downregulated genes, hypermethylation-low-expression genes, and hypomethylation-high-expression genes selected by DAVID (https://david.ncifcrf.gov/). Subsequently, we performed the Kyoto Encyclopedia of Genes and Genomes (KEGG) pathway enrichment analyses for the high miRNA-targeting downregulated genes, low miRNA-targeting upregulated genes, hypermethylation-low-expression genes, and hypomethylation-high-expression genes. All analyses were performed with *p* < 0.05 as the screening condition.

### 2.5. Protein–Protein Interaction Network Construction and Module Analysis

We used the Search Tool of the Retrieval of Interacting Genes (STRING) online tool to perform PPI networks of hypermethylation-low-expression genes and hypomethylation-high-expression genes, respectively. cytoHubba in Cytoscape software was used to obtain hub genes within the PPI (top 10 nodes ranked by degree). The functional and pathway enrichment analysis of the genes in each module was performed by DAVID with *p* < 0.05 as the threshold.

### 2.6. Real-Time Quantitative PCR

PC9 and BEAS-2B cell lines were purchased from Zhejiang Meisen Cell Technology Co., Ltd. (MeisenCTCC). The PC9 cell line was cultured using 1640 + 10% FBS+1% anti-anti. The BEAS-2B cell line was cultured using BEGM, with 1% anti-anti added to the culture. Real-time Quantitative PCR was performed using Bio-Rad CFX96. After cell culture, the cells were washed three times with iced PBS. The RNA isolater Total RNA Extraction Reagent (Vazyme) was used to isolate the total RNA from cells. Then, 1 μg of total RNA and HiScript III-RT SuperMix for qPCR (Vazyme) were used for reverse transcription, according to the manufacturer’s instructions. Amplification reactions were set up in 20 μL volume containing ChamQ Universal SYBR qPCR Master Mix (Vazyme) and amplification primers according to the manufacturer’s instructions. The primer sequences used for real-time PCR are listed as follows. An amount of 5ng of cDNA was used in each amplification reaction.

The primer sequences for PCR amplification were as follows: SLC2A1, forward: 5′-TCT​GGC​ATC​AAC​GCT​GTC​TTC-3′ and reverse: 5′-CGA​TAC​CGG​AGC​CAA​TGG​T-3′; PAX6, forward: 5′-TGG​GCA​GGT​ATT​ACG​AGA​CTG-3′ and reverse: 5′-ACT​CCC​GCT​TAT​ACT​GGG​CTA-3′; LEP forward: 5′-TGC​CTT​CCA​GAA​ACG​TGA​TCC-3′ and reverse: 5′-CTC​TGT​GGA​GTA​GCC​TGA​AGC-3′; KLF4 forward: 5′-CGG​ACA​TCA​ACG​ACG​TGA​G-3′ and reverse: 5′-GAC​GCC​TTC​AGC​ACG​AAC​T-3′; FGF forward: 5′-CAG​TAG​AAA​TCG​GAG​TTG​TTG​CC-3′ and reverse: 5′-TGA​GCC​ATA​GAG​TTT​CCC​CTT​C-3′; and β actin forward: 5′-CAT​GTA​CGT​TGC​TAT​CCA​GGC-3′ and reverse: 5′-CTC​CTT​AAT​GTC​ACG​CAC​GAT-3′.

### 2.7. Drug Exploration in CMap

The Connectivity Map (CMap) database (https://www.broadinstitute.org/) contains gene expression profiles of human cells treated with small bioactive molecules. Researchers can use CMap to identify connections among small molecules that share a physiological process, chemicals, and actions and then predict the potential drugs (Lamb et al., 2006). We used the CMap database to identify potential small-molecule drugs that reverse or induce DEGs’ modified expression in LUAD cell lines (mean range from −0.5 to 0.5 and *p* < 0.01).

### 2.8. Statistical Analyses

All the results were analyzed and processed by GraphPad Prism 8 software. The unpaired *t*-test was used for statistical analysis, and the data were expressed as mean ± standard deviation. *p* < 0.05 was considered statistically significant; ^∗^ meant *p* < 0.05, ^∗∗^ meant *p* < 0.01, ^∗∗∗^ meant *p* < 0.001, and ^∗∗∗∗^ meant *p* < 0.0001.

## 3 Results

### 3.1. Identification of Abnormal Methylated Differentially Expressed Genes in LUAD

The characteristics of mRNA and miRNA transcriptome profiling and DNA methylation profiling based on the TCGA database are shown in Supplementary Table S1. In mRNA expression profiling microarrays of TCGA, a total of 12972 DEGs were screened in cancer tissue samples from LUAD, including 10076 upregulated genes and 2896 downregulated genes. Simultaneously, 13 high-expressed miRNAs and 18 low-expressed miRNAs were identified in miRNA expression microarrays of TCGA database. Supplementary Table S2 records the characteristics of the top five differentially expressed miRNAs and their potential target DEGs. As to DNA methylation microarrays, 2405 hypermethylated genes and 2155 hypomethylated genes were found.

**FIGURE 1:**
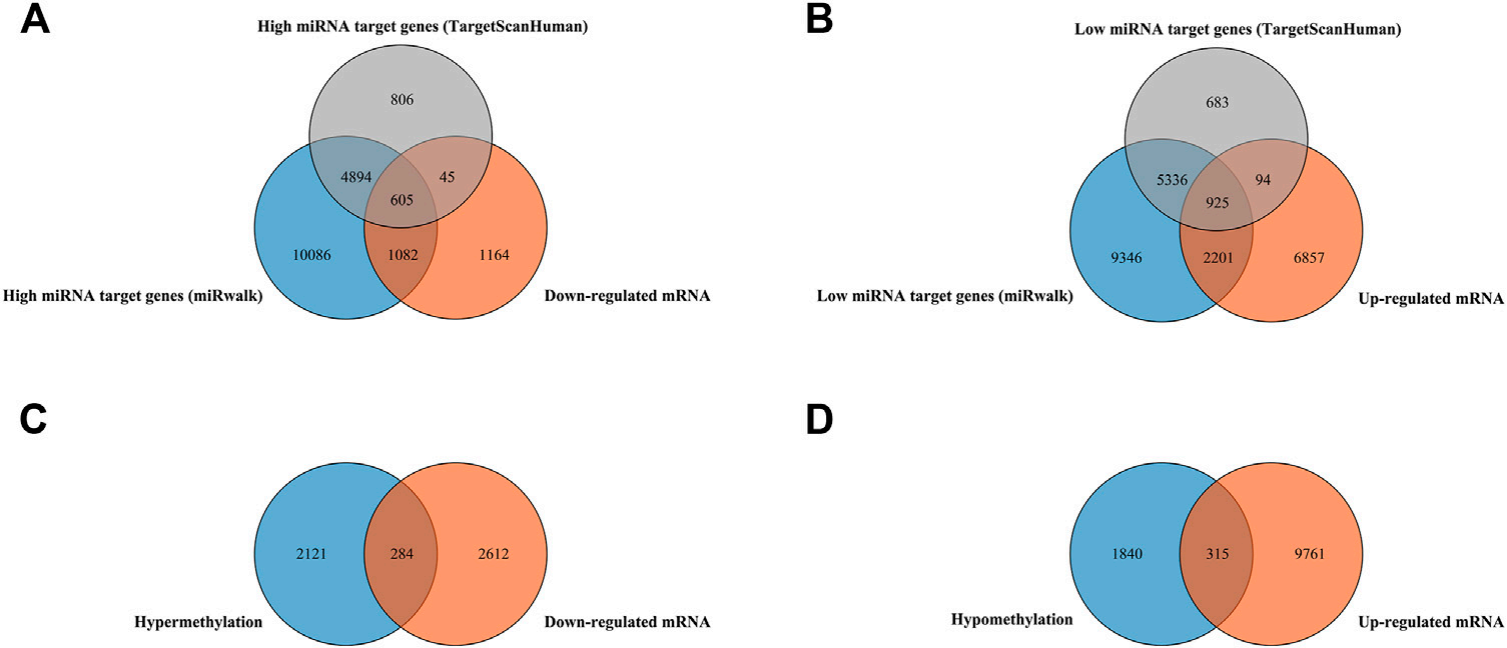
Identification of target genes of differentially expressed miRNAs and mRNA, as well as aberrantly methylated differentially expressed genes between cancer and adjacent samples from LUAD patients. **(A,B)** Target genes of differentially expressed miRNAs and mRNAs; miRNA target genes were predicted by microT-CDS online software and the miRWalk database, respectively. **(C,D)** Aberrantly methylated differentially expressed genes.

Finally, 607 high miRNA-targeting downregulated genes and 925 low miRNA-targeting upregulated genes were screened *via* overlapping target genes of DEMs and DEGs (Figures 1A,B). In addition, 284 hypermethylation-low-expression genes and 315 hypomethylation-high-expression genes by overlapping abnormal methylation and regulated genes were identified (Figures 1C,D).

**FIGURE 2:**
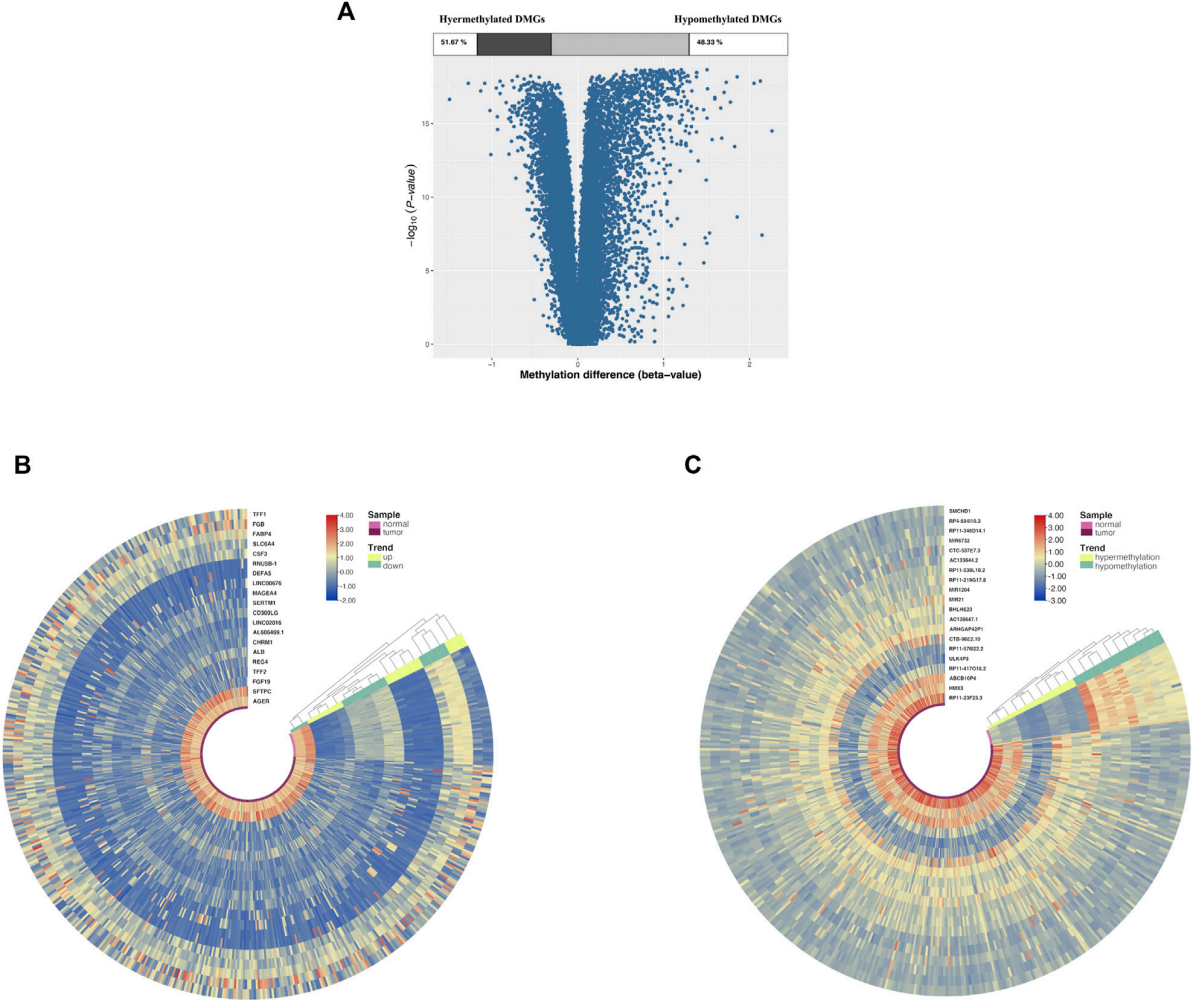
DMG and DEG analysis in cancer and adjacent samples from LUAD patients. **(A)** Volcano plot of DMGs; the percentages of hypermethylated and hypomethylated DMGs are displayed on top. **(B)** Heatmap of top 20 DEGs (10 upregulated genes and 10 downregulated genes). **(C)** Heatmap of top 20 DMGs (10 hypermethylated genes and 10 hypomethylated genes); red indicates that the expression of genes is relatively upregulated, or the level of methylation is hypermethylated, and blue indicated that the expression of genes is relatively downregulated, or the level of methylation is hypomethylated.

Of all DMGs, 51.76% were hypermethylated, and 48.33% were hypomethylated (Figure 2A). Moreover, Figures 2B,C suggested that the DEGs and DMGs (top 20 upregulated and top 20 downregulated genes, as well as top 20 hypermethylation and top 20 hypomethylation genes) can be differentiated between LUAD and normal samples.

### 3.2. DEGs Associated With Altered Targeting miRNAs

#### 3.2.1. Low-Expression miRNAs and Upregulated Genes

**FIGURE 3:**
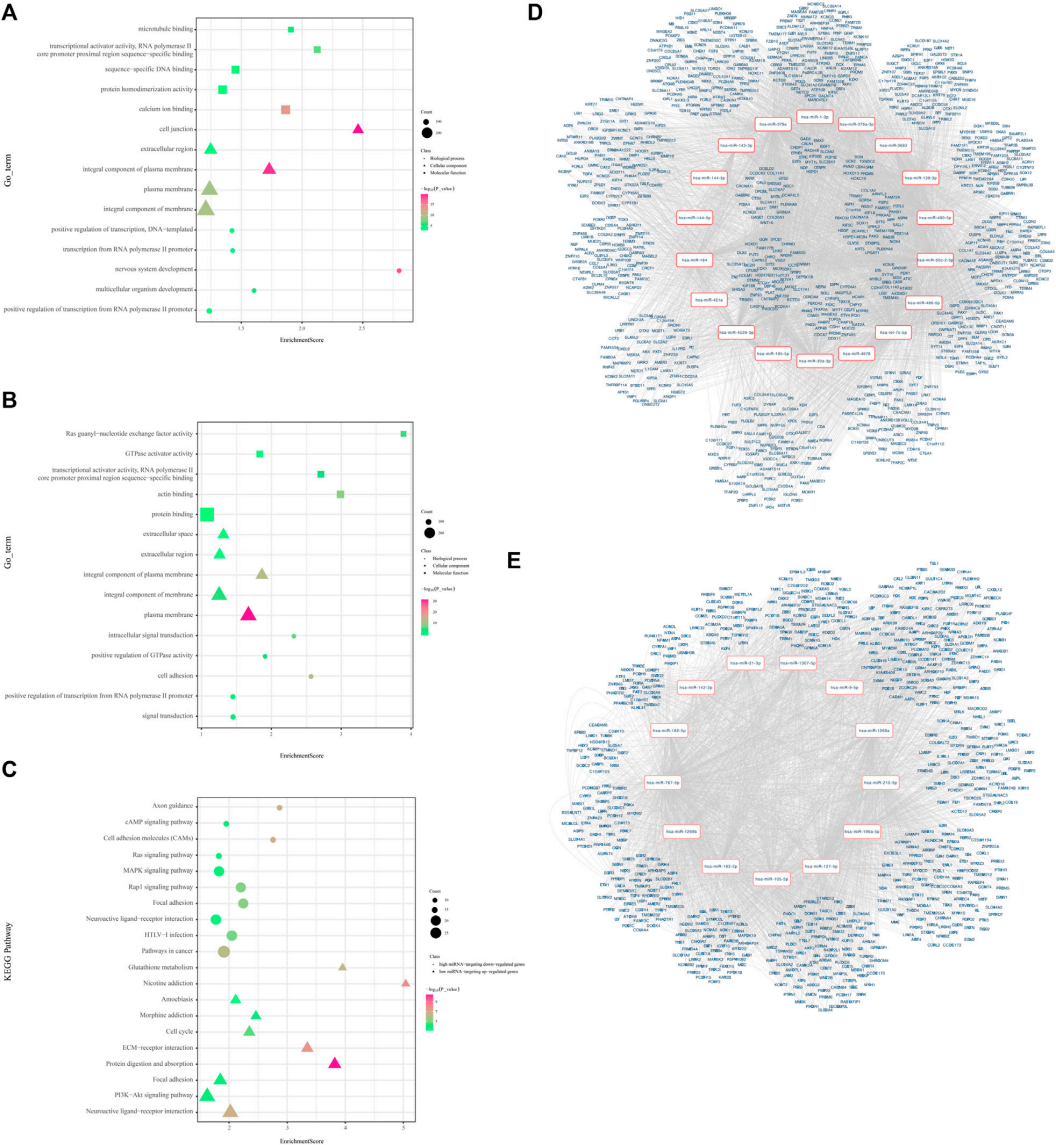
Visualized regulatory network and enrichment bubble graph for miRNA-targeting DEGs. **(A)** Enrichment bubble graph for upregulated genes. **(B)** Enrichment bubble graph for downregulated genes. **(C)** KEGG enrichment bubble chart of low-expression miRNAs and upregulated genes and high-expression miRNAs and downregulated genes. **(D)** Regulatory network graph of 18 low-expression miRNAs. **(E)** Regulatory network graph of 13 high-expression miRNAs.

For low-expression miRNAs and upregulated genes, 168 GO terms were screened with the thresholds of *p* < 0.05, which were mainly associated with the regulation of transcription and cell adhesion (Figure 3A). The most enriched KEGG pathways were the neuroactive ligand–receptor interaction, PI3K-Akt signaling pathway, focal adhesion, protein digestion, and absorption, and ECM–receptor interaction. The KEGG enrichment chart of low-expression miRNAs and upregulated genes is shown in Figures 3C,D. It also shows the miRNA-mRNA network of the 925 upregulated genes.

#### 3.2.2. High-Expression miRNAs and Downregulated Genes

A total of 607 high-expression miRNAs and downregulated genes were enriched in 169 GO terms with the thresholds of *p* < 0.05, which were mainly associated with the regulation of transcription, signal transduction, and cell adhesion (Supplementary Table S3, Figure 3B). The most enriched KEGG pathways were pathways in cancer, HTLV-I infection, neuroactive ligand–receptor interaction, focal adhesion, and the Rap1 signaling pathway. The KEGG enrichment chart of high-expression miRNAs and downregulated genes is shown in Figure 3C. Meanwhile, we constructed the miRNA-mRNA network to reveal further significant miRNA/mRNAs regulated in LUAD progression (Figure 3E).

### 3.3. DEGs Associated With Altered DNA Methylation

#### 3.3.1. Hypermethylation and Low-Expression Genes

**FIGURE 4:**
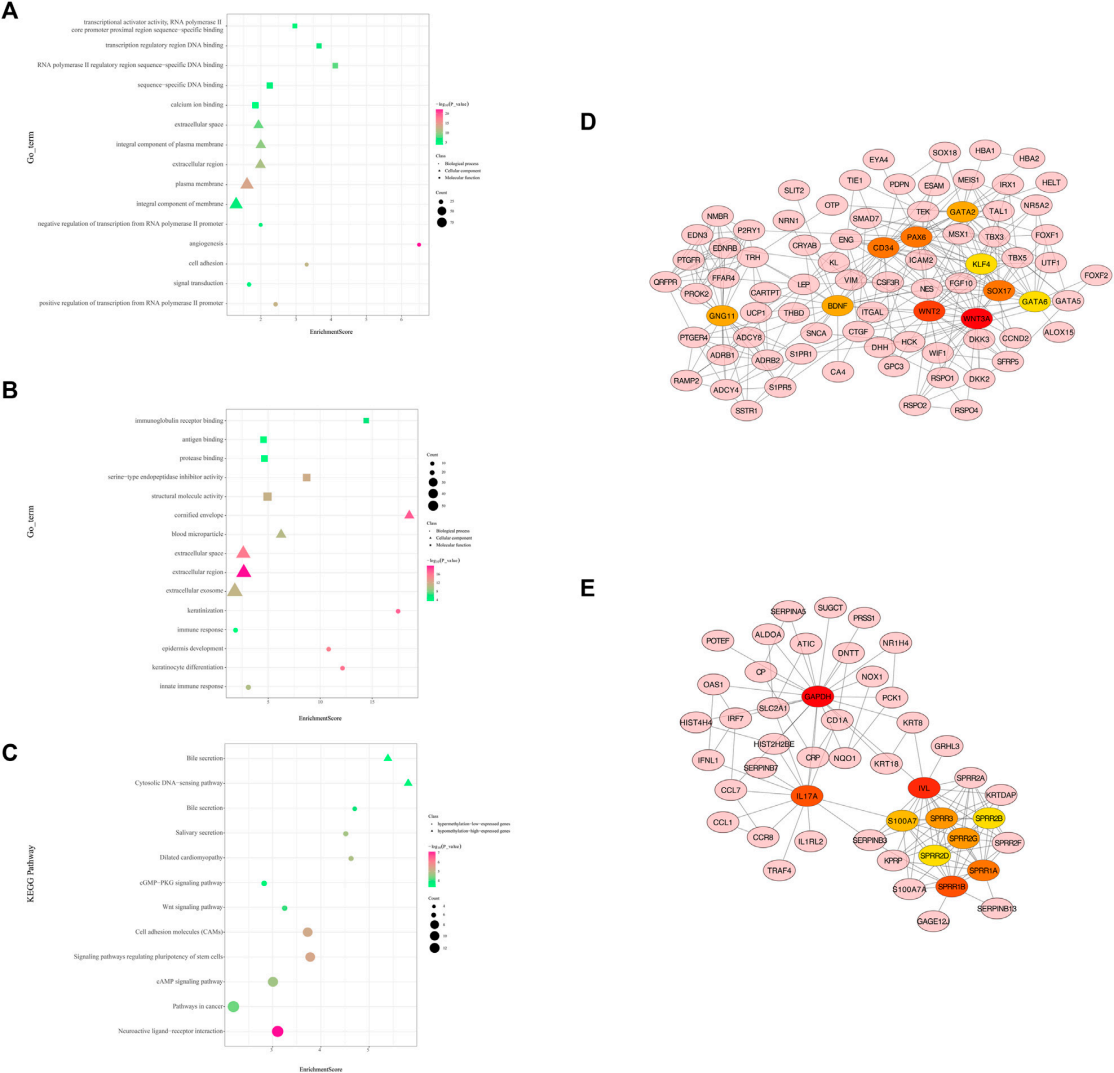
Protein-protein interaction (PPI) network and enrichment bar graph for methylation-related DEGs. **(A)** Enrichment bubble graph for hypermethylated downregulated genes. **(B)** Enrichment bubble graph for hypomethylated upregulated genes. **(C)** KEGG enrichment bubble chart of hypermethylated downregulated genes and hypomethylated upregulated genes. **(D)** PPI network of hypermethylation and low-expression genes between cancer and adjacent samples from LUAD patients (only displays the 10 hub genes and the expanded subnetwork identified by the cytoHubba app in Cytoscape). **(E)** PPI network of hypomethylation and high-expression genes between cancer and adjacent samples from LUAD patients (only displays the 10 hub genes and the expanded subnetwork identified by the cytoHubba app in Cytoscape).

Functional enrichment analysis of hypermethylation and low-expression genes suggested that 141 GO terms were recognized with the thresholds of *p* < 0.05, such as the regulation of transcription, signal transduction, and cell adhesion (Figure 4A). The most enriched KEGG pathways were the neuroactive ligand–receptor interaction, pathways in cancer, cAMP signaling pathway, signaling pathways regulating pluripotency of stem cells, and cell adhesion molecules (CAMs) (Supplementary Table S4, Figure 4C). In total, 191 nodes and 486 edges are shown in the PPI network (Supplementary Figure S1A).

**FIGURE 5:**
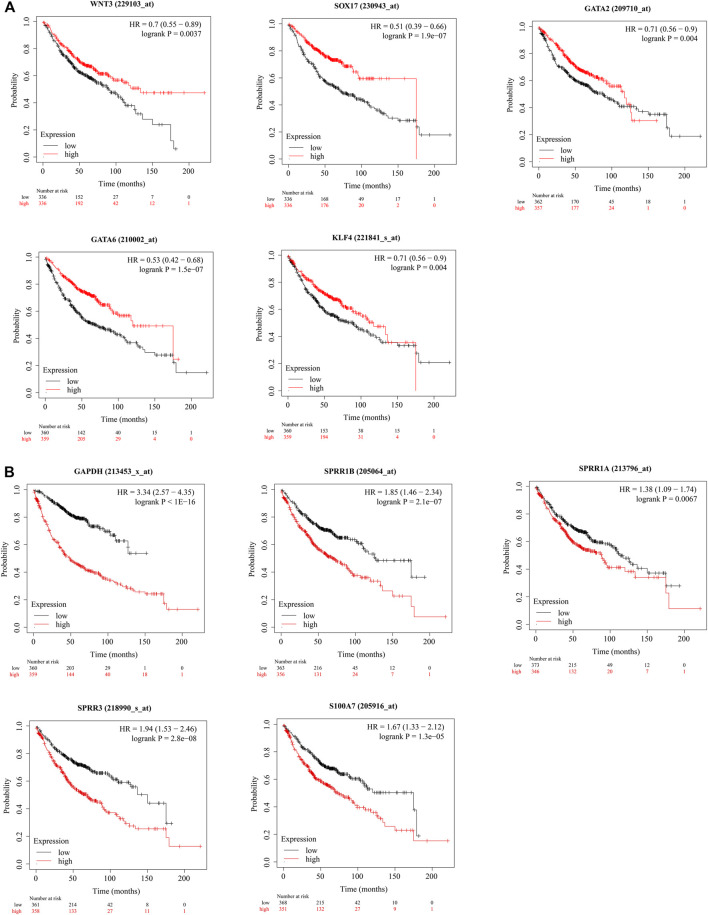
(Continued).

*WNT3A*, *WNT2*, *SOX17*, *CD34*, *PAX6*, *GATA2*, *GNG11*, *BDNF*, *GATA6*, and *KLF4* were identified as hub genes by the degree rank with the cytoHubba app in Cytoscape (Figure 4D, Supplementary Table S5). In these 10 hub genes, *WNAT3A* is calculated with the highest degree (degree = 24). The Kaplan–Meier survival analysis showed that low-expression *WNT3*, *SOX17*, *GATA2*, *GATA6*, and *KLF4* were correlated significantly with poor OS (Figure 5A).

#### 3.3.2. Hypomethylation and High-Expression Genes

As for hypomethylation and high-expression genes, 39 GO terms were identified with the thresholds of *p* < 0.05 (Figure 4B). KEGG pathway analysis recognized enriched cytosolic DNA-sensing pathway and bile secretion (Supplementary Table S4, Figure 4C). In total, 133 nodes and 238 edges were shown in the PPI network (Supplementary Figure S1B).

*GAPDH*, *IVL*, *IL17A*, *SPRR1B*, *SPRR1A*, *SPRR3*, *SPRR2G*, *S100A7*, and *SPRR2A* were identified as hub genes by degree rank with the cytoHubba app (Figure 4E, Supplementary Table S5). GAPDH is calculated with the highest degree (degree = 23). The Kaplan–Meier survival analysis showed that high-expression *GAPDH*, *SPRR1B*, *SPRR1A*, *SPRR3*, and *S100A7* were all significant with poor OS (Figure 5B).

### 3.4. DEGs Associated With Both Abnormal miRNA and DNA Methylation

**FIGURE 6:**
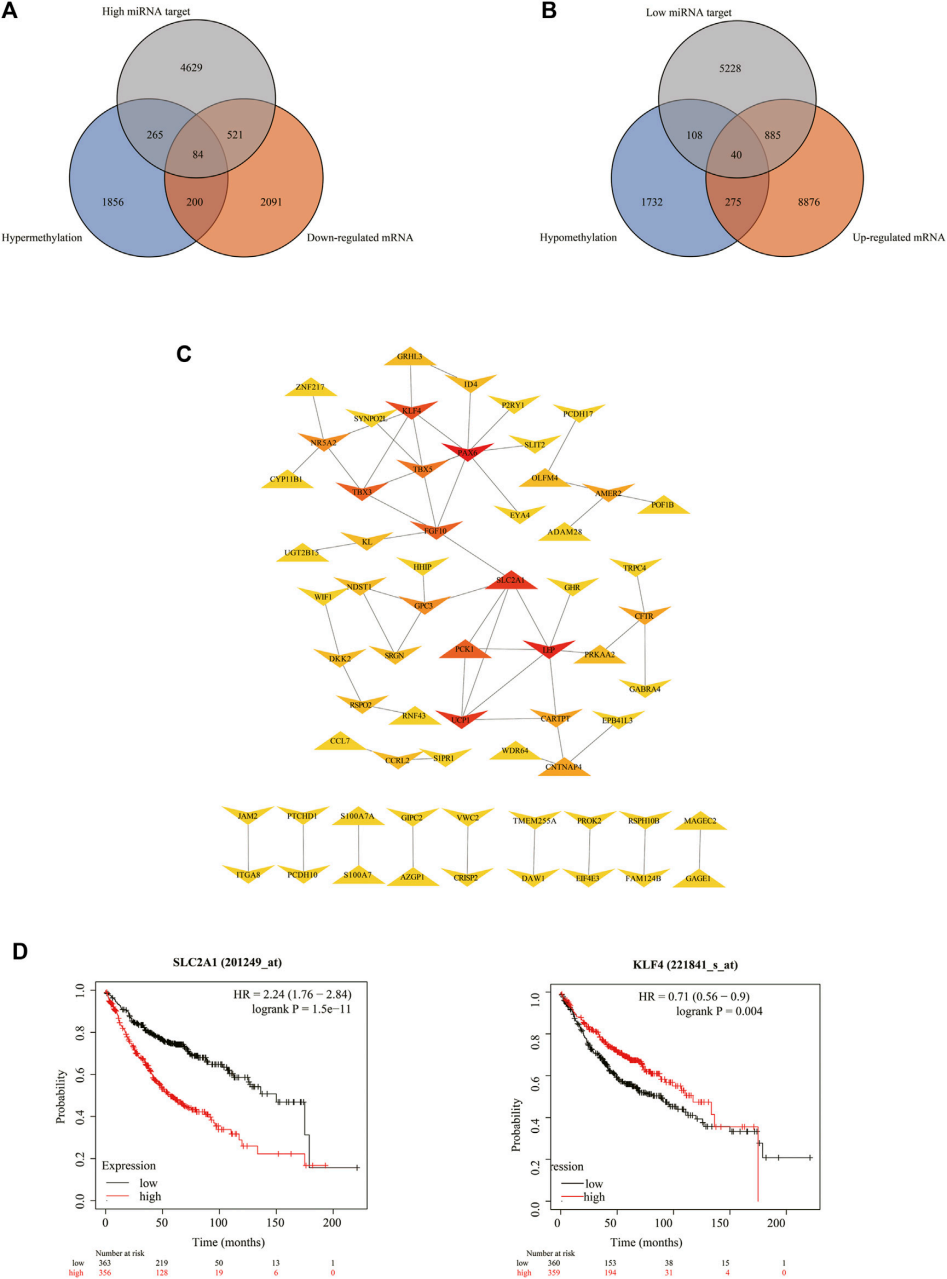
Details for all the overlapped genes. **(A,B)** Venn graph for all the overlapped genes including 84 downregulated genes and 40 upregulated genes, respectively. **(C)** PPI network of all the overlapped genes including 84 downregulated genes and 40 upregulated genes. **(D)** Kaplan–Meier analysis of patients with low (black curve) and high (red curve) expressions of *SLC2A1* and *KLF4*.

**FIGURE 7:**
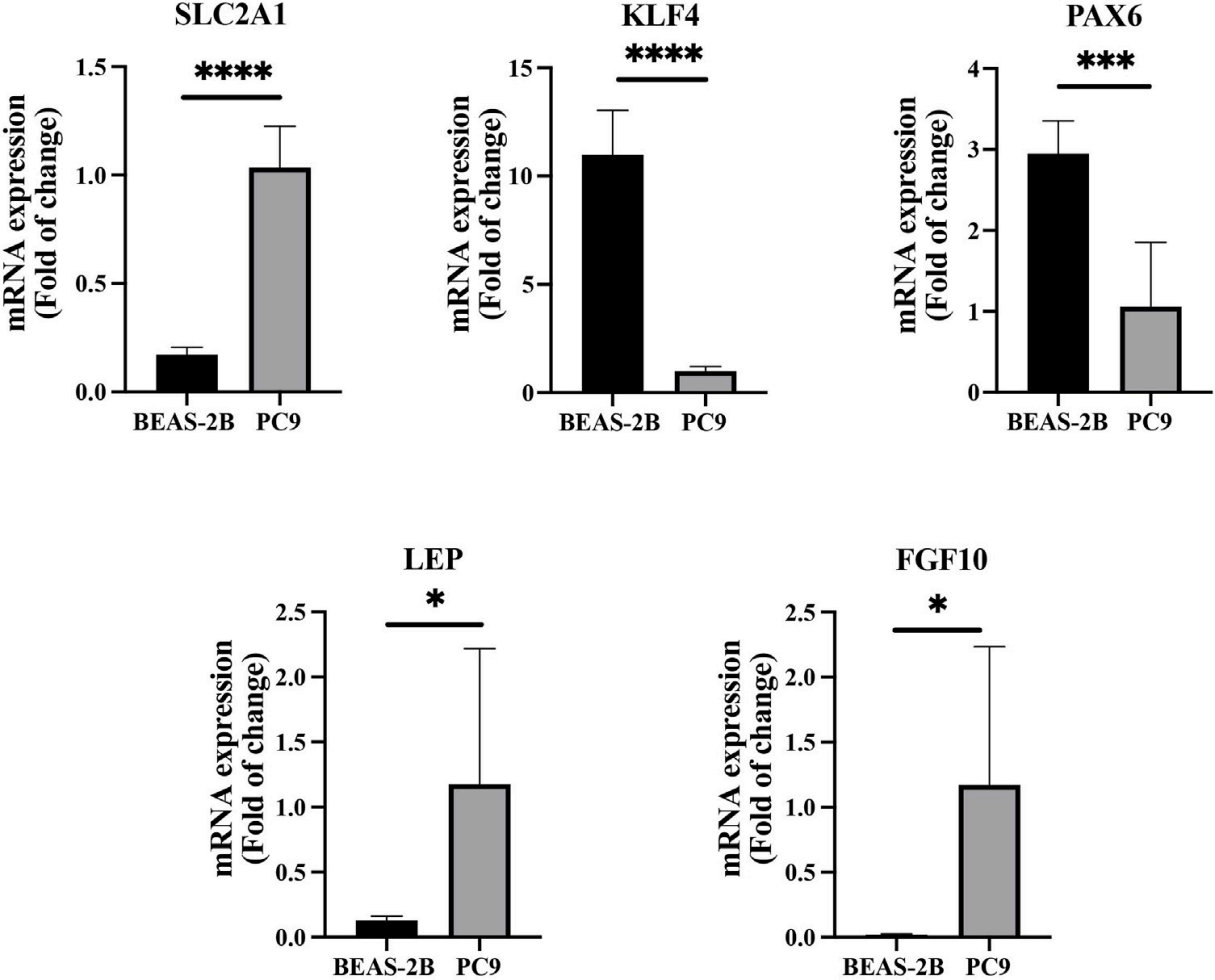
Comparison of the expressions of the five hub genes between the PC9 cell line and BEAS-2B cell lines.

**FIGURE 8:**
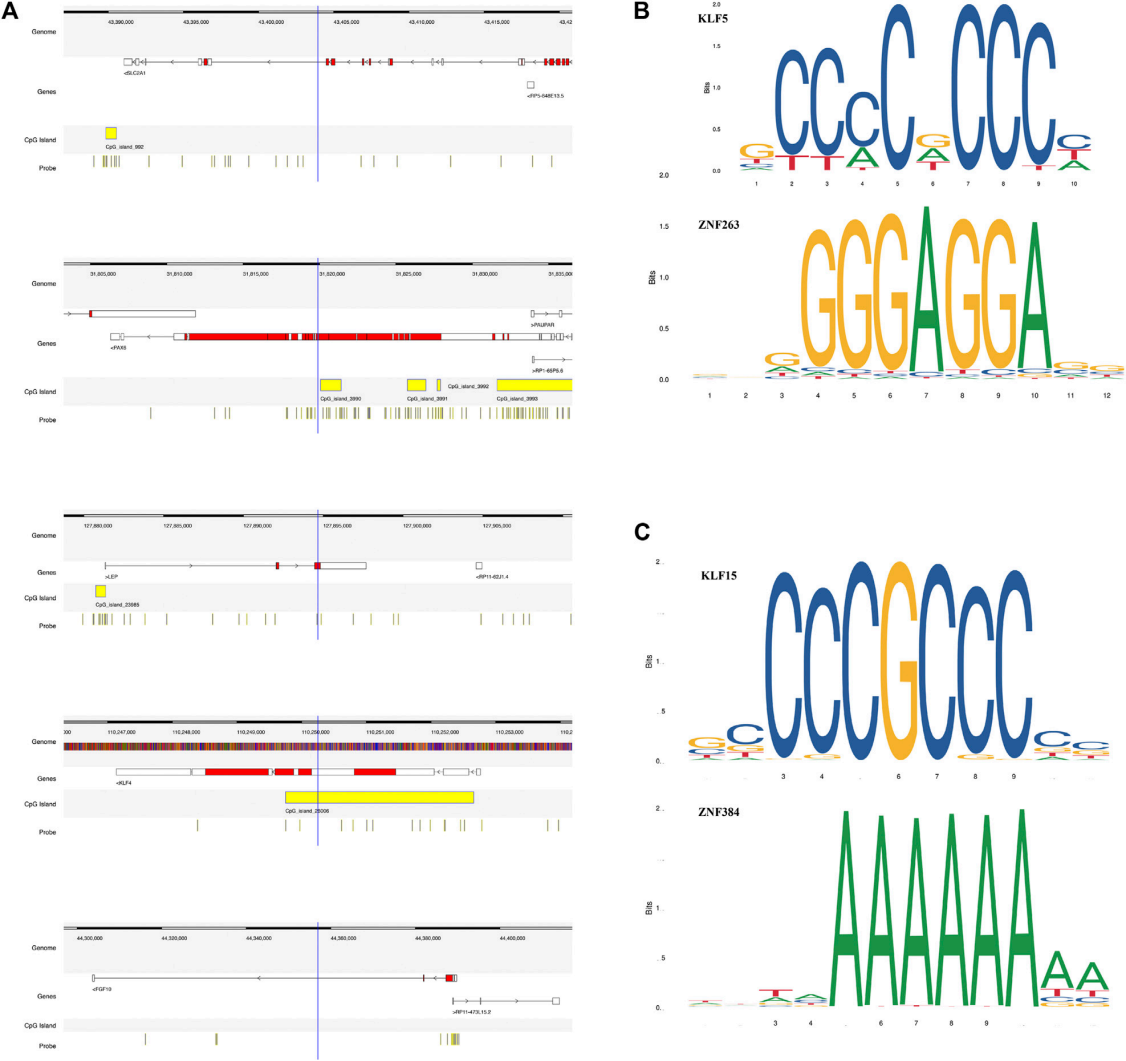
Details of the five screened genes. **(A)** Epigenetic regulatory patterns of both miRNA and DNA methylation-regulating gene expression, as well as CpG island prediction analysis of five screened genes. **(B)** The JASPER database predicted the sequence of two possible transcription factors of *SLC2A1*. **(C)** The JASPER database predicted the sequence of two possible transcription factors of *KLF4*.

We found that several DEGs were regulated by both abnormal miRNA and DNA methylation. It suggested that these DEGs might be of vital importance in the occurrence and development of LUAD. A total of 84 genes such as *FAT4*, *KLF4*, and *EPB41L3* were downregulated under the regulation of both increased miRNA and hypermethylation (Figure 6A). Coincidentally, 40 genes such as *SUGCT*, *RNF43*, and *UGT2B15* were upregulated under the regulation of both decreased miRNA and hypomethylation (Figure 6B). The modulatory miRNA and binding sites, as well as the DNA methylation site, cg ID, and its relation to CpG island, are summarized in Supplementary Table S6. In total, 86 genes, including 29 hypomethylation miRNA-targeting upregulated genes and 57 hypermethylation miRNA-targeting downregulated genes (11 hypomethylated miRNA-targeting upregulated genes and 26 hypermethylated miRNA-targeting downregulated genes without corresponding Affymetrix Probe Set ID on GPL96 cannot be used for CMap), were submitted to the CMap online tool to predict potential drugs in the therapy for LUAD depending on the expression alteration. By ranking the *p*-value in the ascending order and filtering the mean range from −0.5 to 0.5, five small-molecule chemicals were identified as latent treatment options for LUAD (Table 1). Furthermore, a PPI network for all the abnormal expressed genes, including 40 upregulated genes and 84 downregulated (63 nodes and 61 edges) genes were constructed (Figure 6C). Five hub genes were identified for further analysis, including *SLC2A1* with up-regulated expression levels under both low miRNA and hypomethylation regulation, as well as four genes with downregulated expression levels under both high miRNA and hypermethylation regulation of *PAX6*, *LEP*, *KLF4*, and *FGF10*. The Kaplan–Meier survival analysis showed that high-expression *SLC2A1* and low-expression *KLF4* were all significant with poor OS (Figure 6D). To verify the difference in the expression of the five hub genes in TCGA database, we used qRT-PCR to evaluate the expression of the five hub genes at the transcription level and found that the expression levels of *SLC2A1* mRNA (*p* < 0.0001, Figure 7), *LEP* mRNA (*p* < 0.05, Figure 7), and *FGF4* mRNA ((*p* < 0.05, Figure 7) in the PC9 cell line were significantly higher than those in the BEAS-2B cell line. The expression levels of *KLF4* mRNA (*p* < 0.0001, Figure 7) and *PAX6* mRNA (*p* < 0.001, Figure 7) in the PC9 cell line were significantly lower than those in the BEAS-2B cell line. A CpG island prediction has been proceeded, and the results are shown in Figure 8A. The JASPER database predicted the sequence of four possible transcription factors of *SLC2A1* and *KLF4*, as shown in Figures 8B,C. However, further clinical trials are required to verify these findings.

**TABLE1:** Five chemicals were predicted as putative therapeutic agents for LUAD.

CMap name	Chemical formula	Mean	n	Enrichment	*P*
Mecamylamine	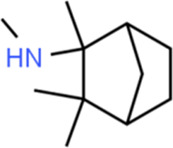	−0.817	3	−0.988	0
LM-1685	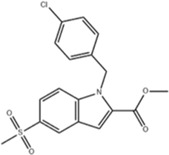	−0.613	3	−0.909	0.00132
5182598	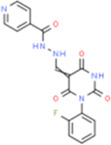	−0.722	2	−0.963	0.00308
Tetracycline	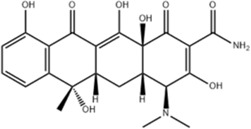	0.578	5	0.733	0.0031
Aminoglutethimide	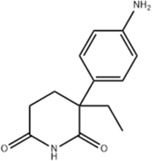	0.556	3	0.853	0.00605

## 4 Discussion

CpG island-specific methylation in the promoter region of genes is associated with gene silencing (Morgan et al., 2018), which changes the expression of downstream hub genes and promotes abnormal cell proliferation (Kulis and Esteller, 2010). DNA methylation and miRNA expression can make a real difference in LUAD by up- or downregulating gene expressions (Herbst et al., 2018; He et al., 2021). Aberrant DNA methylation and miRNA expression can be regarded as impactful biomarkers to distinguish LUAD from normal samples (Ren et al., 2019; Sherafatian and Arjmand, 2019), which would be helpful in diagnosis, assessment of treatment, and prediction of prognosis (Ye et al., 2021). In this present study, data on DNA methylation microarrays, miRNA expression microarrays, and mRNA expression profiling microarrays (the aforementioned data are all obtained from TCGA database) were methodically analyzed, which compare the differential profiling between cancer and adjacent samples from LUAD patients. Hub genes and core pathways have been enriched to screen pivotal events in epigenetic alteration regulated by DNA methylation and miRNA.

A total of 607 high miRNA-targeted downregulated genes were identified through overlapping targets of DEMs and DEGs. The GO analysis showed that these 607 genes are primarily enriched in the cellular component in LUAD, reminding us of the potential regulation of membrane-related metabolism in LUAD. Furthermore, for molecular function, these genes were significantly enriched in protein binding, which indicated an interaction of any protein or protein complex in LUAD. As for KEGG pathway analysis, the target genes were most enriched in pathways in cancer, which suggested that these genes may participate in the tumorigenesis in LUAD. Previous research has indicated that hsa-miR-1269a had the most target genes among the 13 high-expression miRNAs, including *NEGR1*, *ITGA8*, *CLDN18*, *JAM2*, and *JAM3*, associated with cell adhesion. Cell adhesion molecules are a type of membrane surface glycoprotein molecules involved in regulating inflammatory response and promoting the metastasis of LUAD (Liu et al., 2021). Loss of cell adhesion is one of the characteristics of epithelial-to-mesenchymal transition (EMT), and the low expression of adhesion molecules is associated with distant metastasis in LUAD (Kim et al., 2013).

A total of 925 low miRNA targeted upregulated genes by overlapping targets of DEMs and DEGs were finally exhibited. The GO term analysis indicated that the upregulated genes were primarily enriched in integral components of membrane, positive regulation of transcription from RNA polymerase II promoter, and calcium ion binding, which indicated a regulatory role in RNA translation and transcription. The previous study has shown that the activation of Ca2+ in cells may be related to tumorigenicity and metastasis in LUAD (Li et al., 2018). KEGG analysis revealed pathways including the neuroactive ligand–receptor interaction, PI3K-Akt signaling pathway, focal adhesion, and protein digestion and absorption. GABA receptors are regulated by neuroactive steroids and are considered to control cell proliferation (Watanabe et al., 2006). A PI3K-Akt signaling pathway is a key signal medium that activates EMT-induced transcription factors (Karimi Roshan et al., 2019). According to the research, hsa-let-7c-5p upregulated 236 genes, including *COL1A1*, *COL24A1*, *LAMA1*, *ITGA2*, and other genes, and mainly enriched in focal adhesion is associated with EMT (Wu et al., 2021).

Until now, 284 hypermethylation and low expression genes were obtained *via* overlapping strategies of DMGs and DEGs. KEGG pathway analysis showed that hypermethylation-induced disorder of Neuroactive ligand-receptor interaction and Pathways in cancer might cause LUAD. The PPI network of hypermethylation and low-expression genes shows their functional connections; not only the top 10 hub genes among them but also five genes related to prognosis, such as *WNT3*, *SOX17*, *GATA2*, *GATA6*, and *KLF4*, were also selected. Interestingly, eight out of the top 10 hub genes were enriched in the biological process of positive regulation of transcription from the RNA polymerase II promoter.

As for 315 low-methylation and high-expression genes, overlapping hypomethylation and upregulation in LUAD, GO, and KEGG pathway analysis showed enrichment in the innate immune response and cytosolic DNA-sensing pathway. A series of studies indicated that the cytosolic DNA-sensing pathway was associated with antitumor immunity (Amouzegar et al., 2021; Verrier and Langevin, 2021). Therefore, hypomethylation-induced aberrance of high-expression genes may affect the antitumor immunity and promote the progression of LUAD. *GAPDH*, *SPRR1B*, *SPRR1A*, *SPRR3*, and *S100A7* associated with the prognosis of LUAD are obtained through the PPI network. GAPDH is indeed the internal reference used in PCR and Western blot analysis, but GAPDH has also been shown to be dysregulated in the lung, kidney, breast, stomach, glioma, liver, colorectal, melanoma, prostate, pancreatic, and bladder cancers, and GAPDH is generally upregulated in many types of cancer. GAPDH could be utilized as a reference gene for normalizing lung cell lines, while it was de-regulated in non–small cell lung cancer specimens (Schmidt et al., 2005; Nguewa et al., 2008). The de-regulation of GAPDH in tumor tissues or cells demonstrated that the utilization of GAPDH as a reference gene/protein should be chosen very carefully (Guo et al., 2013). In normal tissues, small proline-rich proteins (SPRRs) are involved in the structural integrity of the cornified cell envelope (Patel et al., 2003), and the upregulation of SPRRs is also common under various pathophysiological conditions. Compelling evidence shows that SPRR downregulates p53 and promotes EMT (Demetris et al., 2008; Mizuguchi et al., 2012). A large number of studies have confirmed that SPRR is related to the progression of a variety of tumors (Carregaro et al., 2013). SPRR1 B activates the MAPK signaling pathway involved in LUAD proliferation and metastasis (Zhang Z. et al., 2021), but the influence of other genes of the SPRR family on the progression of LUAD is poorly understood, and further experimental elucidation is needed.

Interestingly, the abnormal expression of DEGs may be regulated by combining the epigenetic alterations of DNA methylation and miRNA. Forty genes such as *SUGCT*, *RNF43*, and *UGT2B15* were upregulated due to the regulation of both decreased DNA methylation and miRNAs, while under the modulation of both increased DNA methylation and miRNAs, 84 genes including *FAT4*, *KLF4*, and *EPB41L3* were downregulated. GO analysis for 40 low miRNA-targeting high-expression hypomethylation genes identified enrichment in cell adhesion, glucose homeostasis, and cellular response to interleukin-1. The three upregulated genes (*CCL7*, *ADAMTS12*, and *PCK1*) are involved in the cellular response to tumor necrosis factor. Moreover, the aforementioned genes are also involved in the glucagon signaling pathway, insulin resistance, bile secretion, and adipocytokine signaling pathway. For 84 high miRNA-targeting and low-expression hypermethylation genes, GO analysis screened the most significantly enriched CC, BP, and MF are integral components of membrane, positive regulation of transcription from the RNA polymerase II promoter, and sequence-specific DNA binding, respectively. Two significant results, neuroactive ligand–receptor interaction and signaling pathways regulating pluripotency of stem cells, were retrieved from the KEGG pathway analysis. Three of the five hub genes (*PAX6*, *LEP*, and *KLF4*) are involved in the aforementioned two pathways; the low expression of KLF4 is associated with poor prognosis. In addition, qRT-PCR was performed to verify the differential expressions of the five hub genes in LUAD. The mRNA expression level of SLC2A1 in the PC9 cell line was observed to be significantly higher than that in the BEAS-2B cell line, and the expression level of mRNA of KLF4 in the PC9 cell line was significantly lower than that in the BEAS-2B cell line. The results of qRT-PCR are consistent with those of bioinformatics analysis, meaning that SLC2A1 may be an oncogene in LUAD, while KLF4 may be a tumor suppressor gene.

Although chemotherapy, targeted therapy, and immunotherapy have brought hope to LUAD patients, drug resistance is still inevitable. It is urgent to find new therapeutic targets, explore new drugs, or reuse existing drugs; online databases can help us predict drugs. By far, the effectiveness of the CMap database has been confirmed by a large number of studies due to its practical value in drug prediction (Aramadhaka et al., 2013; Wang et al., 2016). From the CMap database, five compounds, including mecamylamine, LM-1685, 5182598, tetracycline, and aminoglutethimide, may have significant therapeutic effects on LUAD. Mecamylamine is a nicotinic acetylcholine receptor (nAChR) antagonist; research by Zhu et al. (2003) showed that mecamylamine could reverse the increase in VEGF and circulating endothelial progenitor cells (EPC) caused by secondhand smoke, thereby inhibiting tumor growth and angiogenesis. LM-1685 is a kind of selective COX-2 inhibitor, which induces cancer cell apoptosis and cell cycle arrest and inhibits tumor angiogenesis (Wu et al., 2004; Grosch et al., 2006; Liggett et al., 2014). It was reported that the selective COX-2 inhibitor might enhance the effect of conventional antitumor treatments by intensifying the sensitivity of lung cancer cells to NK cell-mediated cytotoxicity (Kim et al., 2020). It is observed that 5182598 has been reported to be an effective anti-tumor drug from the group of benzylisoquinoline alkaloids (Cordell et al., 2001).

Our research still has some shortcomings. The drug prediction results from the CMap database require a large number of rigorous clinical trials to corroborate their availability in the treatment of LUAD. In addition, the effects of both abnormal DNA methylation and miRNA expression on gene expression also need to be verified by corresponding experiments.

This study indicated that a cavalcade of abnormal methylated differentially expressed genes is related to the epigenetic changes of DNA methylation and miRNAs in LUAD. In total, 607 high miRNA-targeting downregulated genes and 925 low miRNA-targeting upregulated genes were identified by overlapping targets of DEMs and DEGs, which were enriched in the pathways in cancer and the PI3K-Akt signaling pathway, respectively. Furthermore, 284 hypermethylated downregulated genes and 315 hypomethylated upregulated genes obtained by overlapping DMGs and DEGs were associated with the neuroactive ligand–receptor interaction and cytosolic DNA-sensing pathway. Interestingly, 40 genes were upregulated under the co-regulation of hypomethylation and decreased miRNA, while 84 were downregulated under the co-regulation of hypermethylation and increased miRNA. Five small-molecule drugs were identified as potential therapeutic agents for LUAD. Finally, from these genes, *SLC2A1*, *PAX6*, *LEP*, *KLF4*, and *FGF10* were identified as hub genes, especially *SLC2A1* and *KLF4*, which were related to the prognosis of LUAD, and might be used as biomarkers for the precise diagnosis and treatment of LUAD.

## Data Availability

Publicly available datasets were analyzed in this study. This data can be found at: TCGA.
