# Supplementary material for: Epigenetic Alterations of DNA Methylation and miRNA Contribution to Lung Adenocarcinoma
Source: Front Genet. 2022 May 31;13:817552. doi: 10.3389/fgene.2022.817552 (PMC9194831; doi:10.3389/fgene.2022.817552)
Supplement: Supplementary file 3 [file Table1.DOCX]

Supplementary Table 1. Data used in finally quantitative synthesis and integrated analysis.

| **Expression type** | **Data Category** | **Data Type** | **Workflow Type** | **Platform** | Samples(N : P)a |
| --- | --- | --- | --- | --- | --- |
| Methylation profiling | DNA methylation | Methylation Beta Value | Liftover | illumina human methylation 450 | 29:437 |
| mRNA expression | transcriptome profiling | Gene Expressoin Quantification | HTSeq-Counts | NA | 54:497 |
| miRNA expression | transcriptome profiling | miRNA Expression Quantification | miRNA-Seq | NA | 45:483 |

Supplementary Table 2. The detailed characteristics of the top 5 differentially expressed miRNAs.

|  | **ID*** | **sequence** |
| --- | --- | --- |
| ***Up-regulated*** | | |
| hsa-miR-105-5p | MIMAT0000102 | UCAAAUGCUCAGACUCCUGUGGU |
| hsa-miR-1269b | MIMAT0019059 | CUGGACUGAGCCAUGCUACUGG |
| hsa-miR-196a-5p | MIMAT0000226 | UAGGUAGUUUCAUGUUGUUGGG |
| hsa-miR-767-5p | MIMAT0003882 | UGCACCAUGGUUGUCUGAGCAUG |
| hsa-miR-9-5p | MIMAT0000441 | UCUUUGGUUAUCUAGCUGUAUGA |
| ***Down-regulated*** | | |
| hsa-miR-378e | MIMAT0018927 | ACUGGACUUGGAGUCAGGA |
| hsa-miR-3683 | MIMAT0018111 | UGCGACAUUGGAAGUAGUAUCA |
| hsa-miR-486-5p | MIMAT0002177 | UCCUGUACUGAGCUGCCCCGAG |
| hsa-miR-139-3p | MIMAT0004552 | UGGAGACGCGGCCCUGUUGGAGU |
| hsa-miR-4529-3p | MIMAT0019068 | AUUGGACUGCUGAUGGCCCGU |

**miRBase ID*

Supplementary Table 3. GO and KEGG pathway analysis of DEGs targeted by altered miRNAs between cancer and adjacent samples from LUAD patients.

| **Category** | **Term** | **Count** | **Precent (%)** | **P value** |
| --- | --- | --- | --- | --- |
| ***High miRNA targeting down-regulated genes*** | | | | |
| Biological Process | signal transduction | 57 | 9.39 | 0.00 |
| Biological Process | positive regulation of transcription from RNA polymerase II promoter | 48 | 7.91 | 0.00 |
| Biological Process | cell adhesion | 39 | 6.43 | 0.00 |
| Biological Process | positive regulation of GTPase activity | 36 | 5.93 | 0.00 |
| Biological Process | intracellular signal transduction | 31 | 5.11 | 0.00 |
| Cellular Component | plasma membrane | 214 | 35.26 | 0.00 |
| Cellular Component | integral component of membrane | 201 | 33.11 | 0.00 |
| Cellular Component | integral component of plasma membrane | 82 | 13.51 | 0.00 |
| Cellular Component | extracellular region | 63 | 10.38 | 0.04 |
| Cellular Component | extracellular space | 55 | 9.06 | 0.03 |
| Molecular Function | protein binding | 297 | 48.93 | 0.03 |
| Molecular Function | actin binding | 26 | 4.28 | 0.00 |
| Molecular Function | transcriptional activator activity, RNA polymerase II core promoter proximal region sequence-specific binding | 20 | 3.29 | 0.00 |
| Molecular Function | GTPase activator activity | 16 | 2.64 | 0.03 |
| Molecular Function | Ras guanyl-nucleotide exchange factor activity | 14 | 2.31 | 0.00 |
| KEGG Pathway | Pathways in cancer | 26 | 4.28 | 0.00 |
| KEGG Pathway | HTLV-I infection | 18 | 2.97 | 0.01 |
| KEGG Pathway | Neuroactive ligand-receptor interaction | 17 | 2.80 | 0.03 |
| KEGG Pathway | Focal adhesion | 16 | 2.64 | 0.00 |
| KEGG Pathway | Rap1 signaling pathway | 16 | 2.64 | 0.01 |
| ***Low miRNA targeting up-regulated genes*** | | | | |
| Biological Process | positive regulation of transcription from RNA polymerase II promoter | 60 | 6.49 | 0.04 |
| Biological Process | multicellular organism development | 41 | 4.43 | 0.00 |
| Biological Process | nervous system development | 39 | 4.22 | 0.00 |
| Biological Process | transcription from RNA polymerase II promoter | 36 | 3.89 | 0.02 |
| Biological Process | positive regulation of transcription, DNA-templated | 36 | 3.89 | 0.02 |
| Cellular Component | integral component of membrane | 298 | 32.22 | 0.00 |
| Cellular Component | plasma membrane | 244 | 26.38 | 0.00 |
| Cellular Component | integral component of plasma membrane | 117 | 12.65 | 0.00 |
| Cellular Component | extracellular region | 96 | 10.38 | 0.02 |
| Cellular Component | cell junction | 54 | 5.84 | 0.00 |
| Molecular Function | calcium ion binding | 64 | 6.92 | 0.00 |
| Molecular Function | protein homodimerization activity | 47 | 5.08 | 0.03 |
| Molecular Function | sequence-specific DNA binding | 36 | 3.89 | 0.03 |
| Molecular Function | transcriptional activator activity, RNA polymerase II core promoter proximal region sequence-specific binding | 24 | 2.59 | 0.00 |
| Molecular Function | microtubule binding | 19 | 2.05 | 0.01 |
| KEGG Pathway | Neuroactive ligand-receptor interaction | 25 | 2.70 | 0.00 |
| KEGG Pathway | PI3K-Akt signaling pathway | 25 | 2.70 | 0.02 |
| KEGG Pathway | Focal adhesion | 17 | 1.84 | 0.02 |
| KEGG Pathway | Protein digestion and absorption | 15 | 1.62 | 0.00 |
| KEGG Pathway | ECM-receptor interaction | 13 | 1.41 | 0.00 |

Supplementary Table 4. Gene ontology and KEGG pathway analysis of DEGs associated with aberrant DNA methylation between cancer and adjacent samples from LUAD patients.

| **Category** | **Term** | **Count** | **Precent (%)** | ***P*** value |
| --- | --- | --- | --- | --- |
| ***Hypomethylation and up-regulated genes*** | | | | |
| Biological Process | innate immune response | 16 | 5.28 | 0.00 |
| Biological Process | keratinocyte differentiation | 10 | 3.30 | 0.00 |
| Biological Process | epidermis development | 10 | 3.30 | 0.00 |
| Biological Process | immune response | 10 | 3.30 | 0.03 |
| Biological Process | keratinization | 9 | 2.97 | 0.00 |
| Cellular Component | extracellular exosome | 53 | 17.49 | 0.00 |
| Cellular Component | extracellular region | 45 | 14.85 | 0.00 |
| Cellular Component | extracellular space | 37 | 12.21 | 0.00 |
| Cellular Component | blood microparticle | 10 | 3.30 | 0.00 |
| Cellular Component | cornified envelope | 9 | 2.97 | 0.00 |
| Molecular Function | structural molecule activity | 13 | 4.29 | 0.00 |
| Molecular Function | serine-type endopeptidase inhibitor activity | 9 | 2.97 | 0.00 |
| Molecular Function | protease binding | 5 | 1.65 | 0.02 |
| Molecular Function | antigen binding | 5 | 1.65 | 0.02 |
| Molecular Function | immunoglobulin receptor binding | 4 | 1.32 | 0.00 |
| KEGG Pathway | Cytosolic DNA-sensing pathway | 4 | 1.32 | 0.03 |
| KEGG Pathway | Bile secretion | 4 | 1.32 | 0.04 |
| ***Hypermethylation and down-regulated genes*** | | | | |
| Biological Process | positive regulation of transcription from RNA polymerase II promoter | 33 | 2.45 | 0.00 |
| Biological Process | signal transduction | 27 | 1.69 | 0.01 |
| Biological Process | cell adhesion | 21 | 3.33 | 0.00 |
| Biological Process | angiogenesis | 20 | 6.52 | 0.00 |
| Biological Process | negative regulation of transcription from RNA polymerase II promoter | 20 | 2.02 | 0.00 |
| Cellular Component | integral component of membrane | 90 | 1.30 | 0.00 |
| Cellular Component | plasma membrane | 89 | 1.61 | 0.00 |
| Cellular Component | extracellular region | 43 | 1.99 | 0.00 |
| Cellular Component | integral component of plasma membrane | 38 | 2.00 | 0.00 |
| Cellular Component | extracellular space | 35 | 1.93 | 0.00 |
| Molecular Function | calcium ion binding | 17 | 1.84 | 0.02 |
| Molecular Function | sequence-specific DNA binding | 15 | 2.25 | 0.01 |
| Molecular Function | RNA polymerase II regulatory region sequence-specific DNA binding | 11 | 4.11 | 0.00 |
| Molecular Function | transcription regulatory region DNA binding | 10 | 3.65 | 0.00 |
| Molecular Function | transcriptional activator activity, RNA polymerase II core promoter proximal region sequence-specific binding | 9 | 2.97 | 0.01 |
| KEGG Pathway | Neuroactive ligand-receptor interaction | 13 | 4.69 | 0.00 |
| KEGG Pathway | Pathways in cancer | 13 | 4.69 | 0.01 |
| KEGG Pathway | cAMP signaling pathway | 9 | 3.25 | 0.01 |
| KEGG Pathway | Signaling pathways regulating pluripotency of stem cells | 8 | 2.89 | 0.00 |
| KEGG Pathway | Cell adhesion molecules (CAMs) | 8 | 2.89 | 0.01 |

Supplementary Table 5. Hub genes with the top 10 degrees of both high expression and low expression genes.

| **Gene** | **Gene Description** | **Degree** |
| --- | --- | --- |
| ***Hypomethylation and High-Expression hub Genes*** | | |
| GAPDH | glyceraldehyde-3-phosphate dehydrogenase | 23 |
| IVL | involucrin | 16 |
| SPRR1B | small proline rich protein 1B | 14 |
| IL17A | interleukin 17A | 14 |
| SPRR1A | small proline rich protein 1A | 13 |
| SPRR2G | small proline rich protein 2G | 12 |
| SPRR3 | small proline rich protein 3 | 12 |
| S100A7 | S100 calcium binding protein A7 | 11 |
| SPRR2A | small proline rich protein 2A | 9 |
| SPRR2D | small proline rich protein 2D | 9 |
| ***Hypermethylation and Low-Expression hub Genes*** | | |
| WNT3A | Wnt family member 3A | 24 |
| WNT2 | Wnt family member 2 | 20 |
| CD34 | CD34 molecule | 19 |
| PAX6 | paired box 6 | 19 |
| SOX17 | SRY-box transcription factor 17 | 19 |
| GNG11 | G protein subunit gamma 11 | 18 |
| BDNF | brain derived neurotrophic factor | 18 |
| GATA2 | GATA binding protein 2 | 18 |
| GATA6 | GATA binding protein 6 | 17 |
| KLF4 | Kruppel like factor 4 | 17 |

Supplementary Table 6. DEGs associated with both specific miRNA and DNA methylation CpG sites between cancer and adjacent samples from LUAD patients.

| **Gene** | **DNA Methylation** | | | **miRNA** | |
| --- | --- | --- | --- | --- | --- |
|  | **cg ID** | **Site** | **Relation to CpG Island** | **miR** | **Binding site** |
| *Up-regulated genes affected by both low miRNA and hypomethylation* | | | | | |
| SUGCT | cg00420559 | Body | OpenSea | hsa-miR-30a-3p | 3'UTR |
|  | cg00724098 | TSS200 | Island |  |  |
|  | cg01083434 | TSS1500 | Island |  |  |
|  | cg01643856 | Body | S_Shelf |  |  |
|  | cg02426324 | Body | OpenSea |  |  |
|  | cg02976405 | TSS1500 | Island |  |  |
|  | cg03697881 | Body | OpenSea |  |  |
|  | cg04099158 | Body | OpenSea |  |  |
|  | cg05119115 | Body | OpenSea |  |  |
|  | cg05769505 | Body | OpenSea |  |  |
|  | cg05895410 | Body | OpenSea |  |  |
|  | cg08136893 | TSS1500 | Island |  |  |
|  | cg08646805 | Body | OpenSea |  |  |
|  | cg09340267 | TSS200 | Island |  |  |
|  | cg10036840 | Body | OpenSea |  |  |
|  | cg10147982 | Body | OpenSea |  |  |
|  | cg10274696 | Body | OpenSea |  |  |
|  | cg10504632 | Body | OpenSea |  |  |
|  | cg10515180 | Body | OpenSea |  |  |
|  | cg12498409 | TSS200 | Island |  |  |
|  | cg13392320 | Body | OpenSea |  |  |
|  | cg14684441 | 1stExon | Island |  |  |
|  | cg15877233 | Body | OpenSea |  |  |
|  | cg17321668 | TSS1500 | Island |  |  |
|  | cg17389988 | Body | OpenSea |  |  |
|  | cg18175518 | Body | OpenSea |  |  |
|  | cg21326613 | Body | OpenSea |  |  |
|  | cg21653489 | Body | OpenSea |  |  |
|  | cg21983151 | Body | S_Shore |  |  |
|  | cg22284975 | Body | Island |  |  |
|  | cg23249624 | TSS1500 | Island |  |  |
|  | cg23439458 | TSS1500 | Island |  |  |
|  | cg26031288 | Body | OpenSea |  |  |
|  | cg27402881 | TSS1500 | Island |  |  |
| RNF43 | cg04544475 | Body | OpenSea | hsa-miR-30c-2-3p | 3'UTR |
|  | cg04654677 | TSS1500 | OpenSea |  |  |
|  | cg04780629 | TSS200 | OpenSea |  |  |
|  | cg14398214 | 5'UTR | OpenSea |  |  |
|  | cg18366480 | Body | OpenSea |  |  |
|  | cg21917866 | 3'UTR | S_Shore |  |  |
|  | cg24835159 | 5'UTR;1stExon | OpenSea |  |  |
| UGT2B15 | cg20840634 | TSS1500 | OpenSea | hsa-miR-490-5p | CDS |
|  | cg22161115 | 1stExon | OpenSea |  |  |
| GRHL3 | cg01119512 | Body | N_Shore | hsa-miR-139-3p | CDS |
|  | cg04552500 | 1stExon;5'UTR | N_Shelf |  |  |
|  | cg05036846 | TSS1500;Body | Island |  |  |
|  | cg06376426 | TSS1500;Body | Island |  |  |
|  | cg09662754 | TSS200 | N_Shelf |  |  |
|  | cg11155431 | Body | OpenSea |  |  |
|  | cg12405265 | Body;5'UTR | OpenSea |  |  |
|  | cg12757705 | TSS200 | N_Shelf |  |  |
|  | cg13976753 | 3'UTR | OpenSea |  |  |
|  | cg13987674 | TSS200;Body | S_Shore |  |  |
|  | cg14167629 | 1stExon | N_Shelf |  |  |
|  | cg14616251 | TSS1500;Body | Island |  |  |
|  | cg15050111 | TSS200 | N_Shelf |  |  |
|  | cg16155382 | Body | N_Shelf |  |  |
|  | cg18196063 | Body;5'UTR | S_Shelf |  |  |
|  | cg19697558 | Body | N_Shelf |  |  |
|  | cg20242797 | TSS200 | N_Shelf |  |  |
|  | cg20325200 | Body | OpenSea |  |  |
|  | cg21273275 | TSS1500;Body | Island |  |  |
|  | cg21289280 | 1stExon;5'UTR | N_Shelf |  |  |
|  | cg22176324 | TSS1500;Body | Island |  |  |
|  | cg23820945 | 1stExon;5'UTR | N_Shelf |  |  |
|  | cg26365938 | 1stExon;5'UTR | N_Shelf |  |  |
| OR51E1 | cg06735218 | TSS1500 | OpenSea | hsa-miR-143-3p | 3'UTR |
|  | cg16604126 | TSS1500 | OpenSea |  |  |
|  | cg18438823 | 3'UTR | OpenSea |  |  |
|  | cg23632744 | Body | OpenSea |  |  |
|  | cg27183113 | TSS200 | OpenSea |  |  |
| HCAR1 | cg00357958 | 5'UTR;1stExon | OpenSea | hsa-miR-378e | 3'UTR |
|  | cg01306688 | TSS200 | OpenSea |  |  |
|  | cg05290737 | TSS200 | OpenSea |  |  |
|  | cg13702536 | TSS1500 | OpenSea |  |  |
|  | cg15823495 | 1stExon | OpenSea |  |  |
|  | cg17346246 | 1stExon | OpenSea |  |  |
|  | cg19328828 | TSS1500 | OpenSea |  |  |
|  | cg19975916 | TSS200 | OpenSea |  |  |
|  | cg20566840 | TSS200 | OpenSea |  |  |
|  | cg22534509 | 5'UTR;1stExon | OpenSea |  |  |
|  | cg22972858 | TSS200 | OpenSea |  |  |
|  | cg23505823 | TSS1500 | OpenSea |  |  |
|  | cg26643476 | TSS1500 | OpenSea |  |  |
| ACP6 | cg00543196 | TSS200 | Island | hsa-let-7c-5p | 3'UTR |
|  | cg01834614 | TSS1500 | S_Shore |  |  |
|  | cg03401580 | TSS200 | Island |  |  |
|  | cg05755010 | TSS1500 | Island |  |  |
|  | cg05968052 | 1stExon;5'UTR | Island |  |  |
|  | cg06988368 | TSS200 | Island |  |  |
|  | cg09066439 | 1stExon;5'UTR | Island |  |  |
|  | cg09264377 | TSS1500 | S_Shore |  |  |
|  | cg12614442 | TSS200 | Island |  |  |
|  | cg15636040 | TSS200 | Island |  |  |
|  | cg16790610 | TSS1500 | Island |  |  |
|  | cg18709479 | 1stExon;5'UTR | Island |  |  |
|  | cg19693476 | TSS200 | Island |  |  |
|  | cg27244432 | 1stExon;5'UTR | Island |  |  |
| PATE4 | cg05435295 | 5'UTR;1stExon | OpenSea | hsa-miR-30c-2-3p | 3'UTR |
|  | cg22329515 | Body | OpenSea |  |  |
|  | cg27618216 | TSS200 | OpenSea |  |  |
| ADAMTS12 | cg01990593 | Body | N_Shore | hsa-let-7c-5p | 3'UTR |
|  | cg04124352 | TSS1500 | S_Shore |  |  |
|  | cg04578894 | Body | OpenSea |  |  |
|  | cg06448603 | Body | OpenSea |  |  |
|  | cg07784793 | Body | OpenSea |  |  |
|  | cg08768395 | Body | N_Shelf |  |  |
|  | cg09747891 | TSS1500 | S_Shore |  |  |
|  | cg10594543 | Body | OpenSea |  |  |
|  | cg10627511 | Body | OpenSea |  |  |
|  | cg12917072 | Body | OpenSea |  |  |
|  | cg18519308 | TSS1500 | S_Shore |  |  |
|  | cg19641747 | Body | OpenSea |  |  |
|  | cg21569398 | Body | OpenSea |  |  |
|  | cg23236370 | TSS1500 | S_Shore |  |  |
|  | cg23359363 | Body | OpenSea |  |  |
|  | cg26573704 | TSS200 | Island |  |  |
| S100A7 | cg00325910 | Body | OpenSea | hsa-miR-30c-2-3p | 3'UTR |
|  | cg02892624 | 5'UTR | OpenSea |  |  |
|  | cg17421062 | 5'UTR | OpenSea |  |  |
| PAEP | cg01055695 | TSS1500 | OpenSea | hsa-miR-490-5p | CDS |
|  | cg03264414 | 1stExon | OpenSea |  |  |
|  | cg14532484 | 3'UTR | OpenSea |  |  |
| SLC2A1 | cg00102166 | TSS1500 | S_Shore | hsa-let-7c-5p | 3'UTR |
|  | cg01907688 | TSS200 | S_Shore |  |  |
|  | cg01924561 | Body | OpenSea |  |  |
|  | cg03106288 | 5'UTR;1stExon | Island |  |  |
|  | cg03128534 | Body | Island |  |  |
|  | cg04287330 | Body | Island |  |  |
|  | cg05034603 | Body | OpenSea |  |  |
|  | cg05802386 | Body | N_Shore |  |  |
|  | cg06094523 | Body | OpenSea |  |  |
|  | cg07499643 | Body | Island |  |  |
|  | cg07803811 | Body | Island |  |  |
|  | cg08159148 | 5'UTR;1stExon | Island |  |  |
|  | cg09502149 | Body | OpenSea |  |  |
|  | cg09824328 | TSS1500 | S_Shore |  |  |
|  | cg12101479 | TSS1500 | S_Shore |  |  |
|  | cg12656391 | TSS1500 | S_Shore |  |  |
|  | cg13790796 | 5'UTR;1stExon | Island |  |  |
|  | cg15089806 | Body | OpenSea |  |  |
|  | cg16738646 | Body | OpenSea |  |  |
|  | cg20282814 | Body | N_Shore |  |  |
|  | cg20294984 | Body | OpenSea |  |  |
|  | cg20345840 | 5'UTR;1stExon | Island |  |  |
|  | cg21474257 | Body | OpenSea |  |  |
|  | cg21877974 | Body | Island |  |  |
|  | cg22025263 | Body | N_Shelf |  |  |
|  | cg22176566 | 5'UTR;1stExon | Island |  |  |
|  | cg26188818 | Body | Island |  |  |
|  | cg26681016 | TSS200 | S_Shore |  |  |
| ADAM28 | cg14976447 | Body | OpenSea | hsa-let-7c-5p | 3'UTR |
|  | cg17969683 | TSS200 | OpenSea |  |  |
|  | cg18757155 | TSS200 | OpenSea |  |  |
|  | cg22915945 | 5'UTR;1stExon | OpenSea |  |  |
| FCRL2 | cg04559323 | TSS1500 | OpenSea | hsa-miR-30a-3p | CDS |
|  | cg05777583 | 3'UTR | OpenSea |  |  |
|  | cg05784088 | Body | OpenSea |  |  |
|  | cg12282588 | Body | OpenSea |  |  |
|  | cg15761405 | Body | OpenSea |  |  |
|  | cg20674248 | Body | OpenSea |  |  |
| PCK1 | cg03840472 | Body | OpenSea | hsa-miR-486-5p | CDS |
|  | cg09282500 | TSS200 | OpenSea |  |  |
|  | cg13655303 | TSS1500 | OpenSea |  |  |
|  | cg13904968 | TSS200 | OpenSea |  |  |
|  | cg16101800 | Body | OpenSea |  |  |
|  | cg16994880 | 3'UTR | OpenSea |  |  |
|  | cg20605413 | 5'UTR;1stExon | OpenSea |  |  |
|  | cg20810198 | TSS200 | OpenSea |  |  |
| ATIC | cg01280080 | Body | S_Shore | hsa-miR-30c-2-3p | 5'UTR |
|  | cg01839430 | Body | Island |  |  |
|  | cg02319613 | TSS1500 | N_Shore |  |  |
|  | cg05691168 | 1stExon;5'UTR | Island |  |  |
|  | cg05734400 | TSS200 | Island |  |  |
|  | cg06417454 | TSS200 | Island |  |  |
|  | cg06525280 | Body | S_Shore |  |  |
|  | cg08803144 | TSS200 | Island |  |  |
|  | cg10535143 | TSS200 | N_Shore |  |  |
|  | cg14933257 | TSS200 | Island |  |  |
|  | cg16935258 | TSS1500 | N_Shore |  |  |
|  | cg21512817 | 1stExon;5'UTR | Island |  |  |
|  | cg24933605 | Body | Island |  |  |
| OLFM4 | cg01098981 | TSS1500 | OpenSea | hsa-miR-30c-2-3p | 3'UTR |
|  | cg02570298 | Body | OpenSea |  |  |
|  | cg08119452 | TSS1500 | OpenSea |  |  |
|  | cg12582008 | Body | OpenSea |  |  |
|  | cg12824060 | TSS200 | OpenSea |  |  |
|  | cg16520357 | 3'UTR | OpenSea |  |  |
|  | cg24932628 | 1stExon | OpenSea |  |  |
| NEFM | cg01583969 | Body;5'UTR | S_Shore | hsa-miR-30c-2-3p | 3'UTR |
|  | cg02002551 | Body;5'UTR | S_Shore |  |  |
|  | cg02106941 | TSS1500;1stExon | Island |  |  |
|  | cg02761376 | TSS1500;1stExon | Island |  |  |
|  | cg03012544 | Body | S_Shore |  |  |
|  | cg03169018 | Body;TSS200 | Island |  |  |
|  | cg04118306 | 1stExon;TSS200 | Island |  |  |
|  | cg07502389 | TSS1500;TSS200 | Island |  |  |
|  | cg07552803 | TSS1500;1stExon | Island |  |  |
|  | cg09234518 | TSS1500;TSS200 | Island |  |  |
|  | cg12026749 | Body;5'UTR | S_Shore |  |  |
|  | cg13387869 | 3'UTR | S_Shelf |  |  |
|  | cg16459364 | TSS1500;TSS200 | Island |  |  |
|  | cg17078116 | 1stExon;TSS200 | Island |  |  |
|  | cg18267374 | 5'UTR;TSS1500;1stExon | Island |  |  |
|  | cg18898125 | TSS1500 | N_Shore |  |  |
|  | cg19677607 | 1stExon;TSS200 | Island |  |  |
|  | cg20585869 | 1stExon;TSS200 | Island |  |  |
|  | cg22562942 | 1stExon;TSS200 | Island |  |  |
|  | cg23290344 | TSS1500;1stExon | Island |  |  |
|  | cg24705551 | Body | S_Shelf |  |  |
|  | cg26330518 | TSS1500 | N_Shore |  |  |
|  | cg26980244 | Body;5'UTR;1stExon | Island |  |  |
|  | cg27475652 | Body | S_Shelf |  |  |
| ABCC3 | cg00081975 | Body | Island | hsa-miR-139-3p | CDS |
|  | cg00126959 | TSS200 | Island |  |  |
|  | cg01054938 | Body | S_Shore |  |  |
|  | cg03965044 | TSS200 | Island |  |  |
|  | cg05599550 | TSS1500 | N_Shore |  |  |
|  | cg08014994 | TSS200 | Island |  |  |
|  | cg13725958 | 5'UTR;1stExon | Island |  |  |
|  | cg18312989 | Body | OpenSea |  |  |
|  | cg18426477 | Body | OpenSea |  |  |
|  | cg19248656 | TSS200 | Island |  |  |
|  | cg19643390 | TSS200 | Island |  |  |
|  | cg19734752 | Body | OpenSea |  |  |
|  | cg20633883 | TSS200 | Island |  |  |
|  | cg23340875 | TSS1500 | N_Shore |  |  |
|  | cg25928474 | Body | S_Shelf |  |  |
|  | cg26623266 | Body;3'UTR | OpenSea |  |  |
|  | cg27222669 | TSS1500 | N_Shore |  |  |
| WDR64 | cg05940691 | Body | OpenSea | hsa-let-7c-5p | CDS |
|  | cg06082598 | Body | OpenSea |  |  |
|  | cg07233230 | Body | OpenSea |  |  |
|  | cg22338443 | TSS1500 | OpenSea |  |  |
|  | cg26823144 | 3'UTR | OpenSea |  |  |
| TMEM177 | cg00049286 | TSS200 | Island | hsa-miR-195-5p | CDS |
|  | cg03768001 | TSS200 | Island |  |  |
|  | cg06607620 | Body | S_Shore |  |  |
|  | cg06886896 | TSS200 | Island |  |  |
|  | cg11344025 | 5'UTR;1stExon | Island |  |  |
|  | cg12108912 | TSS1500 | N_Shore |  |  |
|  | cg15974183 | TSS200 | Island |  |  |
|  | cg20302082 | TSS1500 | Island |  |  |
|  | cg22580372 | 5'UTR;1stExon | Island |  |  |
|  | cg24441810 | TSS1500 | N_Shore |  |  |
| CYP11B1 | cg02318454 | 1stExon | OpenSea | hsa-miR-30a-3p | 3'UTR |
|  | cg05416055 | Body | OpenSea |  |  |
|  | cg08230698 | Body | OpenSea |  |  |
|  | cg08389266 | 3'UTR | OpenSea |  |  |
|  | cg09120035 | 1stExon | OpenSea |  |  |
|  | cg14149685 | Body | OpenSea |  |  |
|  | cg17015994 | TSS200 | OpenSea |  |  |
|  | cg20073007 | Body | OpenSea |  |  |
|  | cg21298978 | TSS200 | OpenSea |  |  |
|  | cg21901156 | TSS200 | OpenSea |  |  |
|  | cg25376393 | Body | OpenSea |  |  |
| HABP2 | cg08404354 | TSS1500 | OpenSea | hsa-miR-139-3p | 5'UTR |
|  | cg14840429 | TSS1500 | OpenSea |  |  |
|  | cg18511011 | 3'UTR | OpenSea |  |  |
|  | cg18833140 | TSS1500 | OpenSea |  |  |
|  | cg26656452 | Body | OpenSea |  |  |
| SLC17A1 | cg03835296 | 5'UTR | OpenSea | hsa-miR-30c-2-3p | CDS |
|  | cg06885175 | 3'UTR | OpenSea |  |  |
|  | cg16251647 | 1stExon;5'UTR | OpenSea |  |  |
|  | cg22101098 | 5'UTR | OpenSea |  |  |
|  | cg22988581 | TSS200 | OpenSea |  |  |
|  | cg24766398 | 5'UTR | OpenSea |  |  |
| ZNF80 | cg03109316 | TSS1500 | OpenSea | hsa-miR-4529-3p | 3'UTR |
|  | cg10454162 | 5'UTR;1stExon | OpenSea |  |  |
|  | cg11380748 | TSS1500 | OpenSea |  |  |
|  | cg19477247 | 5'UTR;1stExon | OpenSea |  |  |
|  | cg22805431 | 1stExon | OpenSea |  |  |
| CNTNAP4 | cg01151029 | Body;TSS1500 | OpenSea | hsa-let-7c-5p | 3'UTR |
|  | cg04215511 | TSS1500 | OpenSea |  |  |
|  | cg05630943 | Body | OpenSea |  |  |
|  | cg08487455 | TSS200 | OpenSea |  |  |
|  | cg09094037 | Body;TSS1500 | OpenSea |  |  |
|  | cg09966303 | Body;TSS1500 | OpenSea |  |  |
|  | cg26577454 | 5'UTR;1stExon | OpenSea |  |  |
|  | cg27420415 | Body | OpenSea |  |  |
| ZNF217 | cg01692482 | 1stExon | Island | hsa-miR-378e | 3'UTR |
|  | cg04308938 | 3'UTR | OpenSea |  |  |
|  | cg05359207 | Body | N_Shore |  |  |
|  | cg07617814 | 1stExon | Island |  |  |
|  | cg08445782 | Body | N_Shelf |  |  |
|  | cg09029902 | 1stExon;5'UTR | S_Shore |  |  |
|  | cg09228833 | TSS200 | S_Shore |  |  |
|  | cg12032027 | 1stExon | Island |  |  |
|  | cg17098965 | 1stExon;5'UTR | S_Shore |  |  |
|  | cg20979153 | TSS200 | S_Shore |  |  |
| MAGEC2 | cg03793368 | TSS1500 | OpenSea | hsa-miR-4529-3p | 3'UTR |
|  | cg04064672 | 3'UTR | OpenSea |  |  |
|  | cg05257372 | Body | OpenSea |  |  |
|  | cg07549474 | TSS1500 | OpenSea |  |  |
|  | cg09929941 | TSS200 | OpenSea |  |  |
|  | cg10739728 | TSS200 | OpenSea |  |  |
|  | cg15602735 | TSS1500 | OpenSea |  |  |
|  | cg20333412 | TSS200 | OpenSea |  |  |
|  | cg23813564 | 5'UTR;1stExon | OpenSea |  |  |
|  | cg26465522 | TSS200 | OpenSea |  |  |
| GAGE1 | cg21262652 | Body;3'UTR | OpenSea | hsa-miR-144-3p | 3'UTR |
| POF1B | cg04704683 | Body | OpenSea | hsa-miR-1-3p | 3'UTR |
|  | cg05190630 | Body | OpenSea |  |  |
|  | cg08238865 | 5'UTR | OpenSea |  |  |
|  | cg10907112 | TSS200 | OpenSea |  |  |
|  | cg20017658 | 5'UTR | OpenSea |  |  |
|  | cg23455641 | Body | OpenSea |  |  |
|  | cg24387818 | TSS200 | OpenSea |  |  |
|  | cg25323709 | Body | OpenSea |  |  |
| VMP1 | cg00002749 | Body | OpenSea | hsa-miR-143-3p | 3'UTR |
|  | cg01409343 | Body | OpenSea |  |  |
|  | cg02766539 | Body | OpenSea |  |  |
|  | cg02782634 | Body | OpenSea |  |  |
|  | cg03823539 | 1stExon;5'UTR | Island |  |  |
|  | cg05265306 | TSS1500 | N_Shore |  |  |
|  | cg05371867 | TSS200 | Island |  |  |
|  | cg06747916 | TSS200 | Island |  |  |
|  | cg07852793 | TSS200 | Island |  |  |
|  | cg08793459 | TSS1500 | N_Shore |  |  |
|  | cg11251069 | 1stExon;5'UTR | Island |  |  |
|  | cg11698899 | 1stExon;5'UTR | Island |  |  |
|  | cg12054453 | Body | OpenSea |  |  |
|  | cg12395919 | 5'UTR | S_Shelf |  |  |
|  | cg13655923 | TSS1500 | N_Shore |  |  |
|  | cg14032089 | 3'UTR | OpenSea |  |  |
|  | cg16936953 | Body | OpenSea |  |  |
|  | cg17544904 | 1stExon;5'UTR | Island |  |  |
|  | cg18942579 | Body | OpenSea |  |  |
|  | cg20202881 | TSS200 | Island |  |  |
|  | cg20458044 | Body | OpenSea |  |  |
|  | cg23838308 | 1stExon;5'UTR | Island |  |  |
|  | cg24174557 | Body | OpenSea |  |  |
|  | cg25953464 | TSS200 | Island |  |  |
|  | cg26671554 | TSS200 | N_Shore |  |  |
|  | cg27298559 | 5'UTR | S_Shore |  |  |
| TRIML2 | cg03652810 | TSS1500 | N_Shore | hsa-miR-139-3p | CDS |
|  | cg11765851 | Body | N_Shore |  |  |
|  | cg17565478 | TSS1500 | N_Shore |  |  |
|  | cg21529532 | 3'UTR | OpenSea |  |  |
| CCL7 | cg01520122 | TSS1500 | OpenSea | hsa-miR-195-5p | CDS |
|  | cg02936263 | TSS1500 | OpenSea |  |  |
|  | cg08124722 | Body | OpenSea |  |  |
| AXDND1 | cg00255405 | 5'UTR | S_Shore | hsa-let-7c-5p | CDS |
|  | cg03440799 | TSS1500 | N_Shore |  |  |
|  | cg03732293 | 5'UTR;TSS200;1stExon | Island |  |  |
|  | cg04076212 | 5'UTR | S_Shore |  |  |
|  | cg04348247 | TSS1500 | N_Shore |  |  |
|  | cg08037922 | Body | OpenSea |  |  |
|  | cg09822482 | Body | OpenSea |  |  |
|  | cg19422340 | 5'UTR;TSS200;1stExon | Island |  |  |
|  | cg20746459 | Body | OpenSea |  |  |
|  | cg21538511 | 5'UTR | Island |  |  |
|  | cg22225298 | Body | S_Shelf |  |  |
| PRKAA2 | cg04876835 | 1stExon | Island | hsa-miR-139-3p | 3'UTR |
|  | cg06836772 | TSS1500 | N_Shore |  |  |
|  | cg09686317 | 1stExon | Island |  |  |
|  | cg09763175 | TSS1500 | Island |  |  |
|  | cg09935045 | Body | Island |  |  |
|  | cg12167830 | 1stExon | Island |  |  |
|  | cg19083779 | TSS1500 | Island |  |  |
|  | cg25617691 | TSS1500 | Island |  |  |
|  | cg26052885 | 3'UTR | OpenSea |  |  |
|  | cg26068551 | 1stExon | Island |  |  |
| AZGP1 | cg10372302 | TSS1500 | OpenSea | hsa-miR-139-3p | CDS |
|  | cg12019109 | TSS1500 | OpenSea |  |  |
|  | cg19237600 | 3'UTR | OpenSea |  |  |
|  | cg19465374 | Body | OpenSea |  |  |
|  | cg26429636 | TSS200 | OpenSea |  |  |
|  | cg26680675 | TSS1500 | OpenSea |  |  |
| IKBKE | cg06541188 | TSS200 | OpenSea | hsa-miR-30a-3p | CDS |
|  | cg11166312 | Body | OpenSea |  |  |
|  | cg11739399 | 3'UTR | OpenSea |  |  |
|  | cg14283602 | 5'UTR;1stExon | OpenSea |  |  |
|  | cg16556166 | Body | OpenSea |  |  |
|  | cg16619193 | TSS1500 | OpenSea |  |  |
|  | cg20530056 | TSS1500 | OpenSea |  |  |
|  | cg21875946 | Body | OpenSea |  |  |
|  | cg22577136 | 5'UTR | OpenSea |  |  |
|  | cg26859016 | TSS1500 | OpenSea |  |  |
|  | cg26912602 | 5'UTR | OpenSea |  |  |
| S100A7A | cg11790580 | 5'UTR | OpenSea | hsa-let-7c-5p | 3'UTR |
|  | cg12067024 | TSS1500 | OpenSea |  |  |
|  | cg17496887 | TSS1500 | OpenSea |  |  |
|  | cg21107767 | TSS200 | OpenSea |  |  |
|  | cg22933439 | TSS1500 | OpenSea |  |  |
|  | cg24255159 | 5'UTR | OpenSea |  |  |
| ATP1B1 | cg04405414 | TSS1500 | Island | hsa-miR-30c-2-3p | 3'UTR |
|  | cg06176471 | Body | OpenSea |  |  |
|  | cg07136905 | TSS1500 | N_Shore |  |  |
|  | cg08785155 | Body | Island |  |  |
|  | cg12236822 | 1stExon | Island |  |  |
|  | cg13104274 | Body | S_Shore |  |  |
|  | cg13184582 | TSS1500 | Island |  |  |
|  | cg16959606 | TSS1500 | Island |  |  |
|  | cg18159230 | 5'UTR;1stExon | Island |  |  |
|  | cg22399423 | TSS1500 | Island |  |  |
|  | cg24229334 | TSS1500 | Island |  |  |
|  | cg24304617 | Body | S_Shelf |  |  |
|  | cg26009832 | Body | OpenSea |  |  |
|  | cg26856322 | 5'UTR;1stExon | Island |  |  |
|  | ch.1.3307476R | Body | OpenSea |  |  |
| ZNF716 | cg01098474 | TSS1500 | OpenSea | hsa-let-7c-5p | 3'UTR |
|  | cg01545952 | Body | OpenSea |  |  |
|  | cg03298312 | 5'UTR;1stExon | OpenSea |  |  |
|  | cg09299053 | TSS200 | OpenSea |  |  |
|  | cg11357940 | Body | OpenSea |  |  |
|  | cg19521511 | TSS200 | OpenSea |  |  |
| *Down-regulated genes affected by both high miRNA and hypermethylation* |  |  |  |  |  |
| FAT4 | cg00990763 | TSS1500 | Island | hsa-let-7c-5p | 5'UTR |
|  | cg03404279 | 1stExon | Island |  |  |
|  | cg03527919 | TSS200 | Island |  |  |
|  | cg04023369 | TSS1500 | Island |  |  |
|  | cg04373334 | 1stExon | Island |  |  |
|  | cg04459504 | TSS1500 | Island |  |  |
|  | cg05118638 | TSS200 | Island |  |  |
|  | cg08575049 | 1stExon | Island |  |  |
|  | cg08644023 | TSS200 | Island |  |  |
|  | cg10399929 | 1stExon | S_Shore |  |  |
|  | cg10731073 | TSS200 | Island |  |  |
|  | cg12058185 | 1stExon | Island |  |  |
|  | cg12828819 | TSS1500 | Island |  |  |
|  | cg13742182 | TSS1500 | Island |  |  |
|  | cg15795630 | TSS200 | Island |  |  |
|  | cg17265829 | 1stExon | Island |  |  |
|  | cg18202623 | TSS1500 | Island |  |  |
|  | cg22911422 | 1stExon | Island |  |  |
|  | cg23901852 | 1stExon | Island |  |  |
|  | cg25879360 | 1stExon | S_Shelf |  |  |
|  | cg26389756 | Body | OpenSea |  |  |
| KLF4 | cg00492574 | Body | N_Shore | hsa-miR-767-5p | 3'UTR |
|  | cg01564322 | Body | Island |  |  |
|  | cg03066050 | Body | Island |  |  |
|  | cg03267342 | TSS200 | Island |  |  |
|  | cg03939688 | 5'UTR;1stExon | Island |  |  |
|  | cg06545019 | Body | Island |  |  |
|  | cg07309102 | 5'UTR;1stExon | Island |  |  |
|  | cg13894301 | TSS1500 | Island |  |  |
|  | cg14185918 | Body | Island |  |  |
| EPB41L3 | cg00027083 | 5'UTR | Island | hsa-miR-127-5p | 3'UTR |
|  | cg01673082 | TSS200 | Island |  |  |
|  | cg02926160 | 5'UTR | N_Shore |  |  |
|  | cg06459104 | Body | OpenSea |  |  |
|  | cg06550462 | TSS1500 | Island |  |  |
|  | cg07352438 | 5'UTR | Island |  |  |
|  | cg12967001 | TSS200 | Island |  |  |
|  | cg13034362 | TSS1500 | S_Shore |  |  |
|  | cg14075742 | 5'UTR | N_Shelf |  |  |
|  | cg16304950 | 5'UTR;1stExon | Island |  |  |
|  | cg16622495 | TSS200 | Island |  |  |
|  | cg16924702 | TSS1500 | Island |  |  |
|  | cg18543270 | 5'UTR | N_Shore |  |  |
|  | cg19579167 | 5'UTR | Island |  |  |
|  | cg22335490 | TSS1500 | Island |  |  |
|  | cg23564700 | TSS200 | Island |  |  |
|  | cg23847381 | TSS200 | Island |  |  |
|  | cg26790372 | 5'UTR | Island |  |  |
|  | cg27082185 | 5'UTR | N_Shelf |  |  |
| NR4A3 | cg01917626 | 5'UTR | Island | hsa-miR-105-5p | 3'UTR |
|  | cg03277560 | 5'UTR | Island |  |  |
|  | cg04897621 | 5'UTR | Island |  |  |
|  | cg05933762 | 5'UTR | Island |  |  |
|  | cg06145669 | Body;5'UTR | N_Shore |  |  |
|  | cg08042258 | Body | Island |  |  |
|  | cg12190044 | Body | Island |  |  |
|  | cg13412395 | TSS1500 | Island |  |  |
|  | cg13480493 | Body | OpenSea |  |  |
|  | cg13636189 | 5'UTR | Island |  |  |
|  | cg13639936 | 5'UTR | Island |  |  |
|  | cg13655635 | 5'UTR;TSS1500 | S_Shore |  |  |
|  | cg13666703 | TSS1500 | Island |  |  |
|  | cg13703070 | TSS1500 | Island |  |  |
|  | cg13770982 | 5'UTR | Island |  |  |
|  | cg13796392 | TSS1500 | Island |  |  |
|  | cg14569576 | 5'UTR | Island |  |  |
|  | cg16160550 | TSS1500 | Island |  |  |
|  | cg21205987 | 5'UTR;1stExon | Island |  |  |
|  | cg25454755 | 5'UTR;1stExon | Island |  |  |
| SYNPO2L | cg00560542 | Body | Island | hsa-miR-182-5p | 3'UTR |
|  | cg02286717 | 5'UTR;1stExon | OpenSea |  |  |
|  | cg05697785 | TSS1500 | OpenSea |  |  |
|  | cg07127957 | Body;TSS1500 | S_Shelf |  |  |
|  | cg07671858 | Body | Island |  |  |
|  | cg09521743 | 5'UTR;1stExon | OpenSea |  |  |
|  | cg12291247 | Body;TSS1500 | S_Shelf |  |  |
|  | cg12317414 | Body | S_Shore |  |  |
|  | cg15297724 | Body | Island |  |  |
|  | cg16228286 | Body | N_Shore |  |  |
|  | cg17411546 | Body | S_Shelf |  |  |
|  | cg17723653 | Body | S_Shore |  |  |
|  | cg19154600 | TSS200 | OpenSea |  |  |
|  | cg19157819 | 3'UTR | N_Shelf |  |  |
|  | cg23083424 | TSS200 | OpenSea |  |  |
|  | cg24637261 | Body;TSS200 | S_Shelf |  |  |
|  | cg25736617 | Body;TSS1500 | S_Shelf |  |  |
|  | cg26549892 | TSS1500 | OpenSea |  |  |
|  | cg27550918 | Body;5'UTR;1stExon | S_Shelf |  |  |
| SLC5A7 | cg03983336 | TSS1500 | N_Shore | hsa-miR-139-3p | 5'UTR |
|  | cg05099596 | Body | S_Shore |  |  |
|  | cg05311412 | TSS200 | Island |  |  |
|  | cg06662812 | 5'UTR | Island |  |  |
|  | cg07186154 | 5'UTR | Island |  |  |
|  | cg11355135 | TSS200 | Island |  |  |
|  | cg14613271 | TSS200 | Island |  |  |
|  | cg14772660 | Body | S_Shelf |  |  |
|  | cg16232126 | 5'UTR;1stExon | Island |  |  |
|  | cg17052964 | TSS1500 | N_Shore |  |  |
|  | cg17679621 | TSS1500 | N_Shore |  |  |
|  | cg18771173 | TSS200 | Island |  |  |
|  | cg20547653 | 5'UTR | Island |  |  |
|  | cg22415472 | TSS1500 | N_Shore |  |  |
|  | cg26001902 | TSS200 | Island |  |  |
|  | cg26333822 | 5'UTR;1stExon | Island |  |  |
| PAX6 | cg00060304 | 5'UTR | Island | hsa-miR-127-5p | 3'UTR |
|  | cg01587682 | Body | Island |  |  |
|  | cg01665555 | TSS1500;5'UTR | Island |  |  |
|  | cg01867395 | TSS200 | Island |  |  |
|  | cg01904410 | 5'UTR | Island |  |  |
|  | cg02468250 | TSS1500;5'UTR | Island |  |  |
|  | cg02642123 | 5'UTR | Island |  |  |
|  | cg02679809 | 5'UTR | Island |  |  |
|  | cg02770983 | Body | S_Shore |  |  |
|  | cg02771142 | Body | N_Shore |  |  |
|  | cg03527353 | 5'UTR;1stExon | Island |  |  |
|  | cg03854796 | Body | N_Shore |  |  |
|  | cg03905867 | Body | Island |  |  |
|  | cg04504066 | 5'UTR | S_Shore |  |  |
|  | cg04521333 | Body | N_Shore |  |  |
|  | cg04598774 | Body | S_Shore |  |  |
|  | cg04938549 | Body | N_Shore |  |  |
|  | cg05091519 | TSS200 | Island |  |  |
|  | cg05490712 | 5'UTR | Island |  |  |
|  | cg05840031 | 5'UTR | Island |  |  |
|  | cg06312283 | TSS1500 | N_Shore |  |  |
|  | cg06666008 | Body | N_Shore |  |  |
|  | cg06705930 | Body | S_Shore |  |  |
|  | cg07124117 | Body | Island |  |  |
|  | cg07434271 | TSS1500 | N_Shore |  |  |
|  | cg07478918 | Body | N_Shelf |  |  |
|  | cg07660750 | 5'UTR | S_Shore |  |  |
|  | cg07905944 | 5'UTR | Island |  |  |
|  | cg08005992 | 5'UTR;TSS200 | Island |  |  |
|  | cg08116462 | Body | S_Shore |  |  |
|  | cg08391415 | 5'UTR | Island |  |  |
|  | cg08499046 | Body | N_Shore |  |  |
|  | cg08784129 | Body | N_Shore |  |  |
|  | cg09041678 | TSS1500 | N_Shore |  |  |
|  | cg09217215 | 5'UTR | S_Shore |  |  |
|  | cg09224689 | Body | N_Shore |  |  |
|  | cg09252999 | 5'UTR | Island |  |  |
|  | cg09382096 | Body | N_Shore |  |  |
|  | cg09537620 | Body | Island |  |  |
|  | cg09656389 | TSS1500 | Island |  |  |
|  | cg10177238 | TSS1500;5'UTR | Island |  |  |
|  | cg11128216 | Body | Island |  |  |
|  | cg11162118 | 5'UTR | Island |  |  |
|  | cg11333459 | 5'UTR | Island |  |  |
|  | cg11469061 | Body | Island |  |  |
|  | cg11482099 | TSS1500;5'UTR | Island |  |  |
|  | cg11827910 | Body | Island |  |  |
|  | cg12086936 | 5'UTR;TSS200 | Island |  |  |
|  | cg12798259 | Body | S_Shore |  |  |
|  | cg13028700 | 5'UTR | Island |  |  |
|  | cg13245152 | Body | S_Shore |  |  |
|  | cg13570972 | TSS200 | Island |  |  |
|  | cg13596833 | Body | Island |  |  |
|  | cg13694576 | 5'UTR | Island |  |  |
|  | cg13891702 | Body | N_Shore |  |  |
|  | cg14002345 | 5'UTR;TSS200 | Island |  |  |
|  | cg14037837 | 5'UTR | N_Shore |  |  |
|  | cg14293548 | 3'UTR | OpenSea |  |  |
|  | cg14439629 | 5'UTR | S_Shore |  |  |
|  | cg14443953 | 5'UTR | Island |  |  |
|  | cg14800351 | Body | N_Shelf |  |  |
|  | cg15301794 | Body | S_Shore |  |  |
|  | cg15778437 | TSS200 | Island |  |  |
|  | cg16113298 | 5'UTR;1stExon | Island |  |  |
|  | cg16180353 | Body | N_Shore |  |  |
|  | cg16615954 | Body | Island |  |  |
|  | cg16616521 | Body | Island |  |  |
|  | cg16822387 | 5'UTR | N_Shore |  |  |
|  | cg16865446 | Body | S_Shore |  |  |
|  | cg17280740 | TSS1500 | N_Shore |  |  |
|  | cg18058532 | 5'UTR;1stExon | Island |  |  |
|  | cg18082638 | Body | Island |  |  |
|  | cg18270629 | 5'UTR | Island |  |  |
|  | cg18372607 | Body | N_Shore |  |  |
|  | cg18400845 | 5'UTR | Island |  |  |
|  | cg18988498 | Body | N_Shore |  |  |
|  | cg19006378 | 5'UTR | S_Shore |  |  |
|  | cg20014398 | Body | Island |  |  |
|  | cg20131194 | 5'UTR;TSS200 | Island |  |  |
|  | cg20528093 | 5'UTR | Island |  |  |
|  | cg21016855 | Body | N_Shore |  |  |
|  | cg21582373 | Body | N_Shore |  |  |
|  | cg21764190 | 5'UTR | Island |  |  |
|  | cg22272457 | 5'UTR;TSS200 | Island |  |  |
|  | cg22392038 | Body | S_Shore |  |  |
|  | cg22557091 | Body | Island |  |  |
|  | cg22740492 | Body | Island |  |  |
|  | cg22982368 | Body | Island |  |  |
|  | cg23125492 | Body | Island |  |  |
|  | cg23287710 | Body | S_Shore |  |  |
|  | cg23484599 | 5'UTR | N_Shore |  |  |
|  | cg23933618 | 5'UTR | Island |  |  |
|  | cg24332783 | Body | N_Shore |  |  |
|  | cg25242557 | TSS1500;5'UTR | Island |  |  |
|  | cg25603277 | 5'UTR | Island |  |  |
|  | cg25764105 | Body | N_Shelf |  |  |
|  | cg25949958 | 5'UTR;TSS200 | Island |  |  |
|  | cg26019112 | Body | Island |  |  |
|  | cg26029734 | Body | N_Shore |  |  |
|  | cg26315277 | TSS200 | Island |  |  |
|  | cg26848086 | 5'UTR | Island |  |  |
|  | cg26932432 | 5'UTR | N_Shore |  |  |
|  | cg27011060 | Body | N_Shore |  |  |
|  | cg27597956 | 5'UTR;1stExon | Island |  |  |
| FGF10 | cg00167491 | TSS200 | OpenSea | hsa-miR-196a-5p | 3'UTR |
|  | cg00272200 | TSS200 | OpenSea |  |  |
|  | cg02443428 | TSS200 | OpenSea |  |  |
|  | cg06850283 | TSS1500 | OpenSea |  |  |
|  | cg08976810 | TSS1500 | OpenSea |  |  |
|  | cg09792008 | TSS1500 | OpenSea |  |  |
|  | cg13996155 | TSS1500 | OpenSea |  |  |
|  | cg14654926 | 1stExon | OpenSea |  |  |
|  | cg16204420 | Body | OpenSea |  |  |
|  | cg16384588 | TSS200 | OpenSea |  |  |
|  | cg16536718 | TSS1500 | OpenSea |  |  |
|  | cg20387341 | TSS1500 | OpenSea |  |  |
|  | cg22574802 | 1stExon | OpenSea |  |  |
|  | cg25103492 | TSS200 | OpenSea |  |  |
| RSPH10B | cg00318111 | 5'UTR | Island | hsa-miR-105-5p | 3'UTR |
|  | cg00612714 | Body | N_Shelf |  |  |
|  | cg10388456 | 5'UTR | Island |  |  |
|  | cg11051318 | Body | OpenSea |  |  |
|  | cg15703585 | Body | Island |  |  |
|  | cg19694460 | Body | S_Shelf |  |  |
|  | cg25707767 | 5'UTR | Island |  |  |
|  | cg27084028 | Body | OpenSea |  |  |
| SLIT2 | cg00749969 | Body | OpenSea | hsa-miR-139-3p | 5'UTR |
|  | cg03260566 | Body | Island |  |  |
|  | cg03742003 | 1stExon;5'UTR | Island |  |  |
|  | cg03790250 | Body | Island |  |  |
|  | cg07290920 | Body | OpenSea |  |  |
|  | cg07711036 | Body | OpenSea |  |  |
|  | cg08415391 | Body | OpenSea |  |  |
|  | cg08428452 | 1stExon;5'UTR | Island |  |  |
|  | cg09781944 | TSS1500 | Island |  |  |
|  | cg10603296 | Body | OpenSea |  |  |
|  | cg10947633 | Body | OpenSea |  |  |
|  | cg13078140 | TSS200 | Island |  |  |
|  | cg13281139 | TSS200 | Island |  |  |
|  | cg13485685 | Body | Island |  |  |
|  | cg15469350 | TSS200 | Island |  |  |
|  | cg18972811 | TSS1500 | Island |  |  |
|  | cg19940312 | Body | S_Shore |  |  |
|  | cg20928234 | Body | Island |  |  |
|  | cg21129041 | Body | OpenSea |  |  |
|  | cg22508905 | Body | OpenSea |  |  |
|  | cg23412793 | TSS1500 | Island |  |  |
|  | cg26164897 | Body | S_Shelf |  |  |
|  | cg26854559 | Body | OpenSea |  |  |
| NDST1 | cg02562005 | Body | Island | hsa-miR-105-5p | 3'UTR |
|  | cg03111114 | TSS1500 | OpenSea |  |  |
|  | cg03345059 | Body | Island |  |  |
|  | cg04091768 | 5'UTR | OpenSea |  |  |
|  | cg06677890 | TSS200 | OpenSea |  |  |
|  | cg07672051 | 5'UTR;1stExon | OpenSea |  |  |
|  | cg09028166 | 5'UTR | OpenSea |  |  |
|  | cg12557254 | Body | S_Shore |  |  |
|  | cg12589486 | Body | N_Shelf |  |  |
|  | cg14873515 | TSS1500 | OpenSea |  |  |
|  | cg15337815 | 3'UTR | OpenSea |  |  |
|  | cg16674484 | TSS200 | OpenSea |  |  |
|  | cg19836174 | Body | OpenSea |  |  |
|  | cg22157525 | Body | OpenSea |  |  |
|  | cg22540600 | Body | Island |  |  |
|  | cg24153754 | Body | OpenSea |  |  |
|  | cg24884265 | Body | Island |  |  |
|  | cg25677709 | 5'UTR | OpenSea |  |  |
| LRRC4 | cg00353923 | TSS200 | N_Shore | hsa-miR-490-5p | 5'UTR |
|  | cg02176148 | Body | N_Shore |  |  |
|  | cg04153784 | TSS1500 | Island |  |  |
|  | cg06173720 | 5'UTR;1stExon | N_Shore |  |  |
|  | cg06945399 | TSS200 | Island |  |  |
|  | cg07871590 | TSS200 | Island |  |  |
|  | cg09087503 | TSS1500 | Island |  |  |
|  | cg12628196 | TSS1500 | Island |  |  |
|  | cg13508402 | Body | N_Shore |  |  |
|  | cg14059988 | Body | N_Shore |  |  |
|  | cg15087147 | TSS1500 | Island |  |  |
|  | cg21129531 | 5'UTR | N_Shore |  |  |
|  | cg21579556 | 3'UTR | N_Shelf |  |  |
|  | cg24084481 | Body | N_Shore |  |  |
|  | cg26173847 | TSS200 | N_Shore |  |  |
|  | cg26595520 | TSS200 | N_Shore |  |  |
|  | cg27573591 | TSS1500 | Island |  |  |
| MAL | cg03129884 | Body | S_Shore | hsa-miR-21-3p | 3'UTR |
|  | cg03566174 | Body | OpenSea |  |  |
|  | cg04804539 | TSS1500 | Island |  |  |
|  | cg05142617 | TSS1500 | N_Shore |  |  |
|  | cg05314420 | TSS1500 | N_Shore |  |  |
|  | cg06668300 | Body | Island |  |  |
|  | cg07224914 | Body | Island |  |  |
|  | cg09712683 | Body | Island |  |  |
|  | cg09983051 | TSS1500 | Island |  |  |
|  | cg10108468 | 3'UTR | OpenSea |  |  |
|  | cg14410476 | Body | S_Shore |  |  |
|  | cg17626405 | Body | Island |  |  |
|  | cg19762657 | Body | S_Shore |  |  |
|  | cg21245652 | TSS1500 | Island |  |  |
|  | cg22403344 | 5'UTR;1stExon | Island |  |  |
|  | cg22762844 | Body | Island |  |  |
| EDNRB | cg01910869 | TSS1500;5'UTR | Island | hsa-miR-127-5p | 3'UTR |
|  | cg02147695 | TSS1500;5'UTR | S_Shore |  |  |
|  | cg03086857 | Body;1stExon | Island |  |  |
|  | cg04390523 | 5'UTR;TSS200 | Island |  |  |
|  | cg06057566 | TSS1500;5'UTR | S_Shore |  |  |
|  | cg06106063 | TSS1500 | OpenSea |  |  |
|  | cg06179060 | 5'UTR;TSS200 | Island |  |  |
|  | cg06971129 | 5'UTR;TSS200 | Island |  |  |
|  | cg07035515 | TSS1500;5'UTR | S_Shore |  |  |
|  | cg07495027 | Body | N_Shore |  |  |
|  | cg07974719 | TSS1500;5'UTR | S_Shore |  |  |
|  | cg08321129 | TSS1500;5'UTR | S_Shore |  |  |
|  | cg08634041 | TSS1500;5'UTR | S_Shore |  |  |
|  | cg09152886 | TSS1500;5'UTR | S_Shore |  |  |
|  | cg09786383 | TSS1500;5'UTR | S_Shore |  |  |
|  | cg10016380 | TSS1500;5'UTR | Island |  |  |
|  | cg10764762 | TSS1500;5'UTR | S_Shore |  |  |
|  | cg10792120 | TSS1500 | OpenSea |  |  |
|  | cg11074192 | TSS1500;5'UTR | S_Shore |  |  |
|  | cg12120741 | Body;1stExon | N_Shore |  |  |
|  | cg12321193 | TSS1500 | OpenSea |  |  |
|  | cg12602112 | TSS1500;5'UTR | Island |  |  |
|  | cg12847373 | TSS1500;5'UTR | Island |  |  |
|  | cg12935136 | TSS1500;5'UTR | S_Shore |  |  |
|  | cg12983394 | TSS1500;5'UTR | S_Shore |  |  |
|  | cg13434989 | TSS1500;5'UTR | Island |  |  |
|  | cg13818654 | 5'UTR;TSS200 | Island |  |  |
|  | cg13866767 | Body;3'UTR | OpenSea |  |  |
|  | cg15699226 | Body;1stExon | Island |  |  |
|  | cg15836660 | 5'UTR;TSS200 | Island |  |  |
|  | cg16203262 | 5'UTR | S_Shelf |  |  |
|  | cg16571983 | 5'UTR;1stExon | Island |  |  |
|  | cg16739796 | TSS1500;5'UTR | S_Shore |  |  |
|  | cg18032190 | TSS1500;5'UTR | Island |  |  |
|  | cg18210860 | Body;3'UTR | OpenSea |  |  |
|  | cg18568990 | TSS1500;5'UTR | S_Shore |  |  |
|  | cg19111971 | TSS1500;5'UTR | S_Shore |  |  |
|  | cg19650157 | TSS1500;5'UTR | Island |  |  |
|  | cg19742055 | TSS1500;5'UTR | Island |  |  |
|  | cg19759502 | TSS1500;5'UTR | S_Shore |  |  |
|  | cg19916212 | TSS1500;5'UTR | Island |  |  |
|  | cg21364111 | TSS1500;5'UTR | S_Shore |  |  |
|  | cg21675115 | TSS1500;5'UTR | S_Shore |  |  |
|  | cg22310279 | TSS1500;5'UTR | Island |  |  |
|  | cg22541679 | TSS1500;5'UTR | S_Shore |  |  |
|  | cg23316360 | 5'UTR;1stExon | Island |  |  |
|  | cg23326536 | Body | N_Shore |  |  |
|  | cg23494140 | TSS1500;5'UTR | S_Shore |  |  |
|  | cg23766591 | TSS1500;5'UTR | Island |  |  |
|  | cg24236409 | TSS1500;5'UTR | Island |  |  |
|  | cg24745738 | TSS1500;5'UTR | S_Shore |  |  |
|  | cg24785726 | Body | N_Shore |  |  |
|  | cg24942919 | TSS1500;5'UTR | S_Shore |  |  |
|  | cg25717994 | TSS1500;5'UTR | S_Shore |  |  |
|  | cg26022015 | TSS1500;5'UTR | S_Shore |  |  |
|  | cg26622320 | 5'UTR;1stExon | Island |  |  |
| CCRL2 | cg03477080 | TSS200;5'UTR;1stExon | OpenSea | hsa-miR-1269a | 3'UTR |
|  | cg05670596 | TSS1500 | OpenSea |  |  |
|  | cg08679238 | 5'UTR;1stExon | OpenSea |  |  |
|  | cg10952220 | TSS1500;TSS200 | OpenSea |  |  |
|  | cg12332536 | 3'UTR | OpenSea |  |  |
|  | cg12579212 | TSS200;5'UTR;1stExon | OpenSea |  |  |
|  | cg13070763 | TSS200;5'UTR;1stExon | OpenSea |  |  |
|  | cg14749678 | Body | OpenSea |  |  |
|  | cg14754581 | TSS1500;5'UTR;1stExon | OpenSea |  |  |
|  | cg18599081 | TSS1500 | OpenSea |  |  |
|  | cg19850333 | TSS1500;TSS200 | OpenSea |  |  |
|  | cg23350385 | TSS1500 | OpenSea |  |  |
|  | cg25358853 | TSS1500;TSS200 | OpenSea |  |  |
| PDPN | cg00831909 | TSS1500 | Island | hsa-miR-210-3p | 3'UTR |
|  | cg02933679 | TSS200 | Island |  |  |
|  | cg04886857 | Body;5'UTR | S_Shelf |  |  |
|  | cg05140069 | Body;TSS1500 | Island |  |  |
|  | cg05281894 | Body;TSS1500 | Island |  |  |
|  | cg09804380 | Body;TSS1500 | Island |  |  |
|  | cg10743104 | TSS200 | Island |  |  |
|  | cg11791751 | TSS200 | Island |  |  |
|  | cg14027957 | Body;5'UTR | OpenSea |  |  |
|  | cg15563963 | TSS200 | Island |  |  |
|  | cg16277479 | TSS200 | Island |  |  |
|  | cg16590190 | TSS1500 | N_Shore |  |  |
|  | cg17271677 | 3'UTR | OpenSea |  |  |
|  | cg17826518 | Body;5'UTR;1stExon | S_Shore |  |  |
|  | cg18877506 | TSS1500;1stExon | Island |  |  |
|  | cg21819468 | TSS1500;1stExon | Island |  |  |
|  | cg22436123 | TSS200 | Island |  |  |
|  | cg22798977 | TSS1500 | Island |  |  |
|  | cg23954416 | TSS1500 | N_Shore |  |  |
|  | cg24671344 | Body;TSS1500 | Island |  |  |
|  | cg25286482 | TSS1500 | Island |  |  |
| ID4 | cg00468146 | 1stExon | Island | hsa-miR-142-3p | 3'UTR |
|  | cg03715143 | 5'UTR;1stExon | Island |  |  |
|  | cg04359753 | 3'UTR | S_Shore |  |  |
|  | cg04999479 | 3'UTR | Island |  |  |
|  | cg09360571 | 3'UTR | S_Shore |  |  |
|  | cg14151259 | TSS1500 | Island |  |  |
|  | cg14271531 | 5'UTR;1stExon | Island |  |  |
|  | cg17252960 | TSS1500 | N_Shore |  |  |
|  | cg17305436 | TSS1500 | N_Shore |  |  |
|  | cg23165899 | TSS1500 | Island |  |  |
|  | cg23687194 | 3'UTR | S_Shore |  |  |
|  | cg24109612 | Body | Island |  |  |
| JAM2 | cg01975706 | Body | S_Shelf | hsa-miR-1269a | 3'UTR |
|  | cg02485200 | 5'UTR;1stExon | Island |  |  |
|  | cg03382304 | 1stExon | Island |  |  |
|  | cg04964944 | Body | S_Shore |  |  |
|  | cg05504541 | TSS200 | N_Shore |  |  |
|  | cg06479755 | Body | Island |  |  |
|  | cg06716684 | TSS200 | N_Shore |  |  |
|  | cg09704750 | TSS1500 | N_Shore |  |  |
|  | cg10376067 | 3'UTR | OpenSea |  |  |
|  | cg10466421 | TSS1500 | N_Shore |  |  |
|  | cg13769223 | 5'UTR;1stExon | Island |  |  |
|  | cg14553600 | 5'UTR;1stExon | Island |  |  |
|  | cg16532755 | TSS200 | N_Shore |  |  |
|  | cg21042970 | TSS200 | N_Shore |  |  |
|  | cg22627390 | 5'UTR;1stExon | Island |  |  |
|  | cg26412722 | Body | S_Shore |  |  |
| GPRASP1 | cg04225046 | TSS200 | Island | hsa-miR-1-3p | 5'UTR |
|  | cg04748497 | 5'UTR | Island |  |  |
|  | cg09278708 | TSS1500 | N_Shore |  |  |
|  | cg09332981 | TSS1500 | N_Shore |  |  |
|  | cg10670396 | 5'UTR | Island |  |  |
|  | cg12295100 | 5'UTR | Island |  |  |
|  | cg12869615 | TSS200 | Island |  |  |
|  | cg15230046 | Body | S_Shelf |  |  |
|  | cg15579650 | TSS200 | Island |  |  |
|  | cg18536496 | TSS1500 | Island |  |  |
|  | cg20207108 | TSS200 | Island |  |  |
|  | cg21232685 | Body | S_Shelf |  |  |
|  | cg23571457 | 5'UTR | S_Shore |  |  |
|  | cg24818939 | TSS200 | Island |  |  |
|  | cg25777540 | 1stExon;5'UTR | Island |  |  |
|  | cg25950739 | 5'UTR | S_Shore |  |  |
| KCNB1 | cg00429268 | Body | N_Shore | hsa-miR-182-5p | 3'UTR |
|  | cg00976453 | TSS1500 | Island |  |  |
|  | cg01445809 | TSS1500 | Island |  |  |
|  | cg01663603 | TSS200 | Island |  |  |
|  | cg04836214 | Body | N_Shore |  |  |
|  | cg05303999 | 3'UTR | OpenSea |  |  |
|  | cg10951120 | TSS200 | Island |  |  |
|  | cg11131532 | TSS200 | Island |  |  |
|  | cg12307787 | TSS200 | Island |  |  |
|  | cg12392473 | TSS200 | Island |  |  |
|  | cg14474561 | Body | N_Shelf |  |  |
|  | cg14736210 | 1stExon | Island |  |  |
|  | cg17445913 | TSS1500 | N_Shore |  |  |
|  | cg18597991 | Body | N_Shore |  |  |
|  | cg20912978 | Body | OpenSea |  |  |
|  | cg24507762 | TSS1500 | N_Shore |  |  |
|  | cg26709285 | 5'UTR;1stExon | Island |  |  |
| SULT1C4 | cg04414451 | TSS1500 | OpenSea | hsa-miR-1269a | 3'UTR |
|  | cg05036173 | 5'UTR;1stExon | OpenSea |  |  |
|  | cg10236239 | 5'UTR;1stExon | OpenSea |  |  |
|  | cg14300730 | TSS200 | OpenSea |  |  |
|  | cg17966192 | TSS1500 | OpenSea |  |  |
|  | cg19908768 | TSS200 | OpenSea |  |  |
| DKK2 | cg00594011 | 1stExon | Island | hsa-miR-127-5p | 3'UTR |
|  | cg01404615 | TSS1500 | S_Shore |  |  |
|  | cg01421943 | 3'UTR | OpenSea |  |  |
|  | cg01471384 | 5'UTR;1stExon | Island |  |  |
|  | cg01962428 | 5'UTR;1stExon | Island |  |  |
|  | cg03504865 | TSS1500 | S_Shore |  |  |
|  | cg03903991 | Body | N_Shore |  |  |
|  | cg04777988 | Body | N_Shelf |  |  |
|  | cg08341316 | 5'UTR;1stExon | Island |  |  |
|  | cg13139972 | 5'UTR;1stExon | Island |  |  |
|  | cg14129775 | TSS1500 | S_Shore |  |  |
|  | cg17307558 | Body | N_Shore |  |  |
|  | cg17362052 | Body | N_Shore |  |  |
|  | cg24874180 | Body | N_Shore |  |  |
|  | cg25262044 | TSS1500 | S_Shore |  |  |
| GHR | cg00394261 | 5'UTR | OpenSea | hsa-miR-1269a | 3'UTR |
|  | cg03836184 | 5'UTR | Island |  |  |
|  | cg04110544 | 5'UTR | Island |  |  |
|  | cg05837253 | 5'UTR | N_Shore |  |  |
|  | cg07237214 | 5'UTR | Island |  |  |
|  | cg08217227 | TSS1500 | Island |  |  |
|  | cg08761396 | 5'UTR | OpenSea |  |  |
|  | cg12042587 | TSS200 | N_Shore |  |  |
|  | cg12887711 | Body | OpenSea |  |  |
|  | cg14868574 | TSS1500 | Island |  |  |
|  | cg14972625 | 5'UTR | OpenSea |  |  |
|  | cg16292016 | 5'UTR | Island |  |  |
|  | cg18160072 | 5'UTR | Island |  |  |
|  | cg18304305 | 3'UTR | OpenSea |  |  |
|  | cg22016770 | 5'UTR | OpenSea |  |  |
|  | cg24773720 | 5'UTR | Island |  |  |
|  | cg24799710 | 5'UTR | OpenSea |  |  |
|  | cg25010146 | 5'UTR | Island |  |  |
|  | cg25430094 | TSS1500 | Island |  |  |
|  | cg26162554 | 5'UTR | S_Shore |  |  |
| ATOH8 | cg00400334 | 1stExon | Island | hsa-miR-127-5p | 3'UTR |
|  | cg00572504 | 3'UTR | OpenSea |  |  |
|  | cg01472882 | TSS1500 | Island |  |  |
|  | cg01751470 | TSS200 | Island |  |  |
|  | cg02317742 | 5'UTR;1stExon | Island |  |  |
|  | cg02914235 | Body | S_Shelf |  |  |
|  | cg03128635 | TSS200 | Island |  |  |
|  | cg05318142 | Body | OpenSea |  |  |
|  | cg06897686 | Body | Island |  |  |
|  | cg07622521 | Body | OpenSea |  |  |
|  | cg08079596 | Body | OpenSea |  |  |
|  | cg09662694 | TSS200 | Island |  |  |
|  | cg10389138 | Body | OpenSea |  |  |
|  | cg11726701 | Body | OpenSea |  |  |
|  | cg12930553 | TSS200 | Island |  |  |
|  | cg13065834 | 5'UTR;1stExon | Island |  |  |
|  | cg14141912 | Body | OpenSea |  |  |
|  | cg14558812 | Body | S_Shore |  |  |
|  | cg18815025 | TSS200 | Island |  |  |
|  | cg19956166 | TSS200 | Island |  |  |
|  | cg20839205 | Body | OpenSea |  |  |
|  | cg21068480 | TSS1500 | Island |  |  |
|  | cg21946195 | Body | OpenSea |  |  |
|  | cg23571433 | TSS200 | Island |  |  |
|  | cg24399924 | TSS1500 | Island |  |  |
|  | cg26337070 | Body | OpenSea |  |  |
| KL | cg01308409 | Body | S_Shore | hsa-miR-30c-2-3p | 5'UTR |
|  | cg02441765 | 1stExon | Island |  |  |
|  | cg02796545 | TSS200 | Island |  |  |
|  | cg05116906 | TSS200 | Island |  |  |
|  | cg05855588 | TSS1500 | Island |  |  |
|  | cg09886946 | TSS1500 | Island |  |  |
|  | cg12162530 | Body | S_Shore |  |  |
|  | cg14145477 | TSS200 | Island |  |  |
|  | cg17106222 | TSS1500 | Island |  |  |
|  | cg17806623 | TSS1500 | Island |  |  |
|  | cg18056695 | TSS1500 | N_Shore |  |  |
|  | cg20672059 | Body | S_Shelf |  |  |
|  | cg21545902 | TSS1500 | Island |  |  |
|  | cg23132624 | TSS200 | Island |  |  |
|  | cg23282559 | 1stExon | Island |  |  |
|  | cg23584087 | Body | OpenSea |  |  |
|  | cg23943268 | TSS1500 | N_Shore |  |  |
|  | cg25698998 | TSS200 | Island |  |  |
|  | cg26325430 | 3'UTR | OpenSea |  |  |
| TNFSF12 | cg00031162 | Body | S_Shore | hsa-miR-1269a | 3'UTR |
|  | cg06479877 | TSS200 | Island |  |  |
|  | cg09648722 | 5'UTR;1stExon | Island |  |  |
|  | cg09801082 | TSS200 | Island |  |  |
|  | cg12045829 | Body | S_Shore |  |  |
|  | cg13829089 | 3'UTR | N_Shelf |  |  |
|  | cg17267493 | Body | OpenSea |  |  |
|  | cg17892169 | Body | Island |  |  |
| RSPO2 | cg00910695 | 1stExon;5'UTR | Island | hsa-miR-183-5p | 3'UTR |
|  | cg00997551 | TSS1500 | S_Shore |  |  |
|  | cg01833143 | Body | N_Shore |  |  |
|  | cg04050867 | 1stExon;5'UTR | Island |  |  |
|  | cg04549460 | TSS200 | S_Shore |  |  |
|  | cg05025239 | Body | N_Shelf |  |  |
|  | cg05742247 | Body | Island |  |  |
|  | cg06059616 | Body | N_Shore |  |  |
|  | cg07015195 | Body | N_Shore |  |  |
|  | cg07068870 | Body | N_Shore |  |  |
|  | cg07390122 | 5'UTR | Island |  |  |
|  | cg08492090 | Body | Island |  |  |
|  | cg08768048 | Body | OpenSea |  |  |
|  | cg09970569 | Body | N_Shore |  |  |
|  | cg11208967 | Body | OpenSea |  |  |
|  | cg13177747 | TSS200 | S_Shore |  |  |
|  | cg13700897 | TSS200 | S_Shore |  |  |
|  | cg14070647 | 5'UTR | Island |  |  |
|  | cg14733048 | 1stExon;5'UTR | Island |  |  |
|  | cg16845394 | TSS200 | S_Shore |  |  |
|  | cg20061155 | 5'UTR | Island |  |  |
|  | cg21088686 | Body | N_Shore |  |  |
|  | cg22600043 | TSS200 | S_Shore |  |  |
|  | cg24807106 | TSS200 | S_Shore |  |  |
| PRDM6 | cg00129563 | Body | Island | hsa-miR-127-5p | 3'UTR |
|  | cg01085125 | TSS1500 | N_Shore |  |  |
|  | cg01196322 | Body | Island |  |  |
|  | cg01360054 | Body | Island |  |  |
|  | cg01546873 | 3'UTR | OpenSea |  |  |
|  | cg02081006 | Body | N_Shore |  |  |
|  | cg02300584 | TSS1500 | N_Shore |  |  |
|  | cg02564291 | Body | N_Shore |  |  |
|  | cg03701805 | Body | OpenSea |  |  |
|  | cg03729337 | Body | N_Shore |  |  |
|  | cg03776662 | Body | Island |  |  |
|  | cg04058593 | 5'UTR;1stExon | Island |  |  |
|  | cg04503968 | TSS1500 | N_Shore |  |  |
|  | cg05272349 | Body | Island |  |  |
|  | cg05569742 | Body | N_Shore |  |  |
|  | cg05668996 | Body | Island |  |  |
|  | cg07056285 | Body | N_Shore |  |  |
|  | cg07360792 | 5'UTR | Island |  |  |
|  | cg07525144 | TSS1500 | Island |  |  |
|  | cg07622748 | 5'UTR | Island |  |  |
|  | cg07741162 | Body | N_Shore |  |  |
|  | cg07799386 | Body | Island |  |  |
|  | cg09508531 | TSS1500 | N_Shore |  |  |
|  | cg09867598 | Body | N_Shore |  |  |
|  | cg10480584 | TSS1500 | N_Shore |  |  |
|  | cg10535922 | Body | Island |  |  |
|  | cg10632209 | Body | Island |  |  |
|  | cg12018140 | Body | Island |  |  |
|  | cg13628577 | Body | Island |  |  |
|  | cg14071179 | Body | Island |  |  |
|  | cg14469693 | Body | OpenSea |  |  |
|  | cg15100227 | Body | Island |  |  |
|  | cg15193171 | TSS1500 | Island |  |  |
|  | cg15359163 | Body | N_Shore |  |  |
|  | cg16368670 | Body | OpenSea |  |  |
|  | cg17294620 | Body | OpenSea |  |  |
|  | cg17786697 | Body | N_Shore |  |  |
|  | cg18813020 | Body | Island |  |  |
|  | cg19157243 | TSS200 | N_Shore |  |  |
|  | cg19265970 | Body | Island |  |  |
|  | cg19328475 | Body | N_Shore |  |  |
|  | cg19644991 | Body | OpenSea |  |  |
|  | cg19743690 | Body | OpenSea |  |  |
|  | cg19843165 | Body | S_Shore |  |  |
|  | cg20951825 | Body | OpenSea |  |  |
|  | cg21488279 | Body | Island |  |  |
|  | cg21523871 | Body | Island |  |  |
|  | cg23289079 | Body | N_Shore |  |  |
|  | cg24806571 | Body | S_Shelf |  |  |
|  | cg25116709 | Body | Island |  |  |
|  | cg26244952 | TSS1500 | Island |  |  |
|  | cg26416905 | Body | OpenSea |  |  |
|  | cg26938014 | Body | N_Shore |  |  |
|  | cg27065003 | Body | N_Shore |  |  |
|  | cg27083484 | Body | OpenSea |  |  |
|  | cg27087956 | Body | N_Shore |  |  |
| TRPC4 | cg01764020 | TSS200 | Island | hsa-miR-127-5p | 3'UTR |
|  | cg02057391 | 1stExon;5'UTR | Island |  |  |
|  | cg06710083 | Body | OpenSea |  |  |
|  | cg13549719 | TSS200 | Island |  |  |
|  | cg15071251 | 5'UTR | N_Shore |  |  |
|  | cg15398976 | TSS1500 | S_Shore |  |  |
|  | cg15696906 | TSS200 | Island |  |  |
|  | cg16409955 | TSS1500 | Island |  |  |
|  | cg17071063 | TSS1500 | S_Shore |  |  |
|  | cg17979426 | Body | OpenSea |  |  |
|  | cg18182438 | Body | OpenSea |  |  |
|  | cg19275632 | TSS200 | Island |  |  |
|  | cg20535787 | TSS200 | Island |  |  |
|  | cg21081971 | TSS1500 | S_Shore |  |  |
|  | cg21432954 | 1stExon;5'UTR | Island |  |  |
|  | cg23021771 | TSS1500 | S_Shore |  |  |
|  | cg23307680 | 5'UTR | N_Shore |  |  |
| ART4 | cg00253681 | TSS200 | OpenSea | hsa-miR-1269a | 3'UTR |
|  | cg04228042 | TSS200 | OpenSea |  |  |
|  | cg08905654 | TSS1500 | OpenSea |  |  |
|  | cg10047173 | TSS200 | OpenSea |  |  |
|  | cg13573513 | 5'UTR;1stExon | OpenSea |  |  |
|  | cg19532939 | TSS200 | OpenSea |  |  |
|  | cg20967028 | 5'UTR;1stExon | OpenSea |  |  |
|  | cg26614815 | TSS1500 | OpenSea |  |  |
| RIMS4 | cg01304575 | 3'UTR | S_Shore | hsa-miR-1269a | 3'UTR |
|  | cg05139523 | Body | N_Shelf |  |  |
|  | cg12343182 | TSS1500 | Island |  |  |
|  | cg15207742 | Body | Island |  |  |
|  | cg20498414 | TSS1500 | Island |  |  |
|  | cg20752831 | 3'UTR | S_Shelf |  |  |
|  | cg21500133 | TSS1500 | Island |  |  |
|  | cg27361134 | TSS1500 | Island |  |  |
| PROK2 | cg01025398 | Body | N_Shore | hsa-miR-30a-3p | 5'UTR |
|  | cg01904145 | Body | N_Shelf |  |  |
|  | cg05515792 | Body | N_Shore |  |  |
|  | cg08555612 | TSS1500 | Island |  |  |
|  | cg09655952 | Body | N_Shore |  |  |
|  | cg14981807 | Body | N_Shore |  |  |
|  | cg15798455 | Body | N_Shore |  |  |
|  | cg19388776 | TSS1500 | S_Shore |  |  |
|  | cg19822518 | TSS1500 | S_Shore |  |  |
|  | cg22264409 | Body | OpenSea |  |  |
|  | cg22799510 | 3'UTR | OpenSea |  |  |
|  | cg23039279 | 5'UTR;1stExon | Island |  |  |
| HIF3A | cg01552731 | 5'UTR;Body;1stExon | N_Shore | hsa-miR-142-3p | 3'UTR |
|  | cg02879662 | TSS1500;Body | Island |  |  |
|  | cg05286653 | TSS1500;Body | Island |  |  |
|  | cg07684068 | Body | S_Shore |  |  |
|  | cg09789590 | TSS1500;Body | Island |  |  |
|  | cg12068280 | 5'UTR;Body | N_Shelf |  |  |
|  | cg14088357 | TSS1500;1stExon | Island |  |  |
|  | cg14117138 | TSS1500 | Island |  |  |
|  | cg14153927 | 3'UTR | N_Shelf |  |  |
|  | cg15229275 | TSS1500 | Island |  |  |
|  | cg16672562 | 5'UTR;Body;1stExon | S_Shore |  |  |
|  | cg19045239 | TSS1500 | N_Shore |  |  |
|  | cg19310908 | TSS1500 | Island |  |  |
|  | cg20667364 | Body | Island |  |  |
|  | cg21617218 | TSS1500;5'UTR;Body | N_Shore |  |  |
|  | cg22891070 | TSS200;Body | S_Shore |  |  |
|  | cg23548163 | 5'UTR;Body | Island |  |  |
|  | cg25196389 | 5'UTR;Body | Island |  |  |
|  | cg25460031 | 3'UTR | OpenSea |  |  |
|  | cg26749414 | 5'UTR;Body | Island |  |  |
|  | cg27146050 | TSS200;Body | S_Shore |  |  |
| SLC2A3 | cg02511315 | TSS1500 | OpenSea | hsa-miR-486-5p | 5'UTR |
|  | cg02591487 | 5'UTR;1stExon | OpenSea |  |  |
|  | cg10338338 | TSS200 | OpenSea |  |  |
|  | cg20313963 | TSS1500 | OpenSea |  |  |
|  | cg20972214 | 5'UTR;1stExon | OpenSea |  |  |
|  | cg25580254 | TSS200 | OpenSea |  |  |
| P2RY1 | cg00504134 | TSS1500 | N_Shore | hsa-miR-767-5p | 3'UTR |
|  | cg02841941 | TSS200 | Island |  |  |
|  | cg03293976 | 1stExon | S_Shore |  |  |
|  | cg04845053 | 5'UTR;1stExon | Island |  |  |
|  | cg05086811 | TSS200 | Island |  |  |
|  | cg05488043 | 5'UTR;1stExon | Island |  |  |
|  | cg06809364 | 1stExon;3'UTR | S_Shore |  |  |
|  | cg06903384 | 1stExon | Island |  |  |
|  | cg08570275 | 5'UTR;1stExon | Island |  |  |
|  | cg10949611 | TSS200 | N_Shore |  |  |
|  | cg11161961 | 1stExon | S_Shore |  |  |
|  | cg14080015 | 5'UTR;1stExon | Island |  |  |
|  | cg14921522 | 1stExon | S_Shore |  |  |
|  | cg20944305 | 1stExon | Island |  |  |
|  | cg21143560 | TSS1500 | N_Shore |  |  |
|  | cg22055427 | TSS1500 | N_Shore |  |  |
|  | cg26125811 | TSS200 | N_Shore |  |  |
| SMAD7 | cg00145587 | Body | Island | hsa-miR-105-5p | 3'UTR |
|  | cg00214056 | TSS1500 | Island |  |  |
|  | cg00839844 | Body | N_Shore |  |  |
|  | cg02184281 | Body | Island |  |  |
|  | cg05119084 | TSS200 | Island |  |  |
|  | cg05310557 | TSS200 | Island |  |  |
|  | cg06203649 | 3'UTR | N_Shore |  |  |
|  | cg07661480 | 3'UTR | Island |  |  |
|  | cg11023721 | TSS1500 | Island |  |  |
|  | cg11645306 | Body | N_Shore |  |  |
|  | cg11909137 | Body | OpenSea |  |  |
|  | cg12651664 | TSS200 | Island |  |  |
|  | cg13398027 | Body | Island |  |  |
|  | cg13967780 | 1stExon;5'UTR | Island |  |  |
|  | cg14283454 | Body | S_Shore |  |  |
|  | cg14751914 | TSS1500 | Island |  |  |
|  | cg15153018 | TSS1500 | Island |  |  |
|  | cg15166561 | TSS200 | Island |  |  |
|  | cg19430553 | Body | OpenSea |  |  |
|  | cg19575813 | 1stExon;5'UTR | Island |  |  |
|  | cg20800216 | Body | N_Shelf |  |  |
|  | cg21505940 | TSS200 | Island |  |  |
|  | cg21718735 | Body | N_Shore |  |  |
|  | cg23699700 | Body | Island |  |  |
|  | cg24375218 | Body | OpenSea |  |  |
|  | cg25986322 | Body | Island |  |  |
| PTPRT | cg00538248 | Body | N_Shore | hsa-miR-105-5p | 3'UTR |
|  | cg03403065 | 1stExon | Island |  |  |
|  | cg04541293 | TSS1500 | Island |  |  |
|  | cg07167168 | TSS1500 | Island |  |  |
|  | cg08413157 | TSS200 | Island |  |  |
|  | cg09309085 | 3'UTR | OpenSea |  |  |
|  | cg13168820 | 1stExon | Island |  |  |
|  | cg17859110 | TSS1500 | Island |  |  |
|  | cg21672843 | TSS1500 | Island |  |  |
|  | cg23357198 | Body | Island |  |  |
|  | cg25649000 | Body | Island |  |  |
|  | cg26105214 | Body | Island |  |  |
| CCDC68 | cg03778029 | TSS200;TSS1500 | Island | hsa-miR-127-5p | 3'UTR |
|  | cg07058998 | TSS200;5'UTR | N_Shore |  |  |
|  | cg07177852 | 5'UTR;TSS1500 | N_Shore |  |  |
|  | cg12113740 | TSS200;5'UTR | N_Shore |  |  |
|  | cg12955441 | 5'UTR;TSS1500 | Island |  |  |
|  | cg12974637 | 1stExon;5'UTR;TSS1500 | Island |  |  |
|  | cg16583884 | TSS200;5'UTR | N_Shore |  |  |
|  | cg26630735 | TSS200 | Island |  |  |
| UCP1 | cg01109023 | Body | N_Shelf | hsa-miR-30c-2-3p | 5'UTR |
|  | cg01283834 | Body | N_Shore |  |  |
|  | cg02268229 | TSS200 | Island |  |  |
|  | cg02759110 | 1stExon | N_Shore |  |  |
|  | cg05644921 | Body | N_Shore |  |  |
|  | cg09181644 | TSS1500 | S_Shore |  |  |
|  | cg14078662 | TSS1500 | S_Shore |  |  |
|  | cg14223995 | 1stExon | N_Shore |  |  |
|  | cg16512615 | TSS1500 | Island |  |  |
|  | cg19346645 | TSS200 | Island |  |  |
|  | cg21325154 | TSS1500 | Island |  |  |
|  | cg25599538 | TSS200 | Island |  |  |
|  | cg25793521 | TSS200 | Island |  |  |
|  | cg25797055 | TSS200 | Island |  |  |
|  | cg26004759 | Body | N_Shore |  |  |
| GABRA4 | cg01064749 | Body | N_Shore | hsa-miR-127-5p | 3'UTR |
|  | cg03593419 | 5'UTR;1stExon | Island |  |  |
|  | cg09812070 | TSS200 | Island |  |  |
|  | cg16358826 | TSS1500 | S_Shore |  |  |
|  | cg16976370 | TSS1500 | Island |  |  |
|  | cg17491947 | Body | N_Shore |  |  |
|  | cg18328702 | Body | N_Shelf |  |  |
|  | cg18909973 | TSS1500 | S_Shore |  |  |
|  | cg22345214 | 5'UTR;1stExon | Island |  |  |
|  | cg22785561 | 3'UTR | OpenSea |  |  |
|  | cg22798201 | TSS200 | Island |  |  |
|  | cg24154839 | TSS200 | Island |  |  |
|  | cg24394172 | Body | Island |  |  |
|  | cg25951981 | TSS200 | Island |  |  |
| FAM124B | cg00698413 | 5'UTR;1stExon | OpenSea | hsa-miR-1307-5p | 3'UTR |
|  | cg01244015 | TSS200 | OpenSea |  |  |
|  | cg01833675 | TSS200 | OpenSea |  |  |
|  | cg10114725 | TSS200 | OpenSea |  |  |
|  | cg16116321 | Body;3'UTR | OpenSea |  |  |
|  | cg18890556 | 5'UTR;1stExon | OpenSea |  |  |
|  | cg19108747 | 1stExon | OpenSea |  |  |
|  | cg23013850 | 1stExon | OpenSea |  |  |
|  | cg24516901 | TSS200 | OpenSea |  |  |
|  | cg25313930 | TSS200 | OpenSea |  |  |
|  | cg25717464 | 1stExon | OpenSea |  |  |
|  | cg26154999 | 1stExon | OpenSea |  |  |
| USHBP1 | cg04459545 | TSS200 | OpenSea | hsa-miR-196a-5p | 3'UTR |
|  | cg06910100 | Body | OpenSea |  |  |
|  | cg12517843 | Body | S_Shelf |  |  |
|  | cg24766041 | 5'UTR | OpenSea |  |  |
| CCND2 | cg00417288 | Body | OpenSea | hsa-miR-105-5p | 3'UTR |
|  | cg00814733 | TSS1500 | Island |  |  |
|  | cg00888007 | TSS1500 | Island |  |  |
|  | cg01008117 | Body | OpenSea |  |  |
|  | cg01093885 | Body | S_Shore |  |  |
|  | cg01264332 | Body | OpenSea |  |  |
|  | cg02401639 | Body | OpenSea |  |  |
|  | cg02765328 | 1stExon | Island |  |  |
|  | cg03801902 | TSS1500 | Island |  |  |
|  | cg05153364 | TSS1500 | Island |  |  |
|  | cg05667158 | TSS1500 | Island |  |  |
|  | cg05987650 | TSS1500 | Island |  |  |
|  | cg07066369 | Body | S_Shore |  |  |
|  | cg07181862 | Body | OpenSea |  |  |
|  | cg07297830 | Body | OpenSea |  |  |
|  | cg08069899 | Body | Island |  |  |
|  | cg08553284 | TSS1500 | Island |  |  |
|  | cg09277744 | Body | S_Shore |  |  |
|  | cg09799980 | Body | OpenSea |  |  |
|  | cg10091994 | TSS1500 | Island |  |  |
|  | cg11135021 | TSS1500 | Island |  |  |
|  | cg12105382 | Body | OpenSea |  |  |
|  | cg12382902 | Body | Island |  |  |
|  | cg12594237 | Body | OpenSea |  |  |
|  | cg13080151 | TSS1500 | Island |  |  |
|  | cg13391820 | Body | Island |  |  |
|  | cg13404421 | TSS1500 | Island |  |  |
|  | cg13575161 | TSS1500 | Island |  |  |
|  | cg13618596 | Body | S_Shore |  |  |
|  | cg13801381 | Body | Island |  |  |
|  | cg14414332 | Body | OpenSea |  |  |
|  | cg14782437 | TSS1500 | Island |  |  |
|  | cg14834893 | Body | OpenSea |  |  |
|  | cg14914982 | 5'UTR;1stExon | N_Shore |  |  |
|  | cg15249639 | TSS1500 | Island |  |  |
|  | cg15264083 | 5'UTR;1stExon | N_Shore |  |  |
|  | cg15348972 | 5'UTR;1stExon | N_Shore |  |  |
|  | cg15993083 | TSS1500 | Island |  |  |
|  | cg16310717 | Body | Island |  |  |
|  | cg16994506 | Body | Island |  |  |
|  | cg17296482 | TSS1500 | Island |  |  |
|  | cg17558623 | Body | OpenSea |  |  |
|  | cg17580045 | Body | S_Shore |  |  |
|  | cg17886028 | Body | Island |  |  |
|  | cg18566594 | TSS1500 | Island |  |  |
|  | cg18584387 | Body | S_Shore |  |  |
|  | cg19582614 | TSS1500 | Island |  |  |
|  | cg21057429 | TSS1500 | N_Shore |  |  |
|  | cg21177064 | 3'UTR | OpenSea |  |  |
|  | cg21462428 | TSS1500 | Island |  |  |
|  | cg22500428 | TSS1500 | Island |  |  |
|  | cg22555262 | TSS1500 | N_Shore |  |  |
|  | cg22678952 | TSS1500 | Island |  |  |
|  | cg22794990 | 3'UTR | OpenSea |  |  |
|  | cg23235334 | TSS1500 | Island |  |  |
|  | cg24018760 | TSS1500 | Island |  |  |
|  | cg24626079 | TSS1500 | Island |  |  |
|  | cg25126456 | TSS1500 | Island |  |  |
|  | cg25425078 | TSS1500 | Island |  |  |
|  | cg25454116 | TSS1500 | Island |  |  |
|  | cg26377281 | Body | OpenSea |  |  |
|  | cg26551299 | Body | S_Shelf |  |  |
|  | cg26864834 | TSS1500 | N_Shore |  |  |
|  | cg26989531 | 5'UTR;1stExon | N_Shore |  |  |
|  | cg27317813 | Body | OpenSea |  |  |
| EYA4 | cg01162672 | TSS1500 | Island | hsa-miR-1269a | 3'UTR |
|  | cg01328892 | TSS200 | Island |  |  |
|  | cg01401376 | 5'UTR | Island |  |  |
|  | cg01805282 | 5'UTR | Island |  |  |
|  | cg01957732 | TSS1500 | N_Shore |  |  |
|  | cg02446647 | TSS1500 | Island |  |  |
|  | cg04548096 | Body | OpenSea |  |  |
|  | cg05062333 | TSS1500 | Island |  |  |
|  | cg05085230 | TSS200 | Island |  |  |
|  | cg05493394 | TSS1500 | N_Shore |  |  |
|  | cg06132028 | TSS200 | Island |  |  |
|  | cg06181518 | TSS1500 | Island |  |  |
|  | cg06393563 | 5'UTR | S_Shore |  |  |
|  | cg06525347 | TSS1500 | N_Shore |  |  |
|  | cg06764333 | 5'UTR | S_Shore |  |  |
|  | cg07224291 | Body | OpenSea |  |  |
|  | cg07327468 | TSS1500 | N_Shore |  |  |
|  | cg07817705 | 3'UTR | OpenSea |  |  |
|  | cg08712932 | TSS200 | Island |  |  |
|  | cg08917489 | 5'UTR | S_Shore |  |  |
|  | cg09649901 | 3'UTR | OpenSea |  |  |
|  | cg10105884 | Body | OpenSea |  |  |
|  | cg10675276 | TSS1500 | N_Shore |  |  |
|  | cg10782703 | 3'UTR | OpenSea |  |  |
|  | cg11518846 | TSS1500 | Island |  |  |
|  | cg11664500 | TSS200 | Island |  |  |
|  | cg11942956 | TSS200 | Island |  |  |
|  | cg12152919 | TSS1500 | N_Shore |  |  |
|  | cg13283932 | Body | OpenSea |  |  |
|  | cg14017655 | TSS1500 | Island |  |  |
|  | cg14270292 | 1stExon;5'UTR | Island |  |  |
|  | cg14287112 | TSS200 | Island |  |  |
|  | cg14343214 | TSS1500 | N_Shore |  |  |
|  | cg14711475 | Body | OpenSea |  |  |
|  | cg14817541 | 5'UTR | Island |  |  |
|  | cg14866547 | Body | OpenSea |  |  |
|  | cg15246805 | 5'UTR | OpenSea |  |  |
|  | cg15729404 | TSS1500 | N_Shore |  |  |
|  | cg15865026 | Body | OpenSea |  |  |
|  | cg16347317 | TSS200 | Island |  |  |
|  | cg17722675 | 5'UTR | Island |  |  |
|  | cg17736819 | Body | OpenSea |  |  |
|  | cg17838029 | TSS200 | Island |  |  |
|  | cg18625409 | Body | OpenSea |  |  |
|  | cg18714224 | 5'UTR | S_Shore |  |  |
|  | cg20286200 | TSS1500 | Island |  |  |
|  | cg20330472 | TSS1500 | N_Shore |  |  |
|  | cg20611276 | TSS1500 | N_Shore |  |  |
|  | cg20787173 | TSS200 | Island |  |  |
|  | cg20980055 | TSS1500 | Island |  |  |
|  | cg21296676 | TSS1500 | N_Shore |  |  |
|  | cg21390624 | TSS1500 | N_Shore |  |  |
|  | cg21607030 | 5'UTR | Island |  |  |
|  | cg22462983 | TSS1500 | N_Shore |  |  |
|  | cg22871668 | TSS200 | Island |  |  |
|  | cg23028941 | Body | OpenSea |  |  |
|  | cg24176563 | 1stExon;5'UTR | Island |  |  |
|  | cg24675448 | 5'UTR | Island |  |  |
|  | cg24842760 | TSS1500 | N_Shore |  |  |
|  | cg25075347 | Body | OpenSea |  |  |
|  | cg25375297 | Body | OpenSea |  |  |
|  | cg26449787 | TSS1500 | Island |  |  |
|  | cg26501369 | TSS1500 | N_Shore |  |  |
|  | cg26656135 | 1stExon;5'UTR | Island |  |  |
| LEP | cg00666422 | 5'UTR | S_Shore | hsa-miR-1269b | 3'UTR |
|  | cg00840332 | TSS200 | Island |  |  |
|  | cg03084214 | TSS1500 | N_Shore |  |  |
|  | cg07464571 | TSS1500 | Island |  |  |
|  | cg12083122 | 5'UTR | S_Shelf |  |  |
|  | cg12782180 | TSS1500 | Island |  |  |
|  | cg13381984 | 5'UTR;1stExon | Island |  |  |
|  | cg16683741 | 5'UTR | OpenSea |  |  |
|  | cg19594666 | TSS200 | Island |  |  |
|  | cg24862443 | 3'UTR | OpenSea |  |  |
|  | cg25435800 | 5'UTR | OpenSea |  |  |
|  | cg26814075 | TSS200 | Island |  |  |
| TMEM255A | cg04069374 | Body | Island | hsa-miR-105-5p | 3'UTR |
|  | cg04455999 | Body | N_Shore |  |  |
|  | cg05185504 | Body | N_Shore |  |  |
|  | cg05789980 | Body | N_Shelf |  |  |
|  | cg08348649 | Body | N_Shore |  |  |
|  | cg08760398 | Body | N_Shore |  |  |
|  | cg09793609 | TSS200 | S_Shore |  |  |
|  | cg13204538 | 1stExon;5'UTR | S_Shore |  |  |
|  | cg16515238 | Body | Island |  |  |
|  | cg16692973 | 1stExon;5'UTR | Island |  |  |
|  | cg17562247 | Body | N_Shore |  |  |
|  | cg18291437 | TSS1500 | S_Shore |  |  |
|  | cg18456549 | 3'UTR | OpenSea |  |  |
|  | cg19930056 | Body | N_Shore |  |  |
|  | cg21329507 | TSS200 | S_Shore |  |  |
|  | cg21480420 | TSS200 | S_Shore |  |  |
|  | cg23483251 | 1stExon;5'UTR | S_Shore |  |  |
|  | cg26583344 | Body | N_Shore |  |  |
| RIC3 | cg03054541 | 3'UTR | OpenSea | hsa-miR-127-5p | 3'UTR |
|  | cg03707776 | 1stExon | Island |  |  |
|  | cg08383315 | 5'UTR;1stExon | Island |  |  |
|  | cg13788027 | Body | Island |  |  |
|  | cg16484162 | Body | OpenSea |  |  |
|  | cg17603132 | Body | N_Shore |  |  |
|  | cg25098208 | TSS200 | Island |  |  |
|  | cg25778535 | 5'UTR;1stExon | Island |  |  |
| WIF1 | cg03509412 | 5'UTR;1stExon | Island | hsa-miR-490-5p | 5'UTR |
|  | cg10065957 | Body | N_Shore |  |  |
|  | cg15862358 | TSS1500 | S_Shore |  |  |
|  | cg16009877 | 3'UTR | OpenSea |  |  |
|  | cg16099107 | Body | N_Shore |  |  |
|  | cg19427610 | 5'UTR;1stExon | Island |  |  |
|  | cg20098478 | TSS1500 | Island |  |  |
|  | cg21383810 | TSS1500 | Island |  |  |
|  | cg24166864 | TSS200 | Island |  |  |
|  | cg26397188 | TSS200 | Island |  |  |
|  | cg26733786 | 5'UTR;1stExon | Island |  |  |
| NRN1 | cg00565348 | Body | Island | hsa-miR-210-3p | 3'UTR |
|  | cg01410163 | Body | Island |  |  |
|  | cg01493727 | Body | N_Shore |  |  |
|  | cg01566449 | 1stExon;5'UTR | N_Shore |  |  |
|  | cg01807407 | Body | Island |  |  |
|  | cg01928807 | Body | N_Shore |  |  |
|  | cg03707308 | Body | Island |  |  |
|  | cg03759454 | Body | S_Shore |  |  |
|  | cg04187403 | Body | Island |  |  |
|  | cg04475375 | TSS1500 | N_Shore |  |  |
|  | cg05265020 | Body | Island |  |  |
|  | cg06495131 | Body | Island |  |  |
|  | cg07593839 | TSS200 | Island |  |  |
|  | cg07878800 | Body | Island |  |  |
|  | cg07994987 | TSS200 | Island |  |  |
|  | cg08545493 | Body | Island |  |  |
|  | cg08649201 | Body | Island |  |  |
|  | cg08827756 | Body | Island |  |  |
|  | cg09555118 | Body | Island |  |  |
|  | cg09986316 | Body | N_Shore |  |  |
|  | cg10223827 | 1stExon;5'UTR | Island |  |  |
|  | cg10304637 | Body | Island |  |  |
|  | cg11504897 | 1stExon;5'UTR | Island |  |  |
|  | cg11564981 | TSS200 | Island |  |  |
|  | cg12595585 | TSS1500 | Island |  |  |
|  | cg13021333 | Body | Island |  |  |
|  | cg13773570 | Body | Island |  |  |
|  | cg13858611 | Body | N_Shore |  |  |
|  | cg14386951 | 3'UTR | Island |  |  |
|  | cg16648632 | Body | Island |  |  |
|  | cg17658634 | Body | Island |  |  |
|  | cg18924298 | Body | Island |  |  |
|  | cg20223728 | Body | N_Shore |  |  |
|  | cg20904489 | Body | N_Shore |  |  |
|  | cg22142858 | Body | Island |  |  |
|  | cg24720571 | Body | Island |  |  |
|  | cg25307691 | TSS1500 | Island |  |  |
|  | cg25511429 | TSS1500 | N_Shore |  |  |
|  | cg25829425 | Body | Island |  |  |
|  | cg27363741 | Body | N_Shore |  |  |
| GIPC2 | cg00347563 | 1stExon;5'UTR | Island | hsa-miR-127-5p | 3'UTR |
|  | cg01074657 | TSS200 | N_Shore |  |  |
|  | cg04912843 | 1stExon | Island |  |  |
|  | cg09107315 | TSS200 | N_Shore |  |  |
|  | cg09662920 | TSS200 | N_Shore |  |  |
|  | cg09826056 | TSS1500 | N_Shore |  |  |
|  | cg19766489 | TSS1500 | N_Shore |  |  |
|  | cg24496666 | 1stExon | Island |  |  |
|  | cg24719487 | Body | Island |  |  |
|  | cg25288420 | 1stExon;5'UTR | Island |  |  |
| TBX3 | cg00347620 | Body | Island | hsa-miR-210-3p | 3'UTR |
|  | cg00499475 | TSS1500 | Island |  |  |
|  | cg00517511 | Body | S_Shelf |  |  |
|  | cg01078446 | Body | S_Shore |  |  |
|  | cg02323277 | 3'UTR | Island |  |  |
|  | cg06211872 | TSS1500 | N_Shore |  |  |
|  | cg08330778 | TSS200 | Island |  |  |
|  | cg09053536 | Body | N_Shelf |  |  |
|  | cg09413529 | Body | Island |  |  |
|  | cg10330024 | 1stExon;5'UTR | Island |  |  |
|  | cg10530281 | TSS200 | Island |  |  |
|  | cg11246938 | Body | Island |  |  |
|  | cg12530050 | 1stExon;5'UTR | Island |  |  |
|  | cg13030332 | Body | N_Shelf |  |  |
|  | cg13829104 | Body | N_Shore |  |  |
|  | cg13930596 | 3'UTR | N_Shore |  |  |
|  | cg16277169 | Body | Island |  |  |
|  | cg16406892 | Body | N_Shore |  |  |
|  | cg18161956 | Body | Island |  |  |
|  | cg18575770 | 1stExon;5'UTR | Island |  |  |
|  | cg18673726 | TSS200 | Island |  |  |
|  | cg19303619 | 1stExon;5'UTR | Island |  |  |
|  | cg19346371 | 1stExon | Island |  |  |
|  | cg19713038 | 1stExon;5'UTR | Island |  |  |
|  | cg21913681 | TSS200 | Island |  |  |
|  | cg22635491 | Body | Island |  |  |
|  | cg22797735 | TSS200 | Island |  |  |
|  | cg25058261 | 3'UTR | N_Shore |  |  |
|  | cg27630311 | 1stExon | Island |  |  |
| GPC3 | cg02442693 | Body | Island | hsa-miR-196a-5p | 3'UTR |
|  | cg04139820 | TSS1500 | S_Shore |  |  |
|  | cg05445331 | Body | Island |  |  |
|  | cg06384623 | 1stExon | Island |  |  |
|  | cg06431415 | Body | N_Shelf |  |  |
|  | cg07297906 | 5'UTR;1stExon | Island |  |  |
|  | cg09760728 | Body | Island |  |  |
|  | cg11442732 | Body | Island |  |  |
|  | cg14867863 | TSS1500 | Island |  |  |
|  | cg16272791 | TSS1500 | Island |  |  |
|  | cg17725109 | Body | OpenSea |  |  |
|  | cg27496708 | 5'UTR;1stExon | Island |  |  |
| FOXF2 | cg02386403 | 1stExon | Island | hsa-miR-182-5p | 3'UTR |
|  | cg02490736 | Body | N_Shore |  |  |
|  | cg02547269 | 1stExon | Island |  |  |
|  | cg03848675 | TSS1500 | Island |  |  |
|  | cg04094811 | Body | Island |  |  |
|  | cg04187121 | TSS1500 | N_Shore |  |  |
|  | cg05679108 | Body | S_Shore |  |  |
|  | cg06005891 | TSS1500 | Island |  |  |
|  | cg09292226 | Body | N_Shore |  |  |
|  | cg09491120 | Body | S_Shore |  |  |
|  | cg10759602 | Body | Island |  |  |
|  | cg12221475 | 1stExon | Island |  |  |
|  | cg12611423 | TSS200 | Island |  |  |
|  | cg12835736 | 3'UTR | S_Shore |  |  |
|  | cg15141195 | Body | S_Shore |  |  |
|  | cg16619978 | TSS1500 | N_Shore |  |  |
|  | cg16935061 | 3'UTR | S_Shore |  |  |
|  | cg16945312 | Body | N_Shore |  |  |
|  | cg17305266 | 1stExon | Island |  |  |
|  | cg19519310 | TSS1500 | Island |  |  |
|  | cg20457275 | Body | Island |  |  |
|  | cg24151352 | 1stExon | Island |  |  |
|  | cg26174583 | Body | N_Shore |  |  |
|  | cg26987597 | 1stExon | Island |  |  |
| PTCHD1 | cg06619487 | 1stExon | Island | hsa-miR-1269a | 3'UTR |
|  | cg10511972 | TSS1500 | Island |  |  |
|  | cg10549828 | TSS1500 | Island |  |  |
|  | cg10674704 | Body | Island |  |  |
|  | cg17227235 | 3'UTR | OpenSea |  |  |
|  | cg18005219 | Body | Island |  |  |
|  | cg20879096 | TSS1500 | Island |  |  |
|  | cg21657265 | TSS200 | Island |  |  |
|  | cg22691634 | Body | S_Shelf |  |  |
|  | cg26912861 | Body | S_Shore |  |  |
|  | cg27609596 | TSS1500 | Island |  |  |
| AMER2 | cg00313750 | TSS1500 | S_Shore | hsa-miR-1307-5p | 3'UTR |
|  | cg03369269 | TSS200 | Island |  |  |
|  | cg04323814 | TSS1500 | S_Shore |  |  |
|  | cg05438320 | TSS1500 | Island |  |  |
|  | cg06779705 | TSS1500 | S_Shore |  |  |
|  | cg07348641 | TSS1500 | S_Shore |  |  |
|  | cg07920503 | 1stExon | Island |  |  |
|  | cg11030811 | 3'UTR;1stExon | N_Shore |  |  |
|  | cg13055385 | Body;1stExon | Island |  |  |
|  | cg14880499 | 1stExon;5'UTR | Island |  |  |
|  | cg17205928 | TSS1500 | S_Shore |  |  |
|  | cg18034232 | TSS200 | Island |  |  |
|  | cg18815647 | Body;1stExon | Island |  |  |
|  | cg21052415 | TSS1500 | S_Shore |  |  |
|  | cg22029275 | 1stExon;5'UTR | Island |  |  |
|  | cg22141111 | TSS200 | Island |  |  |
|  | cg24296626 | TSS200 | Island |  |  |
| ST6GALNAC5 | cg00294096 | Body | Island | hsa-miR-127-5p | 3'UTR |
|  | cg02639927 | Body | S_Shore |  |  |
|  | cg03761543 | Body | OpenSea |  |  |
|  | cg04077662 | 5'UTR;1stExon | Island |  |  |
|  | cg06201642 | 5'UTR;1stExon | Island |  |  |
|  | cg07499159 | Body | OpenSea |  |  |
|  | cg08431563 | Body | Island |  |  |
|  | cg09511846 | TSS200 | N_Shore |  |  |
|  | cg11338128 | Body | OpenSea |  |  |
|  | cg13463054 | TSS200 | Island |  |  |
|  | cg13823136 | TSS200 | Island |  |  |
|  | cg15100100 | TSS200 | N_Shore |  |  |
|  | cg16926302 | Body | OpenSea |  |  |
|  | cg16966815 | TSS200 | Island |  |  |
|  | cg18380175 | Body | Island |  |  |
|  | cg20417424 | TSS1500 | N_Shore |  |  |
|  | cg21604970 | Body | OpenSea |  |  |
|  | cg23243867 | Body | Island |  |  |
|  | cg24528350 | Body | OpenSea |  |  |
|  | cg25272065 | Body | S_Shore |  |  |
|  | cg25306611 | Body | Island |  |  |
|  | cg27519691 | TSS200 | N_Shore |  |  |
| TBX5 | cg00182639 | Body;5'UTR;1stExon | N_Shore | hsa-miR-139-3p | 5'UTR |
|  | cg00590029 | Body | Island |  |  |
|  | cg00642359 | TSS200;5'UTR | N_Shore |  |  |
|  | cg00756451 | 5'UTR;TSS1500 | N_Shore |  |  |
|  | cg02011607 | 3'UTR | OpenSea |  |  |
|  | cg02712224 | Body | Island |  |  |
|  | cg03843000 | TSS1500 | Island |  |  |
|  | cg03877376 | 1stExon;5'UTR | Island |  |  |
|  | cg04002444 | 1stExon;5'UTR | Island |  |  |
|  | cg04562909 | Body | S_Shore |  |  |
|  | cg04685570 | Body;5'UTR | N_Shore |  |  |
|  | cg05065572 | Body | Island |  |  |
|  | cg05555207 | TSS1500 | Island |  |  |
|  | cg05769349 | TSS1500 | Island |  |  |
|  | cg05929882 | TSS1500 | Island |  |  |
|  | cg06717565 | Body | N_Shore |  |  |
|  | cg06725552 | Body | N_Shore |  |  |
|  | cg06875424 | Body | S_Shore |  |  |
|  | cg06911121 | TSS1500 | Island |  |  |
|  | cg07658357 | Body | N_Shore |  |  |
|  | cg08318726 | 5'UTR;TSS1500 | N_Shore |  |  |
|  | cg08807097 | TSS200;5'UTR | N_Shore |  |  |
|  | cg08900101 | Body | N_Shore |  |  |
|  | cg08985029 | 5'UTR | Island |  |  |
|  | cg09042277 | Body;5'UTR | Island |  |  |
|  | cg09233651 | Body | Island |  |  |
|  | cg10281002 | TSS200 | Island |  |  |
|  | cg11357746 | Body | N_Shore |  |  |
|  | cg11717507 | Body | Island |  |  |
|  | cg11841394 | Body;5'UTR | N_Shore |  |  |
|  | cg12610207 | Body | Island |  |  |
|  | cg12670347 | TSS1500 | Island |  |  |
|  | cg14126466 | Body | N_Shelf |  |  |
|  | cg14214262 | 5'UTR;TSS1500 | Island |  |  |
|  | cg14264795 | 5'UTR;TSS1500 | N_Shore |  |  |
|  | cg14431443 | TSS200 | Island |  |  |
|  | cg16249035 | 1stExon;5'UTR | Island |  |  |
|  | cg16315058 | 1stExon;5'UTR | S_Shore |  |  |
|  | cg16458436 | TSS200 | Island |  |  |
|  | cg16559598 | TSS1500 | Island |  |  |
|  | cg16605327 | TSS1500 | Island |  |  |
|  | cg16805360 | 5'UTR;TSS1500 | N_Shore |  |  |
|  | cg17462200 | 5'UTR;TSS1500 | N_Shore |  |  |
|  | cg17554126 | Body | N_Shore |  |  |
|  | cg17645823 | TSS200 | Island |  |  |
|  | cg18173058 | Body | Island |  |  |
|  | cg18689332 | Body | N_Shore |  |  |
|  | cg18692678 | Body | Island |  |  |
|  | cg19290410 | TSS1500 | Island |  |  |
|  | cg19746940 | Body | N_Shore |  |  |
|  | cg19787532 | TSS200;5'UTR | N_Shore |  |  |
|  | cg20099830 | 5'UTR;TSS1500 | N_Shore |  |  |
|  | cg20426571 | TSS200;5'UTR | N_Shore |  |  |
|  | cg20875159 | 1stExon;5'UTR | S_Shore |  |  |
|  | cg21611810 | TSS200;5'UTR | N_Shore |  |  |
|  | cg21907579 | 1stExon;5'UTR | Island |  |  |
|  | cg22045225 | TSS200;5'UTR | N_Shore |  |  |
|  | cg22628281 | Body | N_Shore |  |  |
|  | cg23320862 | TSS200;5'UTR | N_Shore |  |  |
|  | cg23820885 | Body;5'UTR | Island |  |  |
|  | cg23827572 | TSS200 | Island |  |  |
|  | cg25079102 | 1stExon;5'UTR | Island |  |  |
|  | cg25399352 | Body | N_Shore |  |  |
|  | cg25556579 | Body | OpenSea |  |  |
|  | cg26196480 | TSS200 | Island |  |  |
| RBM24 | cg00381411 | TSS1500;Body | Island | hsa-miR-1307-5p | 3'UTR |
|  | cg02066331 | Body;5'UTR;TSS200 | S_Shore |  |  |
|  | cg02311932 | Body;5'UTR;TSS200 | S_Shore |  |  |
|  | cg02685016 | TSS1500;Body;5'UTR;1stExon | Island |  |  |
|  | cg03078685 | 3'UTR | OpenSea |  |  |
|  | cg04876424 | TSS1500 | N_Shore |  |  |
|  | cg05886811 | TSS1500 | Island |  |  |
|  | cg07346310 | TSS1500;Body;5'UTR;1stExon | S_Shore |  |  |
|  | cg09535605 | TSS1500 | Island |  |  |
|  | cg10096145 | TSS1500;1stExon | Island |  |  |
|  | cg10677246 | TSS1500 | Island |  |  |
|  | cg11084086 | TSS1500;TSS200 | Island |  |  |
|  | cg13868347 | TSS1500 | Island |  |  |
|  | cg14466942 | TSS1500;Body;5'UTR | S_Shore |  |  |
|  | cg15168816 | TSS1500;Body;5'UTR | S_Shore |  |  |
|  | cg15753394 | Body;5'UTR;TSS200 | S_Shore |  |  |
|  | cg17497965 | TSS1500;Body;5'UTR | S_Shore |  |  |
|  | cg18691800 | TSS1500 | Island |  |  |
|  | cg22874893 | TSS1500;TSS200 | Island |  |  |
|  | cg23146346 | Body;5'UTR;TSS200 | S_Shore |  |  |
|  | cg23207527 | Body;1stExon | S_Shore |  |  |
|  | cg23984769 | TSS1500;TSS200 | Island |  |  |
|  | cg24329102 | Body | S_Shelf |  |  |
|  | cg25302957 | TSS1500;Body;TSS200 | Island |  |  |
| ITGA8 | cg00198603 | Body | N_Shore | hsa-miR-1269a | 3'UTR |
|  | cg01078989 | 1stExon | Island |  |  |
|  | cg04578744 | Body | OpenSea |  |  |
|  | cg05609218 | Body | N_Shore |  |  |
|  | cg07073391 | TSS200 | Island |  |  |
|  | cg08361126 | TSS1500 | Island |  |  |
|  | cg11601663 | Body | OpenSea |  |  |
|  | cg13291704 | Body | Island |  |  |
|  | cg13492340 | TSS1500 | S_Shore |  |  |
|  | cg14364212 | Body | N_Shore |  |  |
|  | cg16422098 | TSS200 | Island |  |  |
|  | cg16902509 | Body | N_Shore |  |  |
|  | cg17894577 | Body | OpenSea |  |  |
|  | cg17938623 | TSS1500 | S_Shore |  |  |
|  | cg19944763 | TSS200 | Island |  |  |
|  | cg23317474 | Body | OpenSea |  |  |
|  | cg24959335 | Body | N_Shore |  |  |
|  | cg25825488 | TSS200 | Island |  |  |
|  | cg26104297 | TSS1500 | Island |  |  |
|  | cg27321175 | Body | OpenSea |  |  |
| ZDHHC19 | cg02039400 | Body | N_Shore | hsa-miR-196a-5p | 3'UTR |
|  | cg03327494 | 1stExon | S_Shelf |  |  |
|  | cg04790636 | 3'UTR | OpenSea |  |  |
|  | cg07111415 | TSS200 | S_Shelf |  |  |
|  | cg08022543 | Body | OpenSea |  |  |
|  | cg12702506 | 5'UTR;1stExon | S_Shelf |  |  |
|  | cg14483431 | Body | Island |  |  |
|  | cg18442453 | Body | Island |  |  |
|  | cg20278854 | Body | OpenSea |  |  |
|  | cg22386920 | Body | Island |  |  |
|  | cg23052855 | Body | OpenSea |  |  |
|  | cg24517978 | Body | N_Shelf |  |  |
|  | cg26292150 | Body | Island |  |  |
| SRGN | cg02851793 | Body | OpenSea | hsa-miR-210-3p | 3'UTR |
|  | cg06041695 | Body | OpenSea |  |  |
|  | cg13445486 | TSS200 | OpenSea |  |  |
|  | cg13645409 | Body | OpenSea |  |  |
|  | cg14898243 | Body | OpenSea |  |  |
|  | cg17342283 | 1stExon;5'UTR | OpenSea |  |  |
|  | cg18278184 | TSS1500 | OpenSea |  |  |
|  | cg26522946 | TSS1500 | OpenSea |  |  |
|  | cg27208307 | TSS1500 | OpenSea |  |  |
| S1PR1 | cg02628801 | TSS1500 | Island | hsa-miR-1269b | 3'UTR |
|  | cg03050965 | Body | S_Shelf |  |  |
|  | cg04156369 | 5'UTR;1stExon | Island |  |  |
|  | cg04701420 | TSS1500 | N_Shore |  |  |
|  | cg05053688 | 5'UTR | Island |  |  |
|  | cg05160678 | TSS1500 | N_Shore |  |  |
|  | cg05338680 | TSS1500 | Island |  |  |
|  | cg07400091 | 5'UTR | S_Shore |  |  |
|  | cg10020333 | 5'UTR | S_Shore |  |  |
|  | cg10210739 | 5'UTR | S_Shore |  |  |
|  | cg13477354 | Body | S_Shelf |  |  |
|  | cg17859448 | 5'UTR | S_Shore |  |  |
|  | cg17972213 | Body | S_Shelf |  |  |
|  | cg18323466 | 5'UTR;1stExon | Island |  |  |
|  | cg18757941 | 5'UTR | S_Shore |  |  |
|  | cg19038540 | TSS1500 | N_Shore |  |  |
|  | cg20166532 | 5'UTR | S_Shore |  |  |
|  | cg20740769 | TSS1500 | Island |  |  |
|  | cg21385047 | 5'UTR | Island |  |  |
|  | cg23978968 | Body | S_Shore |  |  |
|  | cg25012864 | TSS1500 | Island |  |  |
| SLC6A5 | cg00007644 | Body | Island | hsa-miR-127-5p | 3'UTR |
|  | cg03479705 | Body | OpenSea |  |  |
|  | cg03573068 | TSS1500 | S_Shore |  |  |
|  | cg04032066 | Body | Island |  |  |
|  | cg04569615 | Body | N_Shore |  |  |
|  | cg04592728 | TSS1500 | S_Shore |  |  |
|  | cg04642741 | TSS200 | N_Shore |  |  |
|  | cg04968806 | Body | S_Shelf |  |  |
|  | cg05399320 | Body | S_Shore |  |  |
|  | cg05572763 | Body | OpenSea |  |  |
|  | cg05645677 | 1stExon;5'UTR | N_Shore |  |  |
|  | cg06804921 | TSS200 | N_Shore |  |  |
|  | cg08530317 | TSS200 | N_Shore |  |  |
|  | cg08828816 | 3'UTR | OpenSea |  |  |
|  | cg09136245 | Body | OpenSea |  |  |
|  | cg09357935 | Body | S_Shelf |  |  |
|  | cg10841552 | Body | Island |  |  |
|  | cg11288764 | TSS1500 | Island |  |  |
|  | cg11784785 | TSS1500 | S_Shore |  |  |
|  | cg12101354 | Body | N_Shore |  |  |
|  | cg13661968 | Body | N_Shore |  |  |
|  | cg14524936 | Body | OpenSea |  |  |
|  | cg15083015 | Body | S_Shelf |  |  |
|  | cg15828364 | Body | S_Shelf |  |  |
|  | cg17424999 | Body | Island |  |  |
|  | cg18042724 | Body | S_Shelf |  |  |
|  | cg18855426 | Body | OpenSea |  |  |
|  | cg18954388 | Body | N_Shore |  |  |
|  | cg19172170 | Body | OpenSea |  |  |
|  | cg20632573 | Body | N_Shore |  |  |
|  | cg21591624 | Body | Island |  |  |
|  | cg21957058 | Body | S_Shelf |  |  |
|  | cg23745839 | Body | S_Shelf |  |  |
|  | cg24420041 | TSS1500 | S_Shore |  |  |
|  | cg26213155 | Body | N_Shore |  |  |
|  | cg27036111 | TSS1500 | S_Shore |  |  |
| NR5A2 | cg00001583 | Body | Island | hsa-miR-127-5p | 3'UTR |
|  | cg00026457 | Body | Island |  |  |
|  | cg00940433 | 3'UTR | OpenSea |  |  |
|  | cg01333884 | Body | Island |  |  |
|  | cg01941755 | Body | Island |  |  |
|  | cg02457750 | Body | N_Shore |  |  |
|  | cg02773945 | Body | Island |  |  |
|  | cg02779944 | Body | Island |  |  |
|  | cg02901753 | Body | Island |  |  |
|  | cg03257172 | Body | N_Shore |  |  |
|  | cg04025964 | Body | Island |  |  |
|  | cg04097639 | Body | Island |  |  |
|  | cg04308769 | Body | N_Shore |  |  |
|  | cg04605987 | Body | N_Shore |  |  |
|  | cg05366139 | Body | OpenSea |  |  |
|  | cg05391318 | Body | Island |  |  |
|  | cg05470502 | Body | Island |  |  |
|  | cg06244002 | Body | Island |  |  |
|  | cg06880420 | Body | S_Shelf |  |  |
|  | cg07208853 | Body | S_Shore |  |  |
|  | cg07792529 | Body | OpenSea |  |  |
|  | cg07926895 | Body | S_Shore |  |  |
|  | cg08158862 | Body | S_Shore |  |  |
|  | cg10098523 | Body | N_Shelf |  |  |
|  | cg10513702 | Body | N_Shore |  |  |
|  | cg10972873 | Body | N_Shore |  |  |
|  | cg11219485 | Body | N_Shore |  |  |
|  | cg11800251 | Body | Island |  |  |
|  | cg12730323 | Body | Island |  |  |
|  | cg13229857 | Body | S_Shore |  |  |
|  | cg13476077 | Body | N_Shore |  |  |
|  | cg14025556 | Body | Island |  |  |
|  | cg15062377 | Body | N_Shore |  |  |
|  | cg15130541 | Body | Island |  |  |
|  | cg15198094 | Body | N_Shore |  |  |
|  | cg16046444 | Body | N_Shore |  |  |
|  | cg16993043 | Body | N_Shore |  |  |
|  | cg17486263 | Body | Island |  |  |
|  | cg17520027 | Body | Island |  |  |
|  | cg17716724 | Body | S_Shore |  |  |
|  | cg17759535 | Body | N_Shore |  |  |
|  | cg17804356 | Body | Island |  |  |
|  | cg17873998 | Body | Island |  |  |
|  | cg18126097 | Body | S_Shelf |  |  |
|  | cg18203366 | Body | Island |  |  |
|  | cg18394216 | Body | OpenSea |  |  |
|  | cg19713947 | Body | S_Shore |  |  |
|  | cg20406878 | TSS1500 | OpenSea |  |  |
|  | cg21540765 | Body | Island |  |  |
|  | cg21825944 | Body | N_Shelf |  |  |
|  | cg21851672 | Body | N_Shore |  |  |
|  | cg22515278 | Body | Island |  |  |
|  | cg23258678 | Body | N_Shore |  |  |
|  | cg23455785 | Body | Island |  |  |
|  | cg23806259 | Body | OpenSea |  |  |
|  | cg23996829 | Body | OpenSea |  |  |
|  | cg24157531 | Body | N_Shore |  |  |
|  | cg24352938 | Body | Island |  |  |
|  | cg25306277 | Body | N_Shore |  |  |
| CARTPT | cg01187920 | 1stExon | Island | hsa-miR-21-3p | 3'UTR |
|  | cg02762440 | TSS200 | N_Shore |  |  |
|  | cg08351336 | TSS200 | N_Shore |  |  |
|  | cg14999396 | Body | S_Shore |  |  |
|  | cg18250028 | Body | Island |  |  |
|  | cg20798152 | TSS200 | N_Shore |  |  |
|  | cg22212873 | 3'UTR | S_Shore |  |  |
|  | cg22517388 | TSS200 | Island |  |  |
|  | cg23300372 | Body | Island |  |  |
|  | cg26146287 | TSS1500 | N_Shore |  |  |
|  | cg26371061 | TSS200 | N_Shore |  |  |
| TRIM71 | cg00997054 | Body | OpenSea | hsa-miR-1269a | 3'UTR |
|  | cg00997338 | Body | N_Shore |  |  |
|  | cg01553090 | Body | OpenSea |  |  |
|  | cg02406098 | Body | OpenSea |  |  |
|  | cg04507915 | Body | Island |  |  |
|  | cg06379532 | Body | OpenSea |  |  |
|  | cg06651450 | Body | N_Shore |  |  |
|  | cg08145617 | TSS1500 | Island |  |  |
|  | cg12338417 | 1stExon | Island |  |  |
|  | cg13000260 | Body | OpenSea |  |  |
|  | cg13896818 | Body | S_Shore |  |  |
|  | cg14560430 | Body | Island |  |  |
|  | cg14657525 | TSS1500 | Island |  |  |
|  | cg14776998 | Body | S_Shore |  |  |
|  | cg15630071 | Body | Island |  |  |
|  | cg15923864 | Body | OpenSea |  |  |
|  | cg17029062 | TSS200 | Island |  |  |
|  | cg17980786 | 3'UTR | OpenSea |  |  |
|  | cg18170070 | Body | OpenSea |  |  |
|  | cg18249634 | Body | Island |  |  |
|  | cg18310412 | TSS1500 | Island |  |  |
|  | cg18415444 | Body | OpenSea |  |  |
|  | cg19127283 | TSS200 | Island |  |  |
|  | cg19200592 | Body | OpenSea |  |  |
|  | cg19741945 | 1stExon | Island |  |  |
|  | cg21124497 | TSS200 | Island |  |  |
|  | cg21484228 | TSS200 | Island |  |  |
|  | cg21654379 | Body | S_Shore |  |  |
|  | cg23243038 | 5'UTR;1stExon | Island |  |  |
|  | cg23528400 | TSS200 | Island |  |  |
|  | cg23666945 | Body | Island |  |  |
|  | cg24348110 | Body | N_Shore |  |  |
|  | cg24629438 | Body | OpenSea |  |  |
|  | cg24847757 | Body | OpenSea |  |  |
|  | cg24953321 | Body | N_Shore |  |  |
|  | cg25732410 | Body | OpenSea |  |  |
|  | cg27093242 | Body | OpenSea |  |  |
|  | cg27195956 | Body | N_Shore |  |  |
|  | ch.3.680450R | Body | OpenSea |  |  |
| CFTR | cg00735923 | TSS200 | OpenSea | hsa-miR-767-5p | 3'UTR |
|  | cg06081199 | 5'UTR;1stExon | OpenSea |  |  |
|  | cg09181792 | TSS1500 | OpenSea |  |  |
|  | cg09341015 | Body | OpenSea |  |  |
|  | cg09626894 | TSS1500 | OpenSea |  |  |
|  | cg17204129 | TSS1500 | OpenSea |  |  |
|  | cg17616554 | TSS1500 | OpenSea |  |  |
|  | cg21212505 | Body | OpenSea |  |  |
|  | cg21461649 | TSS200 | OpenSea |  |  |
|  | cg22467052 | Body | OpenSea |  |  |
|  | cg22533025 | Body | OpenSea |  |  |
|  | cg25509184 | TSS1500 | OpenSea |  |  |
|  | cg26635219 | TSS1500 | OpenSea |  |  |
| ST8SIA6 | cg01466017 | TSS1500 | Island | hsa-miR-105-5p | 3'UTR |
|  | cg02946934 | TSS1500 | Island |  |  |
|  | cg05576619 | Body | OpenSea |  |  |
|  | cg09301734 | Body | Island |  |  |
|  | cg09571858 | 1stExon | Island |  |  |
|  | cg15019721 | TSS1500 | Island |  |  |
|  | cg15468564 | Body | N_Shelf |  |  |
|  | cg17256364 | Body | N_Shore |  |  |
|  | cg20011402 | 1stExon | Island |  |  |
|  | cg23284074 | Body | OpenSea |  |  |
|  | cg27475522 | TSS1500 | S_Shore |  |  |
| CPEB1 | cg00317577 | Body | OpenSea | hsa-miR-183-5p | 3'UTR |
|  | cg01645753 | TSS200 | Island |  |  |
|  | cg01776825 | Body | OpenSea |  |  |
|  | cg03180980 | Body;TSS200 | OpenSea |  |  |
|  | cg03340215 | Body | Island |  |  |
|  | cg04140663 | Body | Island |  |  |
|  | cg04184836 | 5'UTR;1stExon | Island |  |  |
|  | cg04781638 | Body;TSS1500 | OpenSea |  |  |
|  | cg04807004 | Body;TSS200 | OpenSea |  |  |
|  | cg05874309 | Body | OpenSea |  |  |
|  | cg06263843 | Body;5'UTR;1stExon | OpenSea |  |  |
|  | cg07386898 | Body | Island |  |  |
|  | cg07624612 | Body | N_Shelf |  |  |
|  | cg07769790 | TSS1500 | Island |  |  |
|  | cg08084502 | Body;TSS200 | OpenSea |  |  |
|  | cg08688548 | TSS1500 | Island |  |  |
|  | cg09400152 | Body | OpenSea |  |  |
|  | cg10140906 | Body;TSS200 | OpenSea |  |  |
|  | cg11022060 | Body | OpenSea |  |  |
|  | cg11074968 | Body;TSS1500 | OpenSea |  |  |
|  | cg11871991 | Body;1stExon | OpenSea |  |  |
|  | cg16043144 | Body | Island |  |  |
|  | cg16495212 | TSS1500 | Island |  |  |
|  | cg17453840 | TSS1500 | Island |  |  |
|  | cg19665362 | TSS200 | Island |  |  |
|  | cg24319381 | TSS200 | Island |  |  |
|  | cg24689264 | Body | OpenSea |  |  |
|  | cg25513800 | Body | N_Shore |  |  |
|  | cg25856663 | TSS1500 | S_Shore |  |  |
|  | cg26565719 | Body | OpenSea |  |  |
|  | cg27578811 | Body | Island |  |  |
| HLF | cg01185682 | Body | S_Shore | hsa-miR-183-5p | 3'UTR |
|  | cg01392772 | 5'UTR;1stExon | Island |  |  |
|  | cg01451391 | Body | S_Shore |  |  |
|  | cg02383154 | Body | S_Shore |  |  |
|  | cg04219321 | TSS1500 | N_Shore |  |  |
|  | cg04795713 | 5'UTR;1stExon | Island |  |  |
|  | cg05452524 | Body | Island |  |  |
|  | cg14399851 | 5'UTR;1stExon | Island |  |  |
|  | cg15833843 | TSS1500 | N_Shore |  |  |
|  | cg17254383 | Body | OpenSea |  |  |
|  | cg18608206 | 3'UTR | OpenSea |  |  |
|  | cg20640281 | TSS1500 | N_Shore |  |  |
|  | cg25534244 | TSS1500 | N_Shore |  |  |
|  | cg25826457 | 5'UTR;1stExon | Island |  |  |
| PIEZO2 | cg00335802 | Body | OpenSea | hsa-let-7c-5p | 5'UTR |
|  | cg00525823 | Body | Island |  |  |
|  | cg02664349 | TSS1500 | Island |  |  |
|  | cg03117976 | TSS1500 | Island |  |  |
|  | cg03426225 | Body | OpenSea |  |  |
|  | cg03602280 | TSS200 | Island |  |  |
|  | cg03686593 | TSS1500 | Island |  |  |
|  | cg05331731 | Body | N_Shore |  |  |
|  | cg06268875 | Body | N_Shore |  |  |
|  | cg06510438 | TSS1500 | Island |  |  |
|  | cg10023530 | TSS200 | Island |  |  |
|  | cg10567810 | TSS1500 | Island |  |  |
|  | cg12049992 | Body | N_Shore |  |  |
|  | cg12673429 | TSS1500 | S_Shore |  |  |
|  | cg12951849 | TSS200 | Island |  |  |
|  | cg13429270 | Body | S_Shore |  |  |
|  | cg14570051 | Body | OpenSea |  |  |
|  | cg15648792 | Body | N_Shelf |  |  |
|  | cg16969586 | Body | Island |  |  |
|  | cg20479672 | Body | S_Shore |  |  |
|  | cg20851748 | Body | N_Shore |  |  |
|  | cg21050234 | Body | Island |  |  |
|  | cg21165219 | Body | OpenSea |  |  |
|  | cg22374057 | 1stExon;5'UTR | Island |  |  |
|  | cg23556443 | TSS200 | Island |  |  |
|  | cg24362812 | TSS200 | Island |  |  |
|  | cg24879209 | Body | S_Shore |  |  |
| VWC2 | cg00333226 | 5'UTR | Island | hsa-miR-1307-5p | 3'UTR |
|  | cg01893212 | TSS200 | Island |  |  |
|  | cg02467990 | TSS200 | Island |  |  |
|  | cg03069122 | Body | Island |  |  |
|  | cg03577655 | Body | Island |  |  |
|  | cg04454951 | TSS1500 | Island |  |  |
|  | cg04904331 | TSS1500 | Island |  |  |
|  | cg05470523 | 5'UTR | Island |  |  |
|  | cg06528267 | Body | Island |  |  |
|  | cg07039180 | 5'UTR | Island |  |  |
|  | cg07379434 | 5'UTR | Island |  |  |
|  | cg07622201 | Body | OpenSea |  |  |
|  | cg09493505 | TSS200 | Island |  |  |
|  | cg12627948 | Body | OpenSea |  |  |
|  | cg14045872 | TSS200 | Island |  |  |
|  | cg17093995 | Body | Island |  |  |
|  | cg17161266 | Body | OpenSea |  |  |
|  | cg17538280 | TSS1500 | N_Shore |  |  |
|  | cg18206027 | 5'UTR;1stExon | Island |  |  |
|  | cg19365527 | 3'UTR | OpenSea |  |  |
|  | cg20066716 | Body | Island |  |  |
|  | cg21769093 | Body | S_Shelf |  |  |
|  | cg23436746 | Body | S_Shore |  |  |
|  | cg23877720 | Body | Island |  |  |
|  | cg26533595 | Body | OpenSea |  |  |
| PCDH17 | cg00208153 | 5'UTR;1stExon | N_Shore | hsa-miR-142-3p | 3'UTR |
|  | cg02994463 | Body | OpenSea |  |  |
|  | cg03865667 | TSS200 | N_Shore |  |  |
|  | cg04413320 | 1stExon | Island |  |  |
|  | cg07430472 | TSS1500 | N_Shore |  |  |
|  | cg07629125 | Body | OpenSea |  |  |
|  | cg07921625 | 1stExon | Island |  |  |
|  | cg11312408 | 1stExon | Island |  |  |
|  | cg12432236 | 1stExon | S_Shore |  |  |
|  | cg12606396 | TSS1500 | S_Shore |  |  |
|  | cg13806135 | TSS1500 | N_Shore |  |  |
|  | cg14427009 | 1stExon | Island |  |  |
|  | cg14893163 | 5'UTR;1stExon | N_Shore |  |  |
|  | cg15112032 | TSS1500 | S_Shore |  |  |
|  | cg19637591 | Body | OpenSea |  |  |
|  | cg20594465 | Body | OpenSea |  |  |
|  | cg22840780 | 1stExon | Island |  |  |
|  | cg24586528 | Body | OpenSea |  |  |
|  | cg25891355 | TSS1500 | N_Shore |  |  |
|  | cg26444528 | 1stExon | Island |  |  |
| FRMD3 | cg02603128 | Body | Island | hsa-miR-1269b | 3'UTR |
|  | cg03498697 | Body | N_Shore |  |  |
|  | cg11286584 | TSS200 | Island |  |  |
|  | cg13858974 | Body | N_Shelf |  |  |
|  | cg13977374 | Body | Island |  |  |
|  | cg14130664 | Body | N_Shore |  |  |
|  | cg21197678 | TSS1500 | Island |  |  |
|  | cg23492392 | Body | Island |  |  |
| C5orf38 | cg02509058 | Body | Island | hsa-miR-210-3p | 3'UTR |
|  | cg03570636 | Body | Island |  |  |
|  | cg04509074 | Body | Island |  |  |
|  | cg04992127 | TSS1500 | Island |  |  |
|  | cg05903444 | TSS1500 | Island |  |  |
|  | cg08204280 | TSS1500 | Island |  |  |
|  | cg08235864 | TSS1500 | Island |  |  |
|  | cg09524455 | TSS1500 | Island |  |  |
|  | cg10958362 | Body | Island |  |  |
|  | cg11337945 | 3'UTR | Island |  |  |
|  | cg11793269 | 1stExon | Island |  |  |
|  | cg12824782 | Body | Island |  |  |
|  | cg12860686 | Body | Island |  |  |
|  | cg13702053 | Body | Island |  |  |
|  | cg13944468 | Body | Island |  |  |
|  | cg18652721 | Body | Island |  |  |
|  | cg18693345 | Body | Island |  |  |
|  | cg19679633 | 5'UTR;1stExon | Island |  |  |
|  | cg21629500 | Body | Island |  |  |
|  | cg21999939 | Body | Island |  |  |
|  | cg23164183 | Body | Island |  |  |
|  | cg23248357 | 3'UTR | Island |  |  |
|  | cg26504021 | Body | Island |  |  |
|  | cg26932441 | Body | Island |  |  |
|  | cg27144115 | Body | Island |  |  |
| CRISP2 | cg01076129 | TSS200 | OpenSea | hsa-miR-195-5p | 5'UTR |
|  | cg01706515 | TSS1500 | OpenSea |  |  |
|  | cg04595372 | 5'UTR;1stExon | OpenSea |  |  |
|  | cg08942800 | TSS200 | OpenSea |  |  |
|  | cg12440062 | TSS200 | OpenSea |  |  |
|  | cg14997592 | 5'UTR | OpenSea |  |  |
|  | cg21710255 | TSS1500 | OpenSea |  |  |
|  | cg25390787 | TSS200 | OpenSea |  |  |
|  | cg26715042 | TSS200 | OpenSea |  |  |
| NFAM1 | cg00334863 | 3'UTR | OpenSea | hsa-miR-1269a | 3'UTR |
|  | cg01383955 | TSS200 | OpenSea |  |  |
|  | cg03017264 | TSS1500 | OpenSea |  |  |
|  | cg03365751 | 5'UTR;1stExon | OpenSea |  |  |
|  | cg07044422 | TSS200 | OpenSea |  |  |
|  | cg07264666 | TSS200 | OpenSea |  |  |
|  | cg07464578 | TSS1500 | OpenSea |  |  |
|  | cg09335713 | TSS200 | OpenSea |  |  |
|  | cg17568996 | Body | OpenSea |  |  |
|  | cg27098470 | TSS200 | OpenSea |  |  |
| DRC1 | cg01734112 | TSS200 | Island | hsa-miR-4529-3p | 5'UTR |
|  | cg02583525 | TSS200 | Island |  |  |
|  | cg04726446 | 1stExon | Island |  |  |
|  | cg10883621 | TSS200 | Island |  |  |
|  | cg11525280 | TSS200 | Island |  |  |
|  | cg12476443 | TSS1500 | N_Shore |  |  |
|  | cg13255216 | Body | Island |  |  |
|  | cg14013195 | TSS200 | Island |  |  |
|  | cg14273450 | 1stExon | Island |  |  |
| PCDH10 | cg00160440 | Body | S_Shelf | hsa-miR-378e | 5'UTR |
|  | cg00945238 | TSS1500 | Island |  |  |
|  | cg01408654 | TSS200 | Island |  |  |
|  | cg02043159 | TSS1500 | Island |  |  |
|  | cg02114924 | TSS1500 | Island |  |  |
|  | cg02562431 | TSS200 | Island |  |  |
|  | cg04035209 | TSS1500 | Island |  |  |
|  | cg05401965 | TSS1500 | Island |  |  |
|  | cg06667761 | 1stExon | Island |  |  |
|  | cg07665387 | TSS1500 | Island |  |  |
|  | cg09221867 | 1stExon | Island |  |  |
|  | cg09693004 | 1stExon | Island |  |  |
|  | cg09960109 | 1stExon | Island |  |  |
|  | cg10196720 | TSS1500 | Island |  |  |
|  | cg10524033 | TSS200 | Island |  |  |
|  | cg12746059 | TSS200 | Island |  |  |
|  | cg14146100 | TSS1500 | Island |  |  |
|  | cg14400886 | TSS200 | Island |  |  |
|  | cg14410319 | 5'UTR;1stExon | N_Shore |  |  |
|  | cg14795750 | 5'UTR;1stExon | N_Shore |  |  |
|  | cg17504999 | 1stExon | Island |  |  |
|  | cg19909865 | Body | S_Shore |  |  |
|  | cg23713176 | Body;1stExon;3'UTR | S_Shore |  |  |
|  | cg27600205 | TSS200 | Island |  |  |
| EIF4E3 | cg01228342 | 5'UTR;TSS1500 | OpenSea | hsa-miR-105-5p | 3'UTR |
|  | cg01295782 | Body | OpenSea |  |  |
|  | cg01454951 | 3'UTR | OpenSea |  |  |
|  | cg02995710 | TSS1500 | S_Shore |  |  |
|  | cg03024619 | 5'UTR | Island |  |  |
|  | cg03392679 | Body;5'UTR | Island |  |  |
|  | cg03457142 | TSS1500 | S_Shore |  |  |
|  | cg03604840 | 5'UTR | N_Shore |  |  |
|  | cg04087740 | TSS1500 | Island |  |  |
|  | cg04408595 | 5'UTR | Island |  |  |
|  | cg09429735 | Body;5'UTR | OpenSea |  |  |
|  | cg10172415 | TSS200;5'UTR | Island |  |  |
|  | cg13026730 | TSS1500 | Island |  |  |
|  | cg13562542 | 5'UTR | Island |  |  |
|  | cg14612785 | 5'UTR;TSS1500 | Island |  |  |
|  | cg18306788 | Body | OpenSea |  |  |
|  | cg18504632 | 3'UTR | OpenSea |  |  |
|  | cg20863339 | TSS200;TSS1500 | Island |  |  |
|  | cg22631938 | 5'UTR | N_Shore |  |  |
|  | cg22823146 | 5'UTR | Island |  |  |
|  | cg22888463 | 5'UTR;TSS1500 | OpenSea |  |  |
|  | cg23333146 | 3'UTR | OpenSea |  |  |
|  | cg24848973 | 5'UTR;TSS1500 | S_Shore |  |  |
|  | cg25649781 | Body | OpenSea |  |  |
| HHIP | cg02215603 | Body | OpenSea | hsa-miR-139-3p | 5'UTR |
|  | cg02524475 | TSS1500 | Island |  |  |
|  | cg07318204 | TSS1500 | Island |  |  |
|  | cg10203922 | TSS1500 | N_Shore |  |  |
|  | cg12936746 | TSS200 | Island |  |  |
|  | cg13150467 | 5'UTR;1stExon | Island |  |  |
|  | cg13749822 | TSS1500 | Island |  |  |
|  | cg14580567 | 5'UTR;1stExon | Island |  |  |
|  | cg14595911 | 5'UTR;1stExon | S_Shore |  |  |
|  | cg15319311 | Body | S_Shore |  |  |
|  | cg20263045 | Body | OpenSea |  |  |
|  | cg22511684 | Body | S_Shore |  |  |
|  | cg23109129 | Body | S_Shore |  |  |
|  | cg23802419 | Body | S_Shore |  |  |
|  | cg25703243 | Body | S_Shore |  |  |
|  | cg26339943 | Body | S_Shelf |  |  |
|  | cg26621699 | TSS1500 | Island |  |  |
|  | cg26978698 | 5'UTR;1stExon | S_Shore |  |  |
| TLL1 | cg00498604 | TSS1500 | N_Shore | hsa-miR-127-5p | 3'UTR |
|  | cg01606085 | TSS200 | N_Shore |  |  |
|  | cg05625362 | Body | Island |  |  |
|  | cg06202492 | Body | OpenSea |  |  |
|  | cg08570521 | 1stExon;5'UTR | N_Shore |  |  |
|  | cg08923494 | Body | S_Shelf |  |  |
|  | cg09669853 | 1stExon;5'UTR | Island |  |  |
|  | cg10756127 | Body | S_Shore |  |  |
|  | cg11678324 | TSS1500 | N_Shore |  |  |
|  | cg12836011 | Body | Island |  |  |
|  | cg15540138 | Body | S_Shore |  |  |
|  | cg18007463 | Body | S_Shore |  |  |
|  | cg19898128 | 1stExon;5'UTR | Island |  |  |
|  | cg20214477 | Body | S_Shore |  |  |
|  | cg20743881 | 3'UTR | OpenSea |  |  |
|  | cg21493633 | Body | S_Shore |  |  |
|  | cg21960859 | Body | S_Shore |  |  |
|  | cg23248150 | Body | S_Shore |  |  |
|  | cg24521633 | 1stExon;5'UTR | Island |  |  |
|  | cg26529911 | Body | S_Shore |  |  |
|  | ch.4.3045996R | Body | OpenSea |  |  |
| DAW1 | cg00063174 | TSS200 | Island | hsa-miR-378e | 5'UTR |
|  | cg01216370 | Body | Island |  |  |
|  | cg08034379 | TSS200 | Island |  |  |
|  | cg09638264 | TSS1500 | N_Shore |  |  |
|  | cg13808278 | 3'UTR | OpenSea |  |  |
|  | cg14329157 | TSS200 | N_Shore |  |  |
|  | cg14646075 | TSS1500 | N_Shore |  |  |
|  | cg20647610 | TSS200 | Island |  |  |
|  | cg21577049 | Body | Island |  |  |
|  | cg22079747 | TSS200 | N_Shore |  |  |
|  | cg23807890 | 5'UTR;1stExon | Island |  |  |

Supplementary Figure1. The protein-protein interaction (PPI) network for methylation-related DEGs. A, PPI network of hypermethylation and low-expression genes between cancer and adjacent samples from LUAD patients. B, PPI network of hypomethylation and high-expression genes between cancer and adjacent samples from LUAD patients.
