# Supplementary figures and images for: Epigenetic Alterations of DNA Methylation and miRNA Contribution to Lung Adenocarcinoma
Source: Front Genet. 2022 May 31;13:817552. doi: 10.3389/fgene.2022.817552 (PMC9194831; doi:10.3389/fgene.2022.817552)

**B**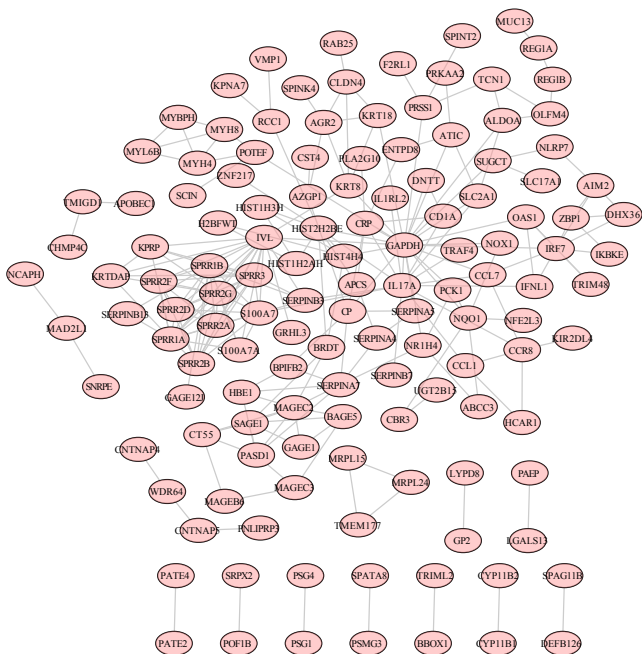

Supplement: Supplementary file 5 [file DataSheet1.PDF]
